# Exploring the lichen biota of Italy: New records from the Italian Alps and Northern Apennines

**DOI:** 10.3897/mycokeys.138.197797

**Published:** 2026-08-10

**Authors:** Chiara Pistocchi, Gabriele Gheza, Luca Di Nuzzo, Luana Francesconi, Silvia Girlanda, Josef Hafellner, Rasmus Kray, Helmut Mayrhofer, Pier Luigi Nimis, Holger Thüs, Juri Nascimbene

**Affiliations:** 1 BIOME Lab, Department of Biological, Geological and Environmental Sciences, University of Bologna, Via Irnerio 42, 40126 Bologna, Italy BIOME Lab, Department of Biological, Geological and Environmental Sciences, University of Bologna Bologna Italy; 2 Botanical Garden of Bergamo “Lorenzo Rota”, Passaggio Torre d’Adalberto 2, 24129 Bergamo, Italy Institute of Biology, University of Graz Graz Austria; 3 Institute of Biology, University of Graz, Holteigasse 6, 8010 Graz, Austria Department of Life Sciences, University of Trieste Trieste Italy; 4 Adlerstraße 12a, 79098 Freiburg, Germany Botanical Garden of Bergamo “Lorenzo Rota” Bergamo Italy; 5 Department of Life Sciences, University of Trieste, Via L. Giorgieri 10, 34127 Trieste, Italy Unaffiliated Freiburg Germany; 6 Botany Department, Stuttgart State Museum of Natural History, Rosenstein 1, 70191 Stuttgart, Germany Botany Department, Stuttgart State Museum of Natural History Stuttgart Germany

**Keywords:** Arctic-alpine lichens, biodiversity conservation, boreal-montane lichens, fieldwork, herbaria, lichen inventory, primary data

## Abstract

The onset of rapid climate change makes it urgent to intensify exploration of potentially threatened lichen biota, especially in mountain areas. In recent years we intensified field sampling in both the Alps and the Northern Apennines, and critically re-examined herbarium specimens from earlier campaigns to improve taxonomic resolution and occurrence records. This paper synthesizes those efforts, providing a robust empirical basis for assessing lichen dynamics in these mountain systems and for informing conservation priorities. Overall, we report 518 occurrences corresponding to 288 infrageneric taxa. Of these, 33 taxa are new to Italy and 255 are newly recorded for at least one Italian administrative region. For several taxa we supply integrated occurrence, ecological and molecular data that clarify aspects of their global distribution, and taxonomic circumscription. The high incidence of species with boreal-montane to arctic-alpine distributions in the Northern Apennines supports the hypothesis that these mountains act as southern outposts for elements of the arctic-boreal biota. Our results also highlight the enduring value of herbaria as repositories of primary biodiversity data and underscore the necessity of continued field exploration to keep species lists and distributional knowledge up to date in the face of accelerating environmental change.

## Introduction

The world’s mountainous regions are important biodiversity hotspots (Rahbek et al. 2019a), where interactions among biotic factors, climate and geology shape evolutionary trajectories and patterns of endemism (Rahbek et al. 2019b; Perrigo et al. 2020). These interactions produce complex spatial mosaics of habitat and microclimate that promote both speciation and the persistence of relict lineages. In the European context, the Alps exemplify this complexity: despite increasing human influence, many natural dynamics remain relatively well preserved, largely thanks to the network of protected areas established over the last century.

The present distribution of numerous alpine taxa bears the imprint of Quaternary glaciations (Rota et al. 2024). Glacial cycles facilitated the northward arrival and subsequent persistence of boreal and arctic species in cold, high-elevation environments, while simultaneously allowing thermophilous taxa to survive on warm, sheltered edges of peripheral ranges and to expand northwards following glacial retreat. These historical range shifts and refugial dynamics continue to determine contemporary patterns of species richness and turnover across elevational gradients.

The Northern Apennine chain, although its peaks rarely exceed 2,000 m, retains a pronounced arctic–boreal character: many cold-adapted species reach here their southernmost distribution limits (Tomaselli 1991; Tomaselli and Gualmini 2000; Petraglia and Tomaselli 2007). Biogeographically, this mountain chain lies at the interface between the Boreal and Mediterranean regions, producing a distinctive assemblage in which boreal relicts and Mediterranean elements coexist across short spatial scales. Such transitional settings are particularly important for understanding range-edge dynamics and the conservation of peripheral populations.

Among alpine organisms, lichens are both diverse and informative indicators of past and present environmental conditions. The lichen biota of the Alps is among the best explored globally, with over 3,000 taxa recorded to date (Nimis et al. 2018), a legacy rooted in a long tradition of lichenological study dating back to the 19^th^ century (e.g., the work of Ferdinand Arnold; Nascimbene et al. 2022). Italy contains a substantial portion of the Alpine system, from the Maritime Alps in Liguria to the Julian Alps in Friuli, and thus plays a central role in regional lichen diversity and biogeography.

Nevertheless, ongoing taxonomic revision and intensified field surveys reveal that our knowledge remains incomplete. Recent systematic work and targeted explorations (Gheza 2019; Nascimbene et al. 2021), including efforts associated with the Dolichens project (Francesconi et al. 2024, 2025a), indicate that many species remain undescribed or under-recorded and that the known distributions of numerous taxa are far from exhaustive. This taxonomic and distributional uncertainty hampers robust conservation assessments for many lichens. The gap in knowledge is particularly acute in the Northern Apennines, where lichenological sampling has historically been sparse (Fariselli et al. 2020), especially at high elevations, where cold-adapted biota may persist as relictual legacies of Quaternary glaciations.

The urgency of addressing these knowledge gaps is heightened by rapid climate change, which is progressing in mountain regions at rates approximately twice those observed in adjacent lowlands (Pepin et al. 2025). Accelerating warming, together with land-use change and other anthropogenic pressures, increases the risk that range-restricted and cold-adapted lichen species will lose suitable habitat before their distributions are fully documented. To respond to this challenge, we have intensified fieldwork in recent years, substantially increasing new collections, and have critically re-examined herbarium material from earlier campaigns in the light of recent taxonomic advances.

This paper summarizes the outcomes of that work. Our aims are to provide a more complete and taxonomically up-to-date account of lichen occurrences in the Italian Alps and Northern Apennines, to identify taxa and habitats of conservation concern, and to supply new occurrence data for species whose distribution, ecology and taxonomy remain poorly known. By establishing a firmer empirical basis for distributional and taxonomic knowledge, we intend to support more informed conservation planning and future studies of biogeographical and evolutionary dynamics in these mountain systems.

## Materials and methods

### Study area

**Figure 1.**
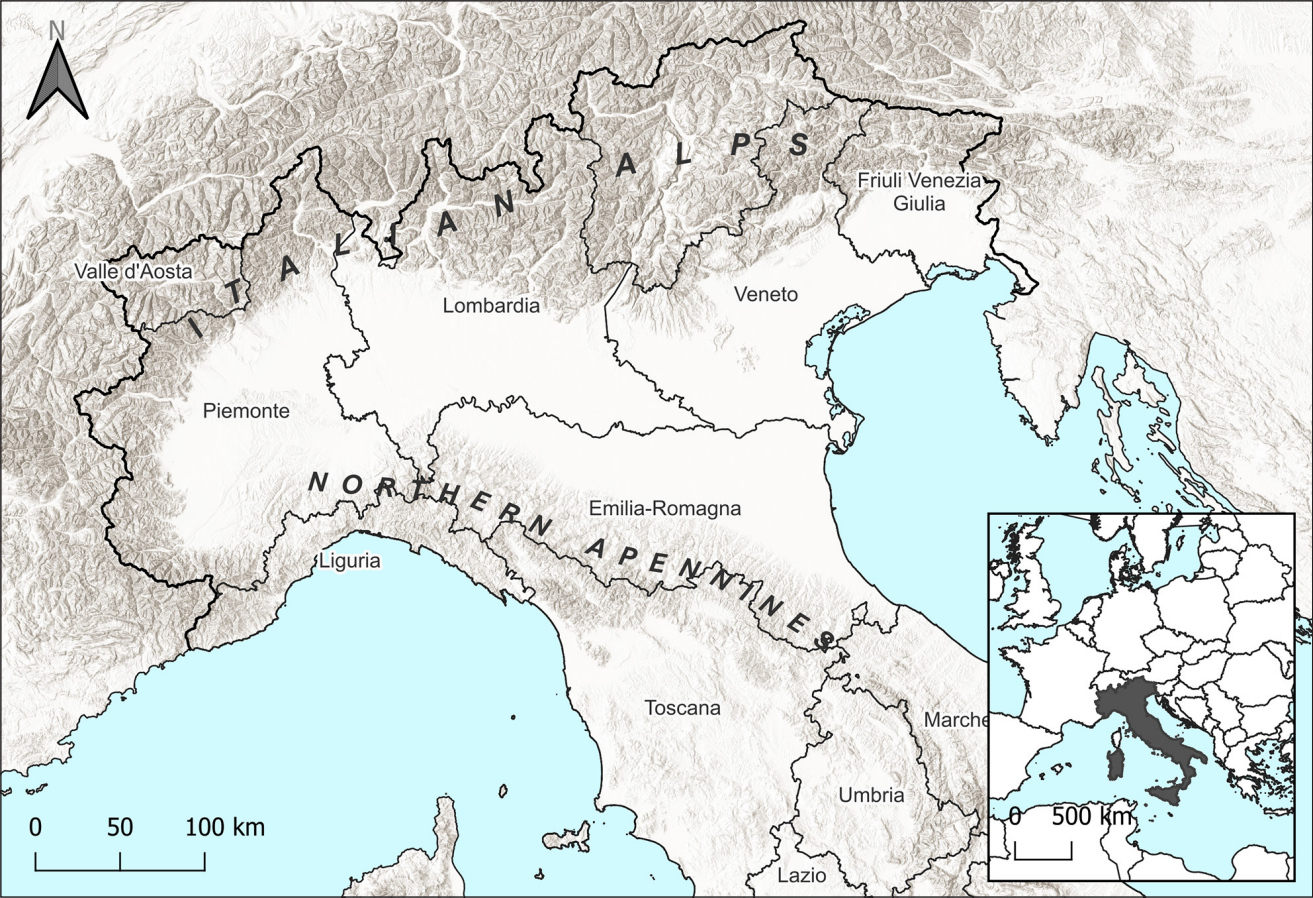
Administrative regions of northern Italy and the main mountain ranges (Italian Alps and Northern Apennines).

The study area comprises the parts of the Alps included in the Italian territory and the Northern Apennines, the northernmost mountain ranges of the Italian peninsula (Fig. 1).

The Italian Alps extend in a north-east to south-west direction from Colle di Cadibona to the Italian-Slovenian border, crossing the westernmost part of Liguria, Valle d’Aosta, Piemonte, Lombardia, Trentino-Alto Adige, Veneto and Friuli Venezia Giulia. Four National (Ntl) Parks (Gran Paradiso, Val Grande, Stelvio, and Dolomiti Bellunesi) and many other protected areas are included in this area. Geology is complex, including several different lithotypes: carbonate and clastic sedimentary rocks, metamorphic continental margin rocks, igneous intrusive rocks, metamorphic and ophiolitic oceanic rocks, whose distribution depends on the processes of Alpine orogenesis (Dal Piaz et al. 2003). The Italian Alps display a high degree of climatic diversity: based on temperature and precipitation regimes, alpine climate zones occur above tree line, associated with glaciers and alpine tundra; moist boreal climate zones with cool to cold winters characterize the oroboreal and subalpine belts; a cool temperate climate prevails in the montane belt, and scattered areas, especially the inner Alpine valleys, exhibit continental, semi-arid climatic conditions (Kottek et al. 2006; Rubel et al. 2017).

The Northern Apennines extend in a north-west to south-east direction from Colle di Cadibona, which separates them from the Alps, to Bocca Trabaria, on the border between Umbria and Marche. They include two Ntl Parks (Appennino Tosco-Emiliano, Foreste Casentinesi, Monte Falterona e Campigna) and cover the administrative regions of Liguria, Toscana, Emilia-Romagna and the northernmost part of Umbria and Marche. The main geological substrate is sandstone, with four distinct lithostratigraphic units which differ in chronology and carbonate content, producing acidic and subacidic soils (Dinelli et al. 1999; Conti et al. 2020). The Northern Apennines separate two distinct climatic regions: the Tyrrhenian region, which is affected by humid air masses coming from the west, and the Adriatic region, exposed to the Po Valley, influenced by continental winds from the Balkans (Nimis 2016). Differences in climate along the ridge are also influenced by the valleys’ orientation and topography relative to solar radiation (Federici and Mazzanti 1988). During Local Last Glacial Maximum in the middle and late Pleistocene, the Northern Apennines hosted more than 100 valley and mountain glaciers, covering more than 260 km^2^, (Giraudi 2004; Baroni et al. 2018). These glaciations have left visible traces in the landscape, characterized by relict glacial landforms and deposits (De Stefani 1883, 1887; Lowe 1992; Giraudi 2004; Compostella et al. 2014).

### Record collection and identification

This work includes specimens that were collected in the Italian Alps and/or in the Northern Apennines and that are newly reported for Italy, or at least for one of its administrative regions.

They were identified using a dissecting and a compound microscope. While routine chemical spot tests were performed for most specimens, standardized thin-layer chromatography (TLC) or HPTLC were used when necessary, following Orange et al. 2010 and Arup et al. 1993, respectively. In some cases, molecular data were retrieved for cryptic or poorly known taxa. In this case, total lichen DNA was isolated from dried thallus material using E.Z.N.A.® Plant & Fungal DNA Kit or Macherey Nagel Nucleo Plant Nucleo Spin II Mini Kit® according to the manufacturer’s protocol. DNA extracts were subjected to PCR using primer ITS1F (Gardes and Bruns 1993) and ITS4 (White et al. 1990) and in one case ITS1-LM (Myllys et al. 1999) and ITS4 to amplify the internal transcribed spacer region (ITS). Amplification of the ITS region was carried out using GoTaq ® G2 Flexi DNA Polymerase (Promega) or meridian bioscience MyTaq™ Red Mix. For both primer combination, PCR cycling parameters used for amplifying the ITS region were: initial denaturation 95 °C for 2 min, followed by 34 cycles of 95 °C for 30 s, 54 °C for 30 s, 72 °C for 1 min, and final elongation 72 °C for 5 min. Amplification products were purified using ExoSAP-IT™ Express. All products were sent to the Macrogen Inc. or Eurofins for Sanger sequencing. For DNA barcoding purposes, we generated nine ITS sequences. The identities of the newly generated sequences were evaluated using BLASTn searches against the NCBI database, considering both query coverage and percentage identity. All new sequences have been submitted to GenBank.

Nomenclature follows ITALIC 8.0, the information system on Italian Lichens (Nimis 2026) which is continuously updated online, as consulted on March 9, 2026.

## Results and discussion

Overall, 518 occurrences referring to 288 infrageneric taxa are reported here, predominantly from the Alps (259 taxa) and to a lesser extent from the Northern Apennines (37 taxa). The complete annotated list with locality details is provided in Suppl. material 1. About 57% of the taxa derive from data-mining and/or taxonomic revision of already available material (for example, specimens stored in GZU and TSB), while approximately 53% were newly collected during the last seven years. Thirty-three taxa are new to Italy and 255 taxa are newly recorded for at least one administrative region.

Since the publication of the first modern checklist of the lichens of Italy (Nimis 1993), lichenological exploration in Italy has steadily increased (see Nimis and Martellos 2003; Nimis 2016, 2026). Our work has contributed substantial regional additions: 91 taxa to Piemonte, 63 to Veneto, 51 to Trentino-Alto Adige, 40 to Liguria, 34 to Emilia-Romagna, 25 to Friuli Venezia Giulia, 21 to Lombardia, 18 to Valle d’Aosta, and 4 to Toscana. Thirty-eight taxa were new to the Italian Alps and 18 to the Northern Apennines, while 15 were new to the entire Apennine chain.

### Biogeographical implications

These results confirm that, despite a long-standing lichenological tradition in Italy (Nimis et al. 2018; Nascimbene et al. 2021, 2022), many species remain unrecorded in both the Italian Alps and the Northern Apennines. The apparent imbalance of sampling effort in our dataset, biased towards the Alps, partly reflects the legacy of nineteenth-century lichenological studies and explains the larger number of new regional records from that mountain system. By contrast, systematic exploration of the Northern Apennines has only recently resumed (Fariselli et al. 2020), yet our findings demonstrate considerable potential for further discoveries in that chain as well.

From a biogeographical perspective, our data considerably enlarge the known distributional range of many species along the Alpine chain, with several “eastern” or “western” Alpine species being actually present throughout the Alps (Suppl. material 1). Furthermore, the high incidence of species with boreal-montane to arctic-alpine distributions in the Northern Apennines supports the hypothesis that these mountains represent the southernmost biogeographical limits for a number of cold-adapted lichens, as has been shown for vascular plants (Petraglia and Tomaselli 2007). Several cold-adapted taxa recorded here, for example *Solorina crocea* and *Cladonia macroceras*, have persisted across decades (Tomaselli 1991; Dalle Vedove et al. 2002; Fariselli et al. 2020). Historical records, however, warn of potential local extinctions: *Alectoria ochroleuca*, collected in the Renaissance Period by Ulisse Aldrovandi (Stech et al. 2018), has not been confirmed by recent surveys. Most cold-adapted species reported here were collected close to mountain summits, which limits their ability to track ongoing climate warming and suggests a substantial extinction debt; even small future climatic shifts could precipitate local losses at the southern margins of their ranges.

### Habitat preferences and ecological observations

New records were associated with a range of well-defined substrates and microhabitats, including calcareous outcrops, shaded siliceous cliffs, old-growth montane forests and anthropogenic stone structures (Suppl. material 1). The substrate specificity observed for many species underlines the importance of fine-scale habitat heterogeneity in maintaining lichen diversity. Microclimatic variables such as humidity, insolation and snow cover emerged repeatedly as key determinants of local occurrence; where available, long-term climatic data and detailed microhabitat descriptions (see Materials and Methods) help to explain the co-occurrence of thermophilous and cryophilous taxa. These ecological patterns are consistent with earlier regional studies (e.g., Vallese et al. 2022; Francesconi et al. 2025b) and provide a rationale for prioritizing similar microhabitats in future surveys.

### Taxonomic and methodological considerations

Our study supplied occurrence, ecological and molecular data for several taxa whose global distribution, ecology and taxonomic circumscription remain poorly understood (Suppl. material 1). Notable examples include: recently described species such as *Lecanora albula* and *L. vocontia*, both recorded from a few localities in the Western Alps; *Leprocaulon calcicola*, previously known only from England and southern France; *Metamelanea umbonata*, described from Switzerland and also reported from Scandinavia; *Ramalina europaea*, apparently widespread in central Europe; and *Verrucaria hakulinenii*, previously known only from Finland. We also report re-evaluated taxa whose distributions were insufficiently known, for example *Aspicilia spermatomanes*, often confused with other species, and *Verrucaria anziana*, long treated as a synonym of *V. latebrosa*.

Several records concern taxonomically difficult groups. Examples include *Dermatocarpon miniatum* var. *cirsodes* within the *D. miniatum* complex, *Phaeophyscia erythrocardia* (a first report from the Alps, previously known from North America and the north-west Caucasus), and *Placynthium nigrum* f. *crustaceum* and *P. rosulans*, currently known from only a few Alpine localities. Cryptic diversity is also evident: the distribution of *Parmelia ernstiae* remains poorly understood because historical records may be hidden under those of *P. saxatilis* s.l., the same applies to *P. pinnatifida*, a cryptic species which has been often confused with *P. omphalodes*. Finally, enigmatic life-history traits are exemplified by *Cladonia luteoalba*, which is thought to begin its life cycle as a parasite on species of the *C. coccifera* group.

Accurate identification in many cases required an integrative approach combining morphology, secondary chemistry and, where possible, molecular data. Molecular sequencing proved decisive for delimiting several cryptic or recently diverged lineages; where molecular work was not feasible, careful micromorphological study and chemical spot tests were essential. We therefore emphasize the value of integrating multiple lines of evidence in lichen taxonomy and recommend that future work prioritize broader molecular sampling across the region to test hypotheses of cryptic diversity and to refine phylogeographical inferences.

### Conservation and monitoring implications

Several taxa newly recorded here are of conservation concern at regional or national levels. The discovery of small, isolated populations highlights the need for targeted conservation measures, including protection of critical substrates and microhabitats from quarrying, inappropriate afforestation, and recreational disturbance. We recommend that the newly documented localities be incorporated into regional monitoring schemes and red-list assessments, and that voucher specimens be deposited in accessible herbaria to facilitate future re-examination. Longitudinal monitoring at selected sites will be essential to clarify population trends and to assess the impacts of climate change and land-use shifts.

These results also underscore the continuing value of herbaria as repositories of primary data: critical re-examination of deposited material in the light of current taxonomic knowledge yielded numerous new regional records. At the same time, our findings stress the importance of sustained field exploration to maintain up-to-date species lists and distributional data. Such primary data are fundamental for conservation planning (Copperthwaite et al. 2026), for taxonomic progress, and for parameterising forecasting models that can improve predictive power (Steinke et al. 2025).

## Conclusions

The 518 occurrences and 288 taxa reported here substantially advance floristic knowledge of lichens in the Alps and the Northern Apennines. Our results demonstrate that systematic field surveys, combined with integrative taxonomic methods and critical herbarium revision, remain indispensable for documenting mountain biodiversity. The new records have direct implications for biogeography, taxonomy and conservation, and they identify clear priorities for future work: intensified exploration of under-surveyed sectors, expanded molecular sampling, and the integration of new data into regional databases and conservation assessments.

## Additional information

### Artificial Intelligence (AI) use

The authors accept full responsibility for the content of the manuscript, including the disclosure of any use of AI.

No AI tools were used in the preparation of this manuscript.

### Author ORCIDs

C. Pistocchi https://orcid.org/0009-0006-4126-790X

G. Gheza https://orcid.org/0009-0009-7524-8522

L. Di Nuzzo https://orcid.org/0000-0002-5558-9401

L. Francesconi https://orcid.org/0000-0003-1745-7069

J. Hafellner https://orcid.org/0009-0008-9857-1501

H. Mayrhofer https://orcid.org/0000-0002-9433-3847

P.L. Nimis https://orcid.org/0000-0003-3523-0183

H. Thüs https://orcid.org/0000-0002-8697-4572

J. Nascimbene https://orcid.org/0000-0002-9174-654X

### Data availability

All of the data that support the findings of this study are available in the main text or Supplementary Information.

## Supplementary materials

Supplementary material 1 List of the records of newly reported taxa Data type pdf Explanation note For each taxon, the following information is presented: 1. administrative region(s); 2. collection locality, longitude and latitude in DMS, elevation, and substrate; 3. collection date, collector(s) name(s) (Leg.), and Herbarium code. Institutional herbaria are abbreviated according to Index Herbariorum (Thiers 2016). Private herbaria are abbreviated with Hb. followed by the family name (e.g., Hb. Nascimbene); the inventory number, when available, is shown in brackets. 4. accession number of GenBank, if the specimen was subjected to molecular analysis.5. short note on ecology, distribution and/or taxonomy. https://binary.pensoft.net/file/1741863This dataset is made available under the Open Database License (http://opendatacommons.org/licenses/odbl/1.0/). The Open Database License (ODbL) is a license agreement intended to allow users to freely share, modify, and use this Dataset while maintaining this same freedom for others, provided that the original source and author(s) are credited. Chiara Pistocchi, Gabriele Gheza, Luca Di Nuzzo, Luana Francesconi, Silvia Girlanda, Josef Hafellner, Rasmus Kray, Helmut Mayrhofer, Pier Luigi Nimis, Holger Thüs, Juri Nascimbene
