# Supplementary material for: Exploring the lichen biota of Italy: New records from the Italian Alps and Northern Apennines
Source: MycoKeys. 2026 Aug 10;138:237–47. doi: 10.3897/mycokeys.138.197797 (PMC13482319; doi:10.3897/mycokeys.138.197797)
Supplement: Supplementary file 1 [file mycokeys-138-237-s001.pdf]

Exploring the lichen biota of Italy: new records from the Italian Alps and Northern Apennines

Chiara Pistocchi[1], Gabriele Gheza[1,2*], Luca Di Nuzzo[1], Luana Francesconi[1], Silvia Girlanda[1], Josef Hafellner[3], Rasmus Kray[4], Helmut Mayrhofer[3], Pier Luigi Nimis[5], Holger Thüs[6], and Juri Nascimbene[1]

[1] BIOME Lab, Department of Biological, Geological and Environmental Sciences, University of Bologna, Via Irnerio 42, 40126 Bologna, Italy
[2] Botanical Garden of Bergamo "Lorenzo Rota", Passaggio Torre d'Adalberto 2, 24129 Bergamo, Italy
[3] Institute of Biology, University of Graz, Holteigasse 6, 8010 Graz, Austria
[4] Adlerstraße 12a, 79098 Freiburg, Germany
[5] Department of Life Sciences, University of Trieste, Via L. Giorgieri 10, 34127 Trieste, Italy
[6] Botany Department, Stuttgart State Museum of Natural History, Rosenstein 1, 70191 Stuttgart, Germany
Corresponding author: Gabriele Gheza (gheza.gabriele@gmail.com)

Supplementary Material 1

List of the records of newly reported taxa.
For each taxon, the following information is presented:

1. administrative region(s);
2. collection locality, longitude and latitude in DMS, elevation, and substrate;
3. collection date, collector(s) name(s) (Leg.), and Herbarium code. Institutional herbaria are abbreviated according to Index Herbariorum (Thiers 2016). Private herbaria are abbreviated with Hb. followed by the family name (e.g., Hb. Nascimbene); the inventory number, when available, is shown in brackets.
4. accession number of GenBank, if the specimen was subjected to molecular analysis.
5. short note on ecology, distribution and/or taxonomy.

Taxa new to Italy

Aquacidia trachona (Ach.) Aptroot (Pilocarpaceae)
Lombardia • Bergamo, Orobie Bergamasche Natural Park, between Vilmaggiore and Baita Brandelegno, 46°00'28"N, 10°06'15"E, 1190 m, on siliceous sandstone, 30 December 2024, leg. et det. G. Gheza, Hb. Gheza (24123005).
A mostly sterile, pycnidiate species mainly growing on sheltered, often rain-protected surfaces of siliceous rocks in shaded and humid situations. Previous records from Italy were unreliable (Nimis 2016).

Aspicilia mauritii Hue (Megasporaceae)
Veneto • Belluno, Dolomiti Bellunesi Ntl Park, Pezedei, below Forcella del Zità Sud, 46°16'27"N, 12°09'13"E, 2285 m, on selciferous carbonatic rocks, 2 July 2024, leg. et det. J. Nascimbene, Hb. Nascimbene (9449).
Piemonte • Cuneo, Colle di Sampeyre just W of the Pass, 44°33'10"N, 7°06'55"E, 2330 m, on base-rich siliceous rocks, 26 July 2000, leg. P. L. Nimis & M. Tretiach (with J. Hafellner), det. P. L. Nimis, TSB (32864).

Valle d'Aosta • Aosta, Passo del Piccolo San Bernardo, 45°41'47"N, 6°53'55"E, 2050 m, on base-rich siliceous rocks, September 1997, leg. et det. P. L. Nimis, TSB (29443).
A silicicolous, often sterile, species that was known from a few localities in the Western Alps (Switzerland). It seems to be rare in the Alps.

Aspicilia spermatomanes (Nyl.) Maheu & Gillet (Megasporaceae)
Trentino-Alto Adige • Trento, San Martino di Castrozza, near Lago di Colbricon Inferiore, 46°17'04"N, 11°45'46"E, 1922 m, on rhyolite, 2 July 2025, leg. H. Oberhollenzer 7281, det. M. Bertrand, STU.
A silicicolous species described from the Pyrenees and recently re-evaluated (Bertrand and Roux 2013), whose distribution is insufficiently known: there are many records from the French Alps, but the species is likely to be widespread throughout the Alps, having often being confused with other species, especially *A. cinerea*.

Calogaya clauzadeana (Gaya) Nav.-Ros. & Cl. Roux (Teloschistaceae)
Veneto • Belluno, Dolomiti Bellunesi Ntl Park, Vette Feltrine near Passo Vette Grandi, 46°05'27"N, 11°50'34"E, 2050 m, on selciferous carbonatic rocks, 5 August 2024, leg. et det. J. Nascimbene, Hb. Nascimbene (9266).
This species is characterized by a salmon-coloured thallus and the growth on vertical or rain-protected surfaces of calcareous rocks. In the Alps it was so far only known from the southern part of the Western Alps (Nimis et al. 2018).

Cladonia luteoalba A. Wilson & Wheldon (Cladoniaceae)
Trentino-Alto Adige • Trento, Stelvio Ntl Park, Val del Monte, Pradacci, 46°20'35"N, 10°37'42"E, 1654 m, on soil above siliceous rocks, 23 August 2025, leg. C. Pistocchi, det. G. Gheza, Hb. Gheza (25121805). • Trento, Stelvio Ntl Park, Val di Rabbi, Tassé, 46°23'25"N, 10°51'52"E, 1105 m, on soil above siliceous rocks, 27 August 2025, leg. C. Pistocchi, det. G. Gheza, Hb. Nascimbene (10216).
An enigmatic species which is believed to begin its life cycle as a parasite on species of the *C. coccifera*-group (*C. coccifera* s. str., *C. borealis*, *C. straminea*), having the same chemistry of the host, but a distinctive morphology (Stenroos 1990, Černajová et al. 2022).

Cryptodiscus gloeocapsa (Nitschke ex Arnold) Baloch, Gilenstam & Wedin (Stictidaceae)
Trentino-Alto Adige • Bolzano, Bozener Porphyrplateau, ridge between Welschnofen (Nova Levante) and Eggen (Ega) W of the mountain Latemar, 46°25'20"N, 11°29'30"E, 1120 m, encrusting bryophytes over porphyry, 19 October 1975, leg. et det. J. Hafellner, GZU (JH1101). • Bolzano, *ibidem*, 46°25'10"N, 11°30'25"E, 1440 m, encrusting bryophytes over porphyry, 19 October 1975, leg. et det. J. Poelt, GZU. • Trento, Stelvio Ntl Park, Val de la Mare c. 6 km N above Cogolo, SE of Malga Prabòn, 46°24'30"N, 10°41'25"E, 1780 m, on soil of embankment along a forest road, 27 July 2006, leg. J. Hafellner (with L. Muggia, M. Tretiach & J. Nascimbene), det. J. Hafellner, GZU (JH87881).
In the Alps the species mostly grows on acidic soil of a few years' old embankments of secondary dirt forest roads in shaded situations, mostly in the montane to subalpine belt. In other parts of Europe, it is also found in other habitats with pioneer character, such as consolidated acidic sand dunes or recently disturbed soil. Co-occurring bryophytes and leafy hepatics are partly or entirely overgrown by the filmy crustose thallus on which deeply immersed, tiny, pale yellow to medium brown urceolate apothecia develop. The species is widely distributed in the Northern Hemisphere. In the Alps, it had so far been recorded from Austria and Switzerland (Nimis et al. 2018).

Dermatocarpon miniatum* var. *cirsodes (Ach.) Zahlbr. (Verrucariaceae)
Lombardia • Brescia, trail between Laveno di Lozio and the Big Bench, 45°58'56"N, 10°16'11"E, 970 m, on argillite, 4 January 2020, leg. G. Gheza, det. H. Thüs, Hb. Gheza (20010406).
This taxon belongs to the difficult group of *D. miniatum* s. lat. It is separated from other morphotaxa by a thicker thallus and a distinctly papillose lower surface. The taxon is accepted in the lichen checklists of Austria (Hafellner and Türk 2016) and France (Roux et al. 2025b), but – like all infraspecific taxa within the *D.*

miniatum aggregate – not in Heiðmarsson (2017), who nonetheless acknowledged that genetic and morphological variation within *D. miniatum* is much larger than in other accepted species of the genus, and that the taxonomy of this group is not yet settled.

Gyalecta kukriensis (Räsänen) Räsänen (Gyalectaceae)
Piemonte • Cuneo, western ridge of Mt. Nebin ca. 1 Km E of Colle di Sampeyre), 44°32'40''N, 7°08'20''E, 2380 m, on siliceous base-rich rocks (ophiolites), 26 July 2000, leg. P. L. Nimis & M. Tretiach (with J. Hafellner), det. P. L. Nimis, TSB (32955).
On base-rich siliceous rocks near or above tree line; also known from Austria, the Carpathians, and Scandinavia.

Lecanora albula (Nyl.) Hue (Lecanoraceae)
Trentino-Alto Adige • Trento, Paneveggio-Pale di San Martino Natural Park, Cavallazza Piccola, 46°16'51''N, 11°46'59''E, 2200 m, on rocks, 2 July 2025, leg. et det. M. Bertrand, Hb. Bertrand (8248).
An epilithic species of the subalpine belt in the Alps and Pyrenees, growing on rocks very poor in calcium carbonate and generally close to the ground. The specimen fits with Roux et al. (2022).

Lecanora biformis (Ramond) Clauzade & Cl. Roux (Lecanoraceae)
Veneto • Belluno, Arabba, below Forcella Porta Vescovo, 46°28'35''N, 11°52'08''E, 2380 m, on volcanic rocks, 1 August 2025, leg. et det. J. Nascimbene, Hb. Nascimbene (10288).
On siliceous rocks in upland areas; reported from a few localities in the Western Alps (France), but perhaps not recognized elsewhere.

Lecanora margacea Poelt (Lecanoraceae)
Trentino-Alto Adige • Trento, Paneveggio-Pale di San Martino Natural Park, between Passo Rolle and Cavallazza Piccola, 46°17'25''N, 11°47'13''E, 2120 m, on semi-shaded rhyolite, 2 July 2025, leg. et det. R. Kray, Hb. Kray (1359).
A species with a thallus of whitish, roundish to later indistinctly lobate squamules and sessile apothecia with lecanorine margin and brown disc. On calcareous schists and marly limestones, with a few records from the Eastern Alps (Nimis et al. 2018).

***Lecanora voconti*a** (Clauzade & Cl. Roux) Cl. Roux & M. Bertrand (Lecanoraceae)
Trentino-Alto Adige • Trento, Southern Torre di Neva, 46°09'33''N, 11°54'42''E, 2200 m, on carbonatic rocks (Dolomia Principale), 18 October 2014, leg. J. Nascimbene, det. M. Grube, Hb. Nascimbene (4827).
A recently-described species (Roux et al. 2022) known from a few localities in the Western Alps (France), growing on horizontal to inclined faces of more or less calciferous rocks.

Leprocaulon calcicola Earl.-Benn., Orange, Hitch & Mark Powell (Leprocaulaceae)
Lombardia • Brescia, Redond di Pescarzo, 46°02'14''N, 10°20'14''E, 602 m, on dirt above calcareous rock, 4 May 2024, leg. G. Gheza, det. G. Gheza & H. Mayrhofer, Hb. Gheza (24050406). • Como, ancient trails above Sala Comacina, 45°57'59''N, 9°09'51''E, 364 m, on limestone, 24 February 2026, leg. et det. G. Gheza, Hb. Gheza.
A recently-described leprose species, typically growing on dry or mortared walls made of calcareous stones, previously reported only from England and Southern France (Orange et al. 2017, Roux et al. 2025a).

Metamelanea umbonata Henssen (Lichinaceae)
Trentino-Alto Adige • Trento, Paneveggio-Pale di San Martino Natural Park, Val Canali, Sentiero del Cacciatore on a dripping rock wall, 46°13'51''N, 11°51'06''E, 1892 m, on carbonatic rocks, 5 August 2025, leg. et det. J. Nascimbene, Hb. Nascimbene (10224).
A rare species described from Switzerland and also known from Scandinavia, growing in seepage tracks on steeply inclined, damp faces of calciferous rocks.

Micarea rhabdogena (Norman) Hedl. (Pilocarpaceae)
Trentino-Alto Adige • Trento, Val Nagaoni, bewteen Rifugio Fonteghi and Rifugio Boz, Loc. "Buse" close to a stream bank, 46°08'01"N, 11°52'50"E, 1167 m, on bark of *Picea abies*, 5 July 2025, leg. et det. H. Oberhollenzer, STU (7260).
A boreal-temperate-montane/subalpine species with a single record so far from the Eastern Alps (Carinthia, Austria).

Peccania cernohorskyi (Servít) Schiman-Czeika, Czeika & Guttová (Phylliscaceae)
Veneto • Belluno, Dolomiti Bellunesi Ntl Park, S-facing slope of Col Dorin, 46°09'50"N, 12°00'43"E, 1975 m, on carbonatic soil, 18 July 2025, leg. et det. J. Nascimbene, Hb. Nascimbene (10474).
On thin layers of soil or directly on basic or calciferous siliceous rocks; apparently rare in Central Europe, also known from the Southern Alps (France), but on calcareous rocks (hence records in need of confirmation), often confused with Anema-species and perhaps more widespread.

Phaeophyscia erythrocardia (Tuck.) Essl. 1978 (Physciaceae)
Trentino-Alto Adige • Trento, Adamello-Brenta Natural Park, Val Nambrone, along the paved road between Rifugio Nambrone and Rifugio Cornisello, 46°12'22"N, 10°44'20"E, 1830 m, at the base of the trunk of *Salix appendiculata*, 28 July 2023, leg. C. Pistocchi, L. Francesconi & G. Gheza, det. H. Mayrhofer, Hb. Nascimbene (9684).
Emilia-Romagna • Modena, Alto Appennino Modenese Natural Park, Mt. Cimone, above Rifugio Ninfa, 44°12'34"N, 10°43'16"E, 1577 m, on bark of *Fagus*, 21 October 2022, leg. J. Nascimbene & F. Bottegoni, det. H. Mayrhofer, Hb. Nascimbene (9683, 9685).
This epiphytic species is characterized by a red-pigmented medulla due to the presence of skyrin and by *Physcia*-type ascospores. First described as *Physcia obscura* var. *erythrocardia* by Tuckerman (1860) from trees in North America, Kashiwadani (1975) introduced the new combination *Physcia erythrocardia*, providing a detailed description. Esslinger (1978) introduced the new combination *Phaeophyscia erythrocardia*, discussing the distinguishing characters concerning the most likely to be confused species *Ph. endococcinodes* and *Ph. endococcina.* The species is known from North America, with records from Minnesota and Mexico (Esslinger 1978), Iowa (Schutte 1983), and Alaska (McCune et al. 2018), and from the north-west Caucasus of Russia (Otte 2007). It is new to the Alps.

Placynthium nigrum* f. *crustaceum Hepp ex Walt. Watson (Placynthiaceae)
Veneto • Belluno, Dolomiti Bellunesi Ntl Park, Cimonega, below Bivacco Feltre close to a small waterfall, 46°10'01"N, 11°56'17"E, 1860 m, on dolomitic rocks, 19 June 2022, leg. et det. J. Nascimbene, Hb. Nascimbene (8006). • Belluno, Dolomiti Bellunesi National, Cimonega, above Bivacco Feltre close to a small waterfall, 46°10'12"N, 11°56'11"E, 1980 m, on dolomitic rocks, 19 June 2022, leg. et det. J. Nascimbene, Hb. Nascimbene (8007).
Trentino-Alto Adige • Trento, Adamello-Brenta Natural Park, near Passo del Grosté on a dripping rock wall, 46°12'06"N, 10°53'59"E, 2564 m, on dolomitic rocks, 1 July 2022, leg. et det. J. Nascimbene, Hb. Nascimbene (8008). • Trento, Adamello-Brenta Natural Park, Val Ambiez, between Rifugio Cacciatore and Rifugio Agostini, close to a spring, 46°08'23"N, 10°52'52"E, 2110 m, on carbonatic rocks, 23 August 2022, leg. et det. Juri Nascimbene, Hb. Nascimbene (8009).
This taxon, which is worthy of further study, has been recognized as different from the main form (Czeika and Czeika 2007), from which it also differs in ecology, being associated with semi-submerged environments at high elevations. For further details see also Thüs and Schultz (2009).

Placynthium rosulans (Th. Fr.) Zahlbr. (Placynthiaceae)
Lombardia • Bergamo, Orobie Bergamasche Natural Park, between Lago di Varro and Baita di Varro, 46°01'29"N, 10°06'24"E, 2120 m, on siliceous sandstone, 20 July 2024, leg. et det. G. Gheza & L. Di Nuzzo, Hb. Gheza (24072052).

Piemonte • Cuneo, cirque E below summit of Rocca dell'Abisso W of Col di Tenda NW below Fort de Giaure, 44°08'25''N, 7°31'05''E, 2250 m, on gneiss, 22 July 2000, leg. P. L. Nimis & M. Tretiach (with J. Hafellner), det. P. L. Nimis, TSB (34355).
A mainly northern species found on moist siliceous rocks, e.g., in inundation zones along brooks, near or above tree line; known from a few localities in the Alps, but perhaps more widespread. The relationship with *P. pannariellum* still needs further study.

Placynthium tantaleum (Hepp) Hue (Placynthiaceae)
Trentino-Alto Adige • Bressanone, Val di Valles, Rio di Valles, near the parking area "Hinterländle", 46°51'33''N, 11°37'16''E, 1390m, on inundated rocks, 7 July 2025, leg. et det. R. Kray, Hb. Kray (1407).
Piemonte • Cuneo, Marguareis Natural Park, just NW above the village Carnino, 44°08'56''N, 7°43'44''E, 1400 m, on slightly calciferous blue-green schists, 19 July 2000, leg. P. L. Nimis & M. Tretiach (with J. Hafellner), det. P. L. Nimis, TSB (33335).
On inundated or persistently wetted calcareous or siliceous rocks and boulders in or along mountain rivers and streams. The epithet has been earlier used for various forms of *P. nigrum*, but our sample complies with the description, and has broader, 2-celled spores.

Pleopyrenis picina (Nyl.) Clem. (Porocyphaceae)
Trentino-Alto Adige • Trento, Paneveggio-Pale di San Martino Natural Park, Cavallazza Piccola, in small creek at the path north of Lago di Colbricon, 46°17'29''N, 11°46'30''E, 1910 m, on siliceous rocks, 2 July 2025, leg. R. Kray & M. Schultz, det. M. Schultz, Hb. Kray (1377).
An inconspicuous crustose cyanolichens growing on periodically wet siliceous rocks in upland regions. According to Prieto et al. 2024 it is widely distributed in continental Europe (Pyrenees, Black Forest, northern Alps, Ore Mountains, Giant Mountains).

Protoblastenia laeta (Poelt) Kainz & Rambold (Psoraceae)
Veneto • Belluno, Dolomiti Bellunesi Ntl Park, Vette Feltrine, near Passo Vette Grandi, 46°05'27''N, 11°50'34''E, 2050 m, on selciferous carbonatic rocks, 5 August 2024, leg. et det. J. Nascimbene, Hb. Nascimbene (9507).
On shaded calcareous rocks, usually on steep, north-exposed rock faces; widespread in Europe but rare, with a few other records from the Eastern Alps (Austria, Germany).

Protoparmelia leproloma (R. Sant.) Rambold & Poelt (Parmeliaceae)
Piemonte • Cuneo, crest SW above Colle dell'Agnello, 44°40'58''N, 6°58'39''E, 2830 m, on calciferous schists, 25 July 2000, leg. P. L. Nimis & M. Tretiach (with J. Hafellner), det. P. L. Nimis, TSB (33178).
An arctic-alpine silicicolous species which starts the life-cycle on other lichens (*Lecidea paupercula*, more rarely *Sporastatia* and other crustose lichens), more widespread in Scandinavia than in the Alps, where it occurs above tree line. The species does not belong to *Protoparmelia* s. str.

Protoparmelia placentiformis (J. Steiner) Poelt (Parmeliaceae)
Liguria • Imperia, Alpi Liguri Natural Park, mountains N above Triora on the road to Col Garezzo, 44°02'50''N, 7°45'15''E, 1650 m, on sandstone, 21 July 2000, leg. P. L. Nimis & M. Tretiach (with J. Hafellner), det. P. L. Nimis, TSB (33429).
On sunny siliceous rock in dry upland areas, lichenicolous on *Lecaimmeria cupreoatra*. Described from arid parts of Asia; after the record in Macedonia, the Italian record is the second finding from Europe. This characteristic lichen most probably does not belong to *Protoparmelia* s. str.

Puttea margaritella (Hulting) S.Stenroos & Huhtinen (*incertae sedis*)
Trentino-Alto Adige • Bolzano, Val di Bráies (Pragser Tal) S of Monguelfo (Welsberg), c. 0.5 km N of Lago di Bráies (Pragser Wildsee), 46°42'20''N, 12°05'15''E, 1480 m, on rotten stumps, on *Ptilidium pulcherrimum* (Weber) Vain., 4 September 2002, leg. et det. J. Hafellner, GZU (JH87952).

This hepaticolous species shows a strong preference for *Ptilidium pulcherrimum* (Poelt and Döbbeler 1975). Exceptionally it has also been found colonizing *Blepharostoma trichophyllum* (L.) Dumort., *Lepidozia reptans* (L.) Dumort., *Bazzania trilobata* (L.) Gray and *Mylia taylorii* (Hook.) Gray. A realistic chance to find the species is by screening *Ptilidium pulcherrimum* mats. Such populations are frequently found spreading on crosscuts of moderately rotten stumps in managed montane coniferous forests. Thus, *Puttea margaritella* does not appear to be restricted to old-growth forests. The overall distribution has been roughly outlined by Stenroos et al. (2009). The so far published records suggest that the species is endemic to the European part of the Holarctic region. In the Alps, it has so far been recorded from Austria and Switzerland (Nimis et al. 2018).

Ramalina europaea Gasparyan, Sipman & Lücking (Ramalinaceae)
Veneto • Belluno, Dolomiti Bellunesi Ntl Park, Erera-Piani Eterni, Col Dorin, 46°09’50”N, 12°00’41”E, 1980 m, on selciferous carbonatic rocks, 18 July 2025, leg. J. Nascimbene (10266).
Trentino-Alto Adige • Trento, Val di Sole, Lago dei Caprioli, 46°17’26”N, 10°45’50”E, 1407 m, on siliceous rocks, 18 June 2025, leg. C. Pistocchi, det. C. Pistocchi & G. Gheza, Hb. Nascimbene (10265), GenBank Acc.Nr.: PZ094140.
A recently-described species, apparently widespread in Central Europe, found both on rocks and tree bark. Typical of base-rich or weakly calciferous rocks, mainly under overhangs. The specimen TSB 41126 cited in Italic 8.0 (Nimis 2026) is actually from Carinthia, but very close to the Italian border.

Staurothele solvens (Anzi) Zschacke **var. *intermedia*** Cl. Roux & Vivant (Verrucariaceae)
Trentino-Alto Adige • Trento, Adamello-Brenta Natural Park, Val Ambiez, between Rifugio Cacciatore and Rifugio Agostini, close to a spring, 46°08’23”N, 10°52’52”E, 2110 m, on carbonatic rocks, 23 August 2022, leg. et det. J. Nascimbene, Hb. Nascimbene (8000).
A taxon of mid to moderately high elevations in the mountains of SW Europe, including the Alps.

Synalissina isidiodes (Nyl. ex Arnold) M. Schultz & M. Prieto (Lichinellaceae)
Friuli Venezia Giulia • Udine, Sappada, Laghi d’Olbe, 46°35’50”N, 12°41’33”E, 2160 m, on carbonatic rocks, 8 November 2025, leg. et det. J. Nascimbene, Hb. Nascimbene (10475).
Trentino-Alto Adige • Trento, Paneveggio-Pale di San Martino Natural Park, Val Canali, Rio di Pradidali, eastern trail, 46°13’17”N, 11°52’23”E, 1250 m, on sunny dolomite, 4 July 2025, leg. et det. R. Kray, Hb. Kray (1380).
On periodically wetted surfaces of various types of rocks; widespread in Northern Europe, further south most frequent in the mountains; known from scattered localities throughout the Alps, outside the Italian territory.

Thelenella muscorum* var. *octospora (Nyl.) Coppins & Fryday (Thelenellaceae)
Piemonte • Cuneo, ridge SE above Colle Valcavera-Vallone dell’Arma, 44°22’40”N, 7°06’15”E, 2440 m, on calciferous schists, 23 July 2000, leg. P. L. Nimis & M. Tretiach (with J. Hafellner), det. P. L. Nimis, TSB (34009).
A mainly western lichen in Europe found on dying mosses on rocks and soil, mostly in upland areas. The record from the Italian Alps is somehow surprising.

Thelopsis lojkana (Poetsch ex Arnold) Nyl. (Stictidaceae)
Veneto • Belluno, Alpago, Mt. Dolada, path to Forcella Dolada, 46°11’48”N, 12°21’14”E, 1683 m, on carbonatic rocks, 30 December 2025, leg. et det. J. Nascimbene, Hb. Nascimbene (10242).
On vertical to underhanging faces of limestone; rare in the Central European mountains, with a few scattered records from the Alps. The material reported here was collected on a humid and shaded rock surface, together with *Gyalecta leucaspis* (A. Massal.) Kremp.

Verrucaria bryoctona (Th. Fr.) Orange (Verrucariaceae)

Trentino-Alto Adige • Trento, Adamello-Brenta Natural Park, Cima Nana, 46°17'01"N, 10°54'14"E, 2561 m, on terricolous bryophytes, 17 August 2022, leg. C. Pistocchi, det. C. Pistocchi & J. Nascimbene, Hb. Nascimbene (9505).
A pioneer, ephemeral species growing on base-rich soil and terricolous, dying bryophytes; widespread in Western Europe, with a few records from the Eastern Alps (Austria).

Verrucaria hakulinenii Pykälä & Myllys (Verrucariaceae)
Veneto • Belluno, Dolomiti Bellunesi Ntl Park, Alpi Feltrine, Cimonega group, small waterfall near the path, 46°10'23"N, 11°56'07"E, 2120 m, on dolomitic rocks, 9 October 2021, leg. J. Nascimbene, det. Holger Thüs, Hb. Nascimbene (7600), ITS-GenBank Acc.Nr: PZ053820. • Belluno, Dolomiti Bellunesi Ntl Park, Alpi Feltrine, Cimonega group, small waterfall near the path, 46°10'11"N, 11°56'10"E, 1980 m, on dolomitic rocks, 9 October 2021, leg. J. Nascimbene, det. Holger Thüs, Hb. Nascimbene (7601), ITS-GenBank Acc.Nr: PZ053821. • Belluno, near Passo Pordoi, close to a spring above the street, 46°29'07"N, 11°48'57"E, 2219 m, on carbonatic rocks, 8 August 2024, leg. et det. J. Nascimbene, Hb. Nascimbene (9471), ITS-GenBank Acc.Nr: PZ094138.
A recently-described freshwater lichen from carbonatic rock in Finland (Pykälä et al. 2024) which is difficult to separate from other species in the *V. hydrophila* group by morphology alone. Its separation from *V. floerkeana* (which has not yet been sequenced, and for which Pykälä et al., 2024 recognized that the existing type material may be heterogenous) may require further study, but amongst those taxa for which reference sequences are available it is readily identified by its distinctive ITS-sequence. Our records are the first reported outside Finland and may indicate a wider distribution on wet limestone in cold regions.

Verrucaria poeltii (Servít) Breuss (Verrucariaceae)
Veneto • Belluno, Pordoi, between Rifugio Vièl dal Pan and Rifugio Fredarola, 46°28'36"N, 11°49'19"E, 2458 m, on volcanic rocks, 8 August 2024, leg. et det. J. Nascimbene, Hb. Nascimbene (9484).
The record by Breuss and Berger (2012) included in Italic 8.0 (Nimis 2026) is actually from Austrian territory, but very close to the border. Therefore, our record from Veneto is the first one from Italy.

Taxa new to administrative regions of Italy

Acarospora freyi H. Magn. (Acarosporaceae)
Veneto • Belluno, Dolomiti Bellunesi Ntl Park, Vette Feltrine near Passo Vette Grandi, 46°05'27"N, 11°50'34"E, 2050 m, on selciferous carbonatic rocks, 5 August 2024, leg. et det. J. Nascimbene, Hb. Nascimbene (9272).
Probably overlooked and more widespread, both in the Alps and in the Apennines, this lichen starts the life-cycle on *Aspicilia candida* and *A. polychroma* on calciferous rocks which are at least partly decalcified on the surface, with optimum near and above tree line.

Acarospora gallica H. Magn. (Acarosporaceae)
Piemonte • Cuneo, Valle Varaita, ca. 4 km W of the village Sampeyre, slopes exposed to the S just W of Villar, 44°34'55"N, 7°08'05"E, 1120 m, on siliceous base-rich rocks, 25 July 2000, leg. P. L. Nimis & M. Tretiach (with J. Hafellner), det. P. L. Nimis, TSB (32673).
A probably holarctic species of base-rich, weakly calciferous siliceous substrata, such as calcareous sandstone, brick, and roofing tiles, usually at relatively low elevations; much overlooked or confused with other species and certainly more widespread in Italy.

Acarospora hospitans H. Magn. (Acarosporaceae)
Piemonte • Cuneo, Monviso Natural Park, N slopes of Monviso, W above Crissolo just W above Lago Fiorenza (S above Pian del Re), 44°41'50"N, 7°05'35"E, 2130 m, on ophiolitic rocks, growing on *Aspicilia* sp., 25 July 2021, leg. P. L. Nimis & M. Tretiach (with J. Hafellner), det. P. L. Nimis, TSB (35209).

On siliceous rocks, growing on the thalli of other crustose lichens, according to Roux (in litt.) exclusively on *Aspicilia* sp. Much overlooked, and in need of further study.

Acarospora irregularis H. Magn. (Acarosporaceae)
Veneto • Belluno, Arabba, N-facing slope of Porta Vescovo, start of the via ferrata, 46°28’24’’N, 11°52’24’’E, 2610 m, on volcanic rocks, 1 August 2025, leg. et det. J. Nascimbene, Hb. Nascimbene (10415).
A species known from central Europe (Czech Republic, Hungary and Slovakia), as well as Austria, Greece and Italy, which was often confused with *A. nitrophila* and related species. According to Knudsen and Kocourková (2016), *A. badiofusca* subsp. *badiorubra* Clauzade & Cl. Roux nom. illegit. is a synonym of this species. See also Knudsen et al. (2014).

Adelolecia pilati (Hepp) Hertel & Hafellner (Ramalinaceae)
Liguria • Imperia, Alpi Liguri Natural Park, mountains N above Triora on the road to Col Garezzo, 44°02’50’’N, 7°45’15’’E, 1650 m, on sandstone, 21 July 2000, leg. P. L. Nimis & M. Tretiach (with J. Hafellner), det. P. L. Nimis, TSB (33433).
An arctic-alpine, circumpolar species of steeply inclined to rain-sheltered surfaces of weathered, metal-rich metamorphic rocks seldom wetted by rain, from the upper montane to the nival belt; widespread in the Alps and also occurring in the high Mediterranean mountains.

Agonimia gelatinosa (Ach.) M. Brand & Diederich (Verrucariaceae)
Emilia-Romagna • Reggio Emilia, Appennino Tosco Emiliano Ntl Park, Mt. Cusna, between Rifugio Battisti and Il Passone, 44°15’54’’N, 10°24’58’’E, 1803 m, on soil, 8 July 2025, leg. C. Pistocchi, S. Girlanda & F. Zinato, det. J. Nascimbene, Hb. Nascimbene (10241).
A species growing on plant debris and mosses in dry calcareous grasslands, with optimum near tree line; in Italy it was reported from the Alps and central-southern Apennines. This record is the first one for the Northern Apennines.

Anema tumidulum P.M. Jørg., M. Schultz & Guttová (Lichinaceae)
Trentino-Alto Adige • Trento, Adamello-Brenta Natural Park, Val Ambiez, between Rifugio Cacciatore and Rifugio Agostini, 46°08’22’’N, 10°52’50’’E, 2115 m, on carbonatic rocks, 23 August 2023, leg. et det. J. Nascimbene, Hb. Nascimbene (8794).
This species seems to be fairly common in Central Europe (Jørgensen et al. 2013). It grows on steeply inclined, sunny surfaces of calcareous or basic siliceous rocks with periodical water seepage after rain, with optimum in upland areas.

Anzina carneonivea (Anzi) Scheid. var. ***carneonivea*** (*incertae sedis*)
Veneto • Belluno, Casera Razzo, 1 Km from the border between Friuli and Veneto, 46°28’42’’N, 12°36’50’’E, 1750 m, on bark of conifers, 1981, leg. et det. P. L. Nimis, TSB (17934)
On acidic substrata such as bark, especially of conifers, wood, plant debris, dying bryophytes, in the understory of upper montane moist forest, and among shrubs in the subalpine belt of the Alps.

Arthonia didyma Körb. (Arthoniaceae)
Emilia-Romagna • Bologna, Corno alle Scale Natural Park, beech forest near Passo della Porticciola, 44°07’46’’N, 10°49’23’’E, 1650 m, on bark of *Fagus sylvatica*, 22 October 2021, leg. et det. J. Nascimbene, Hb. Nascimbene (8031).
A cool-temperate species found on smooth, acid bark in humid areas, most frequent in the beech belt of Tyrrhenian Italy.

Arthonia fusca (A. Massal.) Hepp (Arthoniaceae)

Piemonte • Cuneo, western ridge of Mt. Nebin ca. 1 Km E of Colle di Sampeyre, 44°32’40”N, 7°08’20”E, 2380 m, on siliceous base-rich rocks, 26 July 2000, leg. P. L. Nimis & M. Tretiach (with J. Hafellner), det. P. L. Nimis, TSB (32906).
A holarctic species of base-rich rocks and mortar, most frequent on pebbles, but also on walls, roofing tiles etc.; in the eu-Mediterranean belt it is replaced by *A. calcicola*.

Arthrorhaphis vulgaris (Schaer.) Frisch, Y.Ohmura, Holien & Bendiksby (Arthrorhaphidaceae)
Emilia-Romagna • Modena, Alto Appennino Modenese Natural Park, between Rifugio Ninfa and Mt. Cimone, 44°12’18”N, 10°42’46”E, 1750 m, on soil, 21 October 2022, leg. G. Gheza, det. C. Fagiani & G. Gheza, Hb. Nascimbene (8797). • Reggio Emilia, Appennino Tosco-Emiliano Ntl Park, Mt. Prado, between Lago della Bargetana and Sella del Monte Prado, 44°15’17”N, 10°24’02”E, 1832 m, on soil, 11 July 2025, leg. C. Pistocchi, S. Girlanda & F. Zinato, det. S. Girlanda & C. Pistocchi, Hb. Nascimbene (10219). • Reggio Emilia, Appennino Tosco-Emiliano Ntl Park, between Sella del Monte Prado and Mt. Prado, 44°15’03”N, 10°24’06”E, 1976 m, on soil, 12 July 2025, leg. C. Pistocchi, S. Girlanda & F. Zinato, det. S. Girlanda & C. Pistocchi, Hb. Pistocchi (776). • Reggio Emilia, Appennino Tosco-Emiliano Ntl Park, below Sella del Casarola, 44°19’54”N, 10°12’19”E, 1913 m, on soil, 14 July 2025, leg. C. Pistocchi, S. Girlanda & F. Zinato, det. S. Girlanda & C. Pistocchi, Hb. Nascimbene (10133).
The revision by Frisch et al. (2022) showed that *A. citrinella* s. lat. can be subdivided into several species. *A. citrinella* s. str., characterized by a thallus entirely consisting of small loose to compact aggregations of granular soredia, growing on saxicolous bryophytes, while common in Northern Europe, appears to be much rarer in the Alps than the recently-described *A. vulgaris*. The latter has a thallus of discrete to confluent areoles, growing on soil, terricolous bryophytes and plant remains, or lichenicolous on *Baeomyces*. These are the first records for the Northern Apennines.

Aspicilia mashiginensis (Zahlbr.) Oxner (Megasporaceae)
Veneto • Belluno, Dolomiti Bellunesi Ntl Park, below Mt. Pelf, 46°13’38”N, 12°12’24”E, 2080 m, on selciferous carbonatic rocks, 28 July 2023, leg. et det. J. Nascimbene, Hb. Nascimbene (8562). • Belluno, Pordoi, between Rifugio Vièl dal Pan and Rifugio Fredarola, 46°28’34”N, 11°49’42”E, 2552 m, on volcanic rocks, 8 August 2024, leg. et det. J. Nascimbene, Hb. Nascimbene (9485).
On basic siliceous rocks, often on weakly calciferous schists, in humid-shaded situations near and above tree line, up to the nival belt; to be looked further throughout the Alps. For further information see Tretiach and Hafellner (2000).

Aspicilia grisea Arnold (Megasporaceae)
Trentino-Alto Adige • Trento, Adamello-Brenta Natural Park, Val d’Amola, Passo dei Quattro Cantoni, 46°12’20”N, 10°41’35”E, 2774 m, on tonalite, 18 August 2025, leg. C. Pistocchi, det. I. Zanei, L. Grazia & C. Pistocchi, Hb. Nascimbene (10375).
A chemically variable species (Roux and coll. 2014), found on siliceous rocks, sometimes also on pebbles, certainly more widespread in the Alps, but very much overlooked.

Atla alpina Savić & Tibell (Verrucariaceae)
Friuli Venezia Giulia • Udine, Natura 2000 Site-Mt. Auernig and Mt. Corona, area of Passo Pramollo (Naßfeldpaß) ca. 6 km N of Pontebba, Mt. Carnizza (Garnitzenberg) E above the pass, limestone cliffs on the ridge W of the summit, 46°33’35”N, 13°17’36”E, 1840 m, on Palaeozoic limestone, 6 August 2007, leg. et det. J. Hafellner, GZU (JH76194).
Veneto • Belluno, Dolomiti Bellunesi Ntl Park, Mt. Pelf, 46°13’42”N, 12°12’22”E, 2173 m, on humid carbonatic rocks, 28 July 2023, leg. et det. J. Nascimbene, Hb. Nascimbene (8261).
Under the dissecting microscope the strongly protruding ascomata exhibit a rugose surface of the ostiolar region which is a diagnostic character for this otherweise *Polyblastia*-like species (Savić and Tibell 2008). It grows preferably on low outcrops and small boulders of calcareous rocks thus profiting from a locally increased humidity as result of soil moisture and transpiration of the surrounding herbaceous vegetation. In the

Alps it can be found from the tree line ecotone upwards but the distribution is still badly documented (Hafellner 2010). Confirmed records in Italy are still very few. The distribution as mapped in ITALIC 8.0 is partly based on historical records of misidentified specimens of *Polyblastia theleodes* (now *Henrica theleodes* (Sommerf.) Savić, Tibell & Nav.-Ros.).

Bacidia herbarum (Stizenb.) Arnold (Ramalinaceae)
Veneto • Belluno, Pordoi, between Rifugio Vièl dal Pan and Rifugio Fredarola, 46°28'35"N, 11°49'29"E, 2492 m, on the basal part of *Salix* sp., 8 August 2024, leg. et det. J. Nascimbene, Hb. Nascimbene (9363).
A cool-temperate to arctic-alpine, probably circumpolar lichen found on plant remains and dying bryophytes on calciferous ground, more rarely on bark, with optimum in upland areas.

Baeomyces carneus (Retz.) Flörke (Baeomycetaceae)
Veneto • Belluno, Dolomiti Bellunesi Ntl Park, Cima de Zità Sud, 46°16'16"N, 12°09'24"E, 2540 m, on organic soil over selciferous carbonatic rocks, 2 July 2024, leg. et det. J. Nascimbene, Hb. Nascimbene (9277). • Belluno, Dolomiti Bellunesi Ntl Park, Mt. Talvena, 46°15'58"N, 12°09'21"E, 2542 m, on soil, 24 June 2025, leg. et det. J. Nascimbene, Hb. Nascimbene (9989).
A mainly boreal-montane, perhaps circumpolar lichen found on acid soils high in clay and on weathered siliceous rocks, with optimum near tree line.

Baeomyces placophyllus Ach. (Baeomycetaceae)
Emilia-Romagna • Bologna, Corno alle Scale, between Passo della Porticciola and Punta Sofia, 44°07'34"N, 10°49'29"E, 1756 m, on soil, 25 June 2025, leg. et det. C. Pistocchi, L. Francesconi & F. Zinato, Hb. Nascimbene (10140). • Bologna, Corno alle Scale Natural Park, below Punta Sofia, 44°07'28"N, 10°49'40"E, 1848 m, on sandy soil, 26 June 2025, leg. et det. C. Pistocchi, L. Francesconi & F. Zinato, Hb. Pistocchi (818). • Reggio Emilia, Appennino Tosco-Emiliano Ntl Park, below Cima dell'Angelo, 44°16'18"N, 10°25'03"E, 1891 m, on soil, 10 July 2025, leg. et det. C. Pistocchi, S. Girlanda & F. Zinato, Hb. Nascimbene (9892). • Reggio Emilia, Appennino Tosco-Emiliano Ntl Park, below Mt. Cusna, 44°17'21"N, 10°23'28"E, 2056 m, on soil, 10 July 2025, leg. et det. C. Pistocchi, S. Girlanda & F. Zinato, Hb. Nascimbene (9926). • Reggio Emilia, Appennino Tosco-Emiliano Ntl Park, between Lago della Bargetana and Sella del Monte Prado, 44°15'17"N, 10°24'04"E, 1815 m, on soil, 11 July 2025, leg. et det. C. Pistocchi, S. Girlanda & F. Zinato, Hb. Pistocchi (765). • Reggio Emilia, Appennino Tosco-Emiliano Ntl Park, between Sella del Monte Prado and Mt. Prado, 44°15'03"N, 10°24'06"E, 1976 m, on soil, 12 July 2025, leg. et det. C. Pistocchi, S. Girlanda & F. Zinato, Hb. Nascimbene (10122). • Reggio Emilia, Appennino Tosco-Emiliano Ntl Park, above Sella del Casarola towards Alpe di Succiso, 44°19'54"N, 10°12'11"E, 1953 m, on soil, 14 July 2025, leg. et det. C. Pistocchi, S. Girlanda & F. Zinato, Hb. Nascimbene (10131). • Reggio Emilia, Appennino Tosco-Emiliano Ntl Park, Alpe di Succiso (summit area), 44°19'58"N, 10°11'44"E, 2027 m, on soil, 14 July 2025, leg. et det. C. Pistocchi, S. Girlanda & F. Zinato, Hb. Pistocchi (747). • Modena, Alto Appennino Modenese Natural Park, below Mt. Cimone, above "Il Casotto" of the Aeronautica Militare, 44°11'47"N, 10°42'16"E, 1937 m, on plant debris, 5 August 2025, leg. et det. C. Pistocchi, S. Girlanda & F. Zinato, Hb. Pistocchi (800). • Modena, Alto Appennino Modenese Natural Park, Mt. Cimone, below Rifugio Pian Cavallaro, 44°12'18"N, 10°42'23"E, 1753 m, on soil, 5 August 2025, leg. et det. C. Pistocchi, S. Girlanda & F. Zinato, Hb. Pistocchi (807).
An arctic-alpine to boreal-montane, probably circumpolar lichen found on sandy-clay soil in open stands (e.g., montane-subalpine grasslands), often in moderately disturbed habitats, sometimes reaching the alpine belt. These are the first records for the Apennines.

Bellemerea diamarta (Ach.) Hafellner & Cl. Roux (Lecideaceae)
Friuli Venezia Giulia • Udine, Natura 2000 Site-Mt. Auernig and Mt. Corona, area of Passo Pramollo (Naßfeldpaß) ca. 6 km N of Pontebba, Mt. Carnizza (Garnitzenberg) E above the pass, conspicuous head of siliceous rocks somewhat S of the ridge W of the summit, 46°33'35"N, 13°17'44"E, 1870 m, on low outcrops of siliceous rocks (iron-rich schists), 6 August 2007, leg. et det. J. Hafellner, GZU (JH76775).

At the given locality the species has been found accompanied by *Lecidea silacea* (Hoffm.) Ach. and *Tremolecia atrata* (Ach.) Hertel, other lichens with a strong preference of metal-rich siliceous rocks. The species is widespread in the Alps, from the montane belt upwards (Nimis et al. 2018).

Bellemerea subcandida (Arnold) Hafellner & Cl. Roux (Lecideaceae)
Veneto • Belluno, Dolomiti Bellunesi Ntl Park, Vette Feltrine near Passo Vette Grandi, 46°05'27"N, 11°50'34"E, 2050 m, on selciferous carbonatic rocks, 5 August 2024, leg. et det. J. Nascimbene, Hb. Nascimbene (9269). • Belluno, Dolomiti Bellunesi Ntl Park, Pezedei, below Forcella del Zità Sud, 46°16'27"N, 12°09'13"E, 2285 m, on selciferous carbonatic rocks, 2 July 2024, leg. et det. J. Nascimbene, Hb. Nascimbene (9451).
A probably overlooked and certainly more widespread, characteristic lichen of base-rich, weakly calciferous siliceous rocks in the mountains, known from southern Europe (Alps, Pyrenees).

Biatora subduplex (Nyl.) Printzen (Ramalinaceae)
Veneto • Belluno, Pordoi, between Rifugio Vièl dal Pan and Rifugio Fredarola, 46°28'35"N, 11°49'29"E, 2492 m, on the basal part of *Salix* sp., 8 August 2024, leg. et det. J. Nascimbene, Hb. Nascimbene (9362).
Emilia-Romagna • Reggio Emilia, Appennino Tosco-Emiliano Ntl Park, below Mt. Casarola Anticima Nord, 44°20'00"N, 10°12'27"E, 1939 m, on terricolous bryophytes, 29 May 2024, leg. J. Nascimbene, C. Pistocchi, L. Di Nuzzo, L. Francesconi & G. Gheza, det. S. Girlanda & C. Pistocchi, Hb. Nascimbene (9743). • Bologna, Corno alle Scale Natural Park, above Passo della Porticciola towards Punta Sofia, 44°07'42"N, 10°49'26"E, 1712 m, on bark, 25 June 2025, leg. C. Pistocchi, L. Francesconi & F. Zinato, det. S. Girlanda & C. Pistocchi, Hb. Nascimbene (10139). • Reggio Emilia, Appennino-Tosco Emiliano Ntl Park, between Rifugio Cesare Battisti and Il Passone, 44°15'54"N, 10°54'58"E, 1803 m, on plant debris, 8 July 2025, leg. C. Pistocchi, S. Girlanda & F. Zinato, det. C. Pistocchi, Hb. Nascimbene (9889). • Reggio Emilia, Appennino Tosco-Emiliano Ntl Park, below Mt. Cusna, 44°17'18"N, 10°23'30"E, 2098 m, on soil, 10 July 2025, leg. C. Pistocchi, S. Girlanda & F. Zinato, det. C. Pistocchi, Hb. Nascimbene (9942). • Reggio Emilia, Appennino Tosco-Emiliano Ntl Park, below Mt. Casarola Anticima Nord, 44°20'11"N, 10°12'36"E, 1776 m, on plant debris, 15 July 2025, leg. C. Pistocchi, S. Girlanda & F. Zinato, det. S. Girlanda & C. Pistocchi, Hb. Nascimbene (10136). • Modena, Alto Appennino Modenese Natural Park, Mt. Cimone (summit area), 44°11'40"N, 10°41'54"E, 2137 m, on soil, 4 August 2025, leg. C. Pistocchi, S. Girlanda & F. Zinato, det. S. Girlanda & C. Pistocchi, Hb. Nascimbene (10094). • Modena, Alto Appennino Modenese Natural Park, below Mt. Cimone, above "Il Casotto" of the Aeronautica Militare, 44°11'46"N, 10°42'17"E, 1914 m, on soil, 5 August 2025, leg. C. Pistocchi, S. Girlanda & F. Zinato, det. C. Pistocchi, Hb. Nascimbene (10114). • Reggio Emilia, Appennino Tosco-Emiliano Ntl Park, between Mt. Cusna and Ricovero Rio Grande, 44°17'47"N, 10°23'51"E, 1756 m, on plant debris, 6 August 2025, leg. C. Pistocchi, S. Girlanda & F. Zinato, det. C. Pistocchi, Hb. Nascimbene (10166).
One of the commonest *Biatora*-species in the Alps, especially on plant remains and on basal parts of subalpine shrubs, overlooked, and certainly more widespread in the Alps, and also known from the Northern Apennines.

Biatorella germanica Körb. (Biatorellaceae)
Piemonte • Cuneo, ridge W above Colle del Vallonetto (Vallone dell'Arma), 44°23'10"N, 7°07'00"E, 2500 m, on calcareous rocks, 23 July 2000, leg. P. L. Nimis & M. Tretiach (with J. Hafellner), det. P. L. Nimis, TSB (34055).
On sheltered calcareous rocks in upland areas; perhaps overlooked and more widespread in the Alps, but certainly not common.

Bilimbia microcarpa (Th. Fr.) Th. Fr. (Ramalinaceae)
Piemonte • Cuneo, Marguareis Natural Park, Cima di Pertegue (Cima di Pertegà) W above Upega just E below the summit, 44°08'50"N, 7°40'50"E, 2360 m, on soil over calcareous substrata, 20 July 2000, leg. P. L. Nimis & M. Tretiach (with J. Hafellner), TSB (33079).

Emilia-Romagna • Reggio Emilia, Appennino Tosco-Emiliano Ntl Park, Mt. Casarola, 44°19’55”N, 10°12’28”E, 1972 m, on soil, 29 May 2024, leg. J. Nascimbene, C. Pistocchi, L. Di Nuzzo, L. Francesconi & G. Gheza, det. S. Girlanda & C. Pistocchi, Hb. Nascimbene (9705). • Reggio Emilia, Appennino Tosco-Emiliano Ntl Park, between Rifugio Cesare Battisti and Il Passone, 44°15’54”N, 10°54’58”E, 1803 m, on soil, 8 July 2025, leg. C. Pistocchi, S. Girlanda & F. Zinato, det. C. Pistocchi, Hb. Nascimbene (9887). • Modena, Alto Appennino Modenese Natural Park, Mt. Cimone (summit area), 44°11’40”N, 10°41’54”E, 2137 m, on soil, 4 August 2025, leg. C. Pistocchi, S. Girlanda & F. Zinato, det. S. Girlanda & C. Pistocchi, Hb. Pistocchi (791). • Reggio Emilia, Appennino Tosco-Emiliano Ntl Park, between Mt. Cusna and Ricovero Rio Grande, 44°17’34”N, 10°23’46”E, 1914 m, on soil, 6 August 2025, leg. C. Pistocchi, S. Girlanda & F. Zinato, det. C. Pistocchi & G. Gheza, Hb. Nascimbene (10160).
Liguria • Imperia, slopes SW above the village Monesi, 44°04’20”N, 7°44’10”E, 1630 m, on calciferous soil, 18 July 2000, leg. P. L. Nimis & M. Tretiach (with J. Hafellner), det. P. L. Nimis, TSB (33043).
An arctic-alpine lichen found on mosses in dry grasslands of upland areas, sometimes on epilithic bryophytes, with optimum near or above tree line.

Blastenia ammiospila (Ach.) Arup, Søchting & Frödén (Teloschistaceae)
Piemonte • Torino, Conca Cialancia Natural Park, mountains W of Pinerolo, NE slopes and ridges of the Punta Cialáncia S above the village Perrero, 44°53’00”N, 7°07’20”E, 2350 m, on plant remnants, 26 July 2001, leg. J. Hafellner (with P. L. Nimis & M. Tretiach), det. J. Hafellner, GZU (JH69428). • Torino, Gran Bosco di Salbertrand Natural Park, mountain ridge S of the Valle di Susa, SW above the Colle dell'Assietta, 45°03’55”N, 6°57’10”E, 2550 m, on plant remnants, 27 July 2001, leg. J. Hafellner (with P. L. Nimis & M. Tretiach), det. J. Hafellner, GZU (JH75575). • Torino, mountains NE of Bardonecchia, western slopes of Punta Sommeiller, S below Colle de Sommeiller, 45°07’35”N, 6°50’15”E, 2850 m, on plant remnants, 28 July 2001, leg. J. Hafellner (with P. L. Nimis & M. Tretiach), det. J. Hafellner, GZU (JH87432). • Cuneo, Monviso Natural Park, N slopes of Monviso, W above Crissolo just W above Lago Fiorenza (S above Pian del Re), 44°41’50”N, 7°05’35”E, 2130 m, on soil over ophiolitic rocks, 25 July 2021, leg. P. L. Nimis & M. Tretiach (with J. Hafellner), det. P. L. Nimis, TSB (35220).
A mainly arctic-alpine to boreal-montane, bipolar lichen found on terricolous mosses and plant debris, more rarely on decaying, rather soft lignum or on small shrubs, most frequent above or near tree line (Alps, Northern and Central Apennines).

Blastenia hungarica (H. Magn.) Arup, Søchting & Frödén (Teloschistaceae)
Friuli Venezia Giulia • Pordenone, Loc. Claut, 46°16’07”N, 12°30’57”E, 1650 m, on bark, 15 May 1983, leg. E. Loi, det. P. L. Nimis, TSB (3596).
Liguria • Imperia, slopes SW above the village Monesi, 44°04’20”N, 7°44’10”E, 1630 m, on bark of *Larix*, 18 July 2000, leg. P. L. Nimis & M. Tretiach (with J. Hafellner), det. P. L. Nimis, TSB (33061).
A temperate to boreal-montane lichen found on twigs of acid-barked trees.

Botryolepraria lesdainii (Hue) Canals, Hern.-Mar., Gómez-Bolea & Llimona (*incertae sedis*)
Piemonte • Vercelli, Grotte di Ara, 45°41’50”N, 8°19’34”E, 419 m, on a slight soil layer above calcareous rocks, 2 March 2025, leg. et det. G. Gheza, Hb. Gheza (25030201).
On limestone, calcareous sandstone, basic siliceous rocks (e.g., basalt, lava), epilithic mosses and soil, sometimes on walls, especially in fissures, in shaded sites protected from rain, certainly much more common throughout Italy, below the subalpine belt. According to Baruffo et al. (2006) this is the most shade-loving among all leprarioid species.

Bryobilimbia fissuriseda (Poelt) Timdal, Marthinsen & Rui (Lecideaceae)
Piemonte • Cuneo, ridge W above Colle del Vallonetto (Vallone dell’Arma), 44°23’10”N, 7°07’00”E, 2500 m, on calcareous rocks, 18 July 2000, leg. J. Hafellner (with P. L. Nimis & M. Tretiach), det. J. Hafellner, GZU (JH87576). • Cuneo, *ibidem*, 23 July 2000, leg. P. L. Nimis & M. Tretiach (with J. Hafellner), det. P. L. Nimis, TSB (34028). • Cuneo, Marguareis Natural Park, Cima di Pertegue (Cima di Pertegà), W above Upega just E

below the summit, 44°08’50’’N, 7°40’50’’E, 2360 m, on outcrops of calcareous schists, 20 July 2000, leg. J. Hafellner (with P. L. Nimis & M. Tretiach), det. J. Hafellner, GZU (JH59409). • Cuneo, on the pass between Cima di Pertegue (Cima di Pertegà) and Rocca del Fera, W above Upega, 44°08’50’’N, 7°41’40’’E, 2180 m, on outcrops of calcareous schists, 20 July 2000, leg. J. Hafellner (with P. L. Nimis & M. Tretiach), det. J. Hafellner, GZU (JH87616).
A probably circumpolar, arctic-alpine lichen found in thin fissures of calciferous rocks (calcareous schist, dolomite, much more rarely pure limestone) near and especially above tree line; certainly more widespread in the Alps.

Bryoplaca sinapisperma (DC.) Søchting, Frödén & Arup (Teloschistaceae)
Emilia-Romagna • Reggio Emilia, Appennino Tosco-Emiliano Ntl Park, below Mt. Cusna, 44°17’19’’N, 10°23’27’’E, 2075 m, on terricolous bryophytes, 10 July 2025, leg. C. Pistocchi, S. Girlanda & F. Zinato, det. C. Pistocchi, Hb. Nascimbene (9933).
A holarctic lichen ranging from the arctic zone to the high southern mountains, found on mosses and plant debris on calcareous or base-rich siliceous substrata, with optimum near or above tree line, sometimes reaching the montane belt in open habitats; common in the Alps, restricted to the highest areas of the Apennines.

Buellia dispersa (A. Massal.) A. Massal. (Caliciaceae)
Piemonte • Cuneo, crest SW above Colle dell’Agnello, 44°40’58’’N, 6°58’39’’E, 2830 m, on calciferous schists, 25 July 2000, leg. P.L. Nimis & M. Tretiach (with J. Hafellner), det. P.L. Nimis, TSB (33125). • Cuneo, Valle Varaita, ca. 4 Km E of Sampeyre W of Villar on S-exposed ophiolitic slopes, 44°34’55’’N, 7°08’05’’E, 1120 m, on siliceous base-rich rocks, 25 July 2000, leg. P. L. Nimis & M. Tretiach (with J. Hafellner), det. P. L. Nimis, TSB (34632).
A xeric subtropical to temperate lichen of base-rich or slightly lime-containing siliceous rocks in warm-dry situations, present both in the Mediterranean area and in dry Alpine valleys.

Buellia jugorum (Arnold) Arnold (Caliciaceae)
Emilia-Romagna • Reggio Emilia, Appennino Tosco-Emiliano Ntl Park, Mt. Prado near Passo di Monte Vecchio, 44°14’39’’N, 10°24’39’’E, 1987 m, on soil, 21 June 2023, leg. et det. L. Di Nuzzo, Hb. Di Nuzzo (101).
An arctic-alpine to boreal-montane, circumpolar species found on small siliceous pebbles in wind-exposed ridges, sometimes overgrowing other crustose lichens, mostly near and above tree line; probably more widespread in the Alps, certainly rarer in the high Mediterranean mountains.

Buellia uberior Anzi (Caliciaceae)
Piemonte • Cuneo, Colle di Sampeyre, on the ridge just W of the pass, 44°33’10’’N, 7°06’55’’E, 2300 m, on ophiolitic rocks, 26 July 2000, leg. J. Hafellner (with P. L. Nimis & M. Tretiach), det. J. Hafellner, GZU (JH87286). • Torino, Conca Cialancia Natural Park, mountains W of Pinerolo, northeastern slopes and ridges of Punta Cialáncia S above Perrero, 44°53’00’’N, 7°07’20’’E, 2350 m, on siliceous rocks, 26 July 2001, leg. J. Hafellner (with P. L. Nimis & M. Tretiach), det. J. Hafellner, GZU (JH69362).
Buellia uberior is a poorly competitive species with a tendency to lichenicolous growth, frequently on *Schaereria fuscocinerea* (Nyl.) Clauzade & Cl.Roux. Both specimens reported here show autotrophic thalli but have the diagnostic striate ascospores (Scheidegger 1987). The species is widely distributed in the Holarctic region. In Italy, it has relatively few records, mainly from the Alps – including the type locality in Lombardia – but it has also been recorded from the Northern Apennines and Sardinia.

Calicium notarisii (Tul.) M. Prieto & Wedin (Caliciaceae)
Piemonte • Cuneo, Colle di Sampeyre just W of the Pass, 44°33’10’’N, 7°06’55’’E, 2300 m, on wood of conifers, 26 July 2000, leg. P. L. Nimis & M. Tretiach (with J. Hafellner), det. P. L. Nimis, TSB (32805).

A mainly cool-temperate to southern boreal-montane lichen found on dry, weathered wood (e.g., on fences, wooden poles), but also on bark of old acid-barked trees (especially *Quercus*). Widespread, but rare, from the Alps to the mountains of Sardinia.

Calogaya oblitterata (Pers.) P.F. Cannon & Coppins (Teloschistaceae)
Veneto • Belluno, Marmolada, Val de Ombreta, 46°25'31"N, 11°52'04"E, 2010 m, on volcanic rocks, 21 August 2025, leg. et det. J. Nascimbene, Hb. Nascimbene (10017).
Piemonte • Cuneo, crest SW above Colle dell'Agnello, 44°40'58"N, 6°58'39"E, 2830 m, on calciferous schists, 25 July 2000, leg. P. L. Nimis & M. Tretiach (with J. Hafellner), det. P.L. Nimis, TSB (33185).
A very polymorphic taxon with a mainly temperate to boreal distribution in Europe, also known from the southern European mountains, most frequent on base-rich siliceous rocks or on decalcified calcareous rocks, usually in nutrient-poor stands, both on vertical walls of cliffs and overhangs and on horizontal surfaces of siliceous boulders.

Caloplaca isidiigera Vězda (Teloschistaceae)
Friuli Venezia Giulia • Udine, Natura 2000 Site-Creta di Aip e Sella di Lanza, Passo Cason di Lanza, 46°33'7"N, 13°11'27"E, 1350 m, on calciferous siliceous rocks, 28 May 1990, leg. F. Bersan, det. P. L. Nimis, TSB (14195).
Veneto • Belluno, Tre Cime di Lavaredo, near Rifugio Auronzo, 46°36'47"N, 12°17'46"E, 2300 m, on dolomitic rocks, 31 August 1994, leg. et det. P. L. Nimis, TSB (19719).
A calcicolous species of nutrient-enriched rocks in upland areas, in the past often confused with similar species.

Caloplaca stillicidiorum (Vahl) Lynge **var. *muscorum*** (A. Massal.) (Teloschistaceae)
Liguria • Imperia, Alpi Liguri Natural Park, mountains N above Triora on the road to Col Garezzo, 44°02'50"N, 7°45'15"E, 1650 m, on calciferous soil and plant debris, 21 July 2000, leg. P. L. Nimis & M. Tretiach (with J. Hafellner), det. P. L. Nimis, TSB (33470).
Here we place populations growing on mosses and plant debris, below the subalpine belt and mostly in dry-continental areas, on basic siliceous substrata, with intensely orange apothecial discs. They represent a heterogeneous assemblage of ecotypes whose taxonomy has not been clarified yet.

Caloplaca conciliascens (Nyl.) Zahlbr. (Teloschistaceae)
Piemonte • Cuneo, Marguareis Natural Park, just NW above Carnino, 44°08'56"N, 7°43'44"E, 1400 m, on slightly calciferous blue-green schists, 19 July 2000, leg. P. L. Nimis & M. Tretiach (with J. Hafellner), det. P. L. Nimis, TSB (33345).
A poorly known species, perhaps a synonym of *Lendemeriella exsecuta*, known from the locus classicus in Tyrol, Poland, a single locality in the French Maritime Alps, and from dry-warm sites in central Sardinia. This is the first record for the Italian Alps.

Caloplaca interna Poelt & Nimis (Teloschistaceae)
Piemonte • Cuneo, Marguareis Natural Park, just NW above the village Carnino, 44°08'56"N, 7°43'44"E, 1400 m, on slightly calciferous blue-green schists, 19 July 2000, leg. P. L. Nimis & M. Tretiach (with J. Hafellner), det. P. L. Nimis, TSB (33286).
On south-facing, vertical surfaces of basic siliceous rocks which are, albeit seldom, wetted by water seepage after rain, often found near *Peltula*-stands (the host being mostly sterile), but somehow less bound to periodical seepage of water; fairly common above the south-facing doors of Sardinian basaltic Nuraghes, and also found in dry-continental Alpine valleys. It does not belong to *Caloplaca* s. str.

Caloplaca livida (Hepp) Jatta (Teloschistaceae)
Emilia-Romagna • Modena, Alto Appennino Modenese Natural Park, Mt. Cimone, near "Il Casotto" of the Aeronautica Militare, 44°11'52"N, 10°42'15"E, 1880 m, on plant debris, 21 October 2022, leg. L. Francesconi, det. J. Nascimbene, Hb. Nascimbene (8808).

An arctic-alpine species found on plant debris and bryophytes overgrowing acid siliceous rocks, with optimum near and above treeline. The species belongs to *Bryoplaca* (Arup in litt.).

Candelariella commutata Otte & M. Westb. (Candelariaceae)
Piemonte • Cuneo, ridge SE above Colle Valcavera-Vallone dell'Arma, 44°22'40"N, 7°06'15"E, 2440 m, on soil over calciferous schists, 23 July 2000, leg. P. L. Nimis & M. Tretiach (with J. Hafellner), det. P. L. Nimis, TSB (34530).
An arctic-alpine species, widespread in the mountains of the southern holarctic zone, on epilithic mosses on limestone and dolomite, a characteristic element of calcareous mountains, with optimum above tree line.

Candelariella kuusamoënsis Räsänen (Candelariaceae)
Emilia-Romagna • Bologna, Corno alle Scale Natural Park, below Punta Sofia, 44°07'27"N, 10°49'39"E, 1850 m, on terricolous bryophytes over calcareous soil, 22 October 2021, leg. et det. J. Nascimbene, Hb. Nascimbene (8033).
Liguria • Imperia, Alpi Liguri Natural Park, just E below Upega on right bank of the creek Negrone (Corvo), 44°07'35"N, 7°44'10"E, 1260 m, on wood, 19 July 2000, leg. P. L. Nimis & M. Tretiach (with J. Hafellner), det. P. L. Nimis, TSB (33559).
A boreal-montane, poorly understood lichen found on the top of poles and wooden fences, on plant debris and soil, more rarely on rocks in upland areas; certainly more widespread in the Alps. The delimitation of this species is problematic: most of the material distributed in exsiccata belongs to other species, and it is doubtful whether the material called "*C. kuusamoensis*" by Central and Southern European authors really corresponds to the type material, which in itself resembles a luxuriant *C. vitellina* growing on mosses (Westberg, in litt.). The record from Emilia-Romagna is the first one for the Apennines.

Carbonea atronivea (Arnold) Hertel (Lecanoraceae)
Piemonte • Cuneo, crest SW above Colle dell'Agnello, 44°40'58"N, 6°58'39"E, 2830 m, on calciferous schists, 25 July 2000, leg. P.L. Nimis & M. Tretiach (with J. Hafellner), det. P.L. Nimis, TSB (33159).
An arctic-alpine, circumpolar species found on lime-containing siliceous rocks (e.g. calciferous schists) near or above tree line, starting the life-cycle on species of *Lecidella*; certainly overlooked and more widespread in the Alps.

Carbonea distans (Kremp.) Hafellner & Obermayer (Lecanoraceae)
Piemonte • Cuneo, Rocca dell'Abisso W of Colle di Tenda, E below summit, steep slopes towards uppermost Vallone dell'Abisso, 44°08'37"N, 7°30'29"E, 2630 m, on steep rock faces of large boulders (gneiss), 22 July 2000, leg. J. Hafellner (with P. L. Nimis & M. Tretiach), det. J. Hafellner, GZU (JH87368). • Cuneo, cirque E below the summit of Rocca dell'Abisso (Vallone dell'Abisso), W of Colle di Tenda, NW below the Fort de Giaure, 44°08'25"N, 7°31'05"E, 2250 m, on steep rock faces of large boulders (gneiss), 22 July 2000, leg. J. Hafellner (with P. L. Nimis & M. Tretiach), det. J. Hafellner, GZU (JH87589). • Cuneo, Colle della Lombarda, slopes exposed to the W just NE below the pass, 44°12'20"N, 7°09'10"E, 2360 m, on gneiss, 24 July 2000, leg. J. Hafellner (with P. L. Nimis & M. Tretiach), det. J. Hafellner, GZU (JH87688). • Cuneo, Monviso Natural Park, N slopes of Monviso, W above Crissolo just W above Lago Fiorenza (S above Pian del Re), 44°41'50"N, 7°05'35"E, 2130 m, on ophiolitic rocks, 25 July 2021, leg. P. L. Nimis & M. Tretiach (with J. Hafellner), det. P. L. Nimis, TSB (35201).
Valle d'Aosta • Aosta, Colle del Gran San Bernardo, SW below the pass, 45°52'18"N, 7°09'34"E, 2500 m, on vertical siliceous rock faces, 1 August 2001, leg. J. Hafellner (with P. L. Nimis & M. Tretiach), det. J. Hafellner, GZU (JH87137).
An arctic-alpine obligate parasite on the thalli of *Orphniospora mosigii*, but as the thalli may become rather large the host may not be present in some specimens. The species is found on slightly to steeply inclined, exposed faces of hard siliceous rocks near or above tree line. As the host species is fairly common in the alpine belt of the siliceous Alps, this lichenicolous species might be also more widespread.

Carbonicola anthracophila (Nyl.) Bendiksby & Timdal (Carbonicolaceae)
Piemonte • Cuneo, Vallone della Stura di Demonte 1 Km E of Festiona, 44°18’05”N, 7°21’30”E, 720 m, on bark of *Castanea* sp., 24 July 2000, leg. P. L. Nimis & M. Tretiach (with J. Hafellner), det. P. L. Nimis, TSB (33499).
A circumboreal-montane lichen found on charred wood in upland areas. Our record is the first from the Italian Alps.

Catapyrenium daedaleum (Kremp.) Stein (Verrucariaceae)
Piemonte • Cuneo, western ridge of Mt. Nebin ca. 1 Km E of Colle di Sampeyre, 44°32’40”N, 7°08’20”E, 2380 m, on soil, 26 July 2000, leg. P. L. Nimis & M. Tretiach (with J. Hafellner), det. P. L. Nimis, TSB (32912).
A boreal-montane to arctic-alpine, circumpolar species found on plant debris, mosses and bare, humus-rich soil on calciferous ground near or above tree line.

Catillaria picila (A. Massal.) Coppins (Catillariaceae)
Piemonte • Cuneo, ridge W above Colle del Vallonetto (Vallone dell’Arma), 44°23’10”N, 7°07’00”E, 2500 m, on calcareous rocks, 23 July 2000, leg. P. L. Nimis & M. Tretiach (with J. Hafellner), det. P. L. Nimis, TSB (34062).
A mild-temperate species found on steeply inclined to rain-sheltered surfaces of compact calcareous rocks, especially limestone but also calciferous schists, often together with *C. minuta*. It does not belong to *Catillaria* s. str.

Cetraria obtusata (Schaer.) van den Boom & Sipman (Parmeliaceae)
Veneto • Belluno, Pordoi, between Rifugio Vièl dal Pan and Rifugio Fredarola, 46°28’35”N, 11°49’29”E, 2492 m, on organic soil, 8 August 2024, leg. et det. J. Nascimbene, Hb. Nascimbene (9351).
Piemonte • Cuneo, Rocca dell’Abisso W of Col di Tenda just N below the summit, 44°08’35”N, 7°30’15”E, 2750 m, on soil over gneiss, 22 July 2000, leg. P. L. Nimis & M. Tretiach (with J. Hafellner), det. P. L. Nimis, TSB (34556).
Ecologically similar to *C. ericetorum*, but much rarer, and perhaps more bound to dry-continental situations above tree line; probably confined to the Alps in Italy.

Chaenotheca gracilenta (Ach.) Mattsson & Middelb. (Coniocybaceae)
Piemonte • Cuneo, Vallone della Stura di Demonte 1 Km E of Festiona, 44°18’05”N, 7°21’30”E, 720 m, on bark of *Castanea* sp., 24 July 2000, leg. P. L. Nimis & M. Tretiach (with J. Hafellner), det. P. L. Nimis, TSB (33502).
A circumboreal-montane species found on rotting wood and decaying bark, on faces protected from rain such as hollows of old stumps in ancient, humid, montane to subalpine forests, reported from the Alps and the Northern Apennines.

Chaenotheca stemonea (Ach.) Müll. Arg. (Coniocybaceae)
Liguria • Imperia, Alpi Liguri Natural Park, just E below Upega on right bank of the creek Negrone (Corvo), 44°07’35”N, 7°44’10”E, 1260 m, on bark of *Larix*, 19 July 2000, leg. P. L. Nimis & M. Tretiach (with J. Hafellner), det. P. L. Nimis, TSB (33550).
A cool-temperate to boreal-montane, circumpolar lichen found in rain-protected hollows of conifer trunks inside forests, especially near the ground, both on bark and lignum, sometimes on acid-barked deciduous trees, e.g. *Betula* sp. and *Quercus* sp. Widespread in Italy, but not common.

Cladonia acuminata (Ach.) Norrl. (Cladoniaceae)
Veneto • Belluno, Dolomiti Bellunesi Ntl Park, Pezedei, below Forcella del Zità Sud, 46°16’27”N, 12°09’13”E, 2285 m, on soil, 2 July 2024, leg. J. Nascimbene, det. G. Gheza, Hb. Nascimbene (9948).
A cool-temperate to circumboreal-montane lichen found on calciferous soil rich in humus in open situations, most frequent in the mountains.

Cladonia arbuscula (Wallr.) Flot. (Cladoniaceae)
Emilia-Romagna • Bologna, Corno alle Scale Natural Park, below Punta Sofia, 10°49'24"N, 44°07'43"E, 1680 m, on siliceous soil, 22 October 2021, leg. et det. G. Gheza & C. Pistocchi, Hb. Gheza (21102216). • Reggio Emilia, Ventasso, trail between Mt. Piano and Buca del Moro, 44°20'57"N, 10°13'19"E, 1450 m, on organic soil, 29 May 2024, leg. L. Di Nuzzo, det. G. Gheza, Hb. Nascimbene (9413). • Bologna, Corno alle Scale, between Passo della Porticciola and Punta Sofia, 44°07'34"N, 10°49'31"E, 1762 m, on soil, 25 June 2025, leg. C. Pistocchi, L. Francesconi & F. Zinato, det. C. Pistocchi & S. Girlanda, Hb. Nascimbene (10144). • Reggio Emilia, Appennino Tosco-Emiliano Ntl Park, below Sella del Casarola, 44°19'52"N, 10°12'17"E, 1936 m, on soil, 14 July 2025, leg. C. Pistocchi, S. Girlanda & F. Zinato, det. C. Pistocchi & S. Girlanda, Hb. Nascimbene (10132). • Modena, Alto Appennino Modenese Natural Park, Mt. Cimone, below Rifugio Pian Cavallaro, 44°12'18"N, 10°42'23"E, 1753 m, on soil, 5 August 2025, leg. C. Pistocchi, S. Girlanda & F. Zinato, det. C. Pistocchi & S. Girlanda, Hb. Nascimbene (10118).
A circumpolar, boreal-subarctic-subalpine lichen, one of the most abundant elements of lichen-rich tundra-like vegetation on mineral soil in exposed habitats, sometimes also found on lignum.

Cladonia botrytes (K.G. Hagen) Willd. (Cladoniaceae)
Lombardia • Brescia, Valle Arcina below Casinone di Arcina, 45°53'17"N, 10°22'41"E, 1729 m, on a rotting larch stump, 30 August 2019, leg. et det. G. Gheza, Hb. Gheza.
A rare but widespread species in the Alps (Nimis et al. 2018), bound to rotting wood. All the previous records from Lombardia are uncorrect (see Ravera et al. 2019 for details).

Cladonia cervicornis (Ach.) Flot. **(**Cladoniaceae)
Emilia-Romagna • Bologna, Corno alle Scale Natural Park, between Passo della Porticciola and Punta Sofia, 44°07'29"N, 10°49'37"E, 1815 m, on soil, 26 June 2025, leg. C. Pistocchi, L. Francesconi & F. Zinato, det. G. Gheza & C. Pistocchi, Hb. Nascimbene (10148). • Reggio Emilia, Appennino Tosco-Emiliano Ntl Park, Mt. Cusna, below Cima dell'Angelo, 44°16'22"N, 10°25'07"E, 1935 m, on soil, 9 July 2025, leg. C. Pistocchi, S. Girlanda & F. Zinato, det. G. Gheza & C. Pistocchi, Hb. Nascimbene (9899).
A temperate to southern boreal-montane lichen found on mineral siliceous soil in open grasslands and garrigues.

Cladonia cryptochlorophaea Asahina (Cladoniaceae)
Trentino-Alto Adige • Trento, Adamello-Brenta Natural Park, Valle delle Seghe, above Rifugio Croz dell'Altissimo, 46°10'19"N, 10°55'49"E, 1467 m, on soil, 24 June 2022, leg. L. Francesconi, det. G. Gheza, Hb. Nascimbene (8959, 9414), Hb. Francesconi (68).
A mainly cool-temperate, perhaps holarctic lichen found on soil rich in humus, on peat, etc., probably with a western distribution in Europe.

Cladonia decorticata (Flörke) Spreng. (Cladoniaceae)
Emilia-Romagna • Reggio Emilia, Appennino-Tosco Emiliano Ntl Park, below Mt. Casarola, 44°20'10"N, 10°12'33"E, 1811 m, on organic soil, 29 May 2024, leg. et det. G. Gheza, Hb. Gheza (24052908). • Bologna, Strada delle Malghe below Corno alle Scale, 44°07'29"N, 10°48'59"E, 1590 m, on organic soil, 29 October 2024, leg. et det. G. Gheza, Hb. Gheza (24102919). • Bologna, Corno alle Scale Natural Park, below Punta Sofia, 44°07'28"N, 10°49'39"E, 1834 m, on plant debris, 26 June 2025, leg. C. Pistocchi, L. Francesconi & F. Zinato, det. G. Gheza & C. Pistocchi, Hb. Pistocchi (817). • Reggio Emilia, Appennino Tosco-Emiliano Ntl Park, near Passo di Lama Lite, 44°15'36"N, 10°24'50"E, 1777 m, on soil, 8 July 2025, leg. C. Pistocchi, S. Girlanda & F. Zinato, det. G. Gheza & C. Pistocchi, Hb. Nascimbene (9885). • Reggio Emilia, Appennino Tosco-Emiliano Ntl Park, below Alpe di Succiso, 44°19'59"N, 10°11'49"E, 1994 m, on soil, 14 July 2025, leg. C. Pistocchi, S. Girlanda & F. Zinato, det. G. Gheza & C. Pistocchi, Hb. Nascimbene (10128). • Modena, Alto Appennino Modenese Natural Park, below Mt. Cimone, near "Il Casotto" of the Aeronautica Militare,

44°11’52”N, 10°42’15”E, 1876 m, on soil, 5 August 2025, leg. C. Pistocchi, S. Girlanda & F. Zinato, det. G. Gheza & C. Pistocchi, Hb. Nascimbene (10167).
A typical species of high-altitude open habitats on siliceous substrates, rare but quite widespread in the Alps (Nimis et al. 2018, Nimis 2026), but never reported before from the Apennines, where it is likely limited to the highest siliceous mountains of the northernmost stretch.

Cladonia diversa S. Stenroos (Cladoniaceae)
Veneto • Belluno, Casera Razzo 1 Km from the border between Friuli and Veneto, 46°28’36”N, 12°37’14”E, 1800 m, on soil over siliceous substrata, 1986, leg. et det. P. L. Nimis, TSB (7825).
Related to *C. coccifera* and *C. pleurota*, and with a similar ecology, but perhaps more bound to humid habitats, probably more widespread in the Alps near or above tree line.

Cladonia macrophyllodes Nyl. (Cladoniaceae)
Emilia-Romagna • Bologna, Corno alle Scale Natural Park, below Punta Sofia, 44°07’30”N, 10°49’34”E, 1790 m, on siliceous soil, 22 October 2021, leg. et det. G. Gheza, Hb. Gheza (21102227). • Modena, Alto Appennino Modenese Natural Park, below Mt. Cimone, 44°11’52”N, 10°42’15”E, 1880 m, on organic soil, 21 October 2022, leg. et det. G. Gheza, Hb. Gheza (22102114), Hb. Pistocchi (179). • Reggio Emilia, Appennino Tosco-Emiliano Ntl Park, below Mt. Casarola Anticima Nord, 44°20’08”N, 10°12’26”E, 1850 m, on soil, 29 May 2024, leg. J. Nascimbene, C. Pistocchi, L. Di Nuzzo, L. Francesconi & G. Gheza, det. C. Pistocchi & G. Gheza, Hb. Nascimbene (9808). • Reggio Emilia, Appennino Tosco- Emiliano Ntl Park, Sella di Casarola, 44°19’59”N, 10°12’27”E, 1939 m, on siliceous soil, 29 May 2024, leg. et det. G. Gheza, Hb. Gheza (24052903). • Reggio Emilia, Appennino Tosco-Emiliano Ntl Park, Alpe di Succiso, 44°19’59”N, 10°11’47”E, 2010 m, on soil, 29 May 2024, leg. J. Nascimbene, C. Pistocchi, L. Di Nuzzo, L. Francesconi & G. Gheza, det. G. Gheza & C. Pistocchi, Hb. Nascimbene (9823). • Reggio Emilia, Appennino Tosco-Emiliano Ntl Park, below Mt. Cusna, 44°17’24”N, 10°23’32”E, 2018 m, on soil, 10 July 2025, leg. C. Pistocchi, S. Girlanda & F. Zinato, det. G. Gheza & C. Pistocchi, Hb. Nascimbene (9919). • Reggio Emilia, Appennino Tosco-Emiliano Ntl Park, below Mt. Cusna, 44°17’19”N, 10°23’27”E, 2075 m, on soil, 10 July 2025, leg. C. Pistocchi, S. Girlanda & F. Zinato, det. G. Gheza & C. Pistocchi, Hb. Nascimbene (9938). • Reggio Emilia, Appennino Tosco-Emiliano Ntl Park, below Sella del Casarola towards Rifugio Rio Pascolo, 44°19’59”N, 10°12’18”E, 1835 m, on soil, 14 July 2025, leg. C. Pistocchi, S. Girlanda & F. Zinato, det. C. Pistocchi & G. Gheza, Hb. Nascimbene (10134). • Modena, Alto Appennino Modenese Natural Park, Corno alle Scale, near Rifugio Malghe, 44°07’17”N, 10°48’45”E, 1661 m, on soil, 29 October 2025, leg. C. Pistocchi, det. G. Gheza & C. Pistocchi, Hb. Pistocchi (809).
Toscana • Lucca, Mt. Prado near Passo di Monte Vecchio, 44°14’30”N, 10°24’40”E, 1927 m, on siliceous soil, 21 June 2023, leg. L. Di Nuzzo, det. G. Gheza, Hb. Di Nuzzo (99), Hb. Gheza (23111902).
Cladonia macrophyllodes has been recorded mainly from the Alps in Italy, with a couple of records from Sardegna and Calabria (Nimis 2026). These are the first records from the Northern Apennines, where the species is widespread in open habitats at the highest elevations.

Cladonia novochlorophaea (Sipman) Brodo & Ahti (Cladoniaceae)
Lombardia • Bergamo, Orobie Bergamasche Natural Park, Passo Branchino, 45°56’54”N, 9°48’03”E, 1820 m, on calcareous soil, 2 May 2025, leg. et det. G. Gheza, Hb. Gheza (25050206).
On humus, decaying wood, mossy rocks and, occasionally, on bare soil, usually mixed with other morphologically similar taxa and hence often overlooked.

Cladonia phyllophora Hoffm. (Cladoniaceae)
Friuli Venezia Giulia • Udine, Natura 2000 Site-Creta di Aip e Sella di Lanza, Passo Cason di Lanza, 46°33’22”N, 13°10’06”E, 1800 m, on soil over siliceous substrata, 25 May 1995, leg. F. Bersan & A. Rondi, det. P. L. Nimis, TSB (20592).
A cool-temperate to boreal-montane, probably holarctic lichen found on acid mineral soil.

Cladonia straminea (Sommerf.) Flörke (Cladoniaceae)
Lombardia • Brescia, main trail of the Val di Campovecchio, 46°08'29"N, 10°11'22"E, 1358 m, on a mica-schist outcrop, 19 August 2021, leg. et det. G. Gheza, Hb. Gheza (21081909). • Brescia, Adamello Natural Park, Val Sozzine, 46°14'36"N, 10°32'19"E, 1410 m, on a mossy granite boulder, 10 August 2022, leg. G. Gheza & C. Pistocchi, det. G. Gheza, Hb. Gheza (22081019). • Sondrio, Orobie Valtellinesi Natural Park, trail along Lago Belviso, 46°05'08"N, 10°07'59"E, 1490 m, on a mica-schist outcrop, 22 August 2023, leg. et det. G. Gheza, Hb. Gheza (23082206). • Brescia, Adamello Natural Park, trail between Fabrezza and Passo di Blisiè, 46°05'26"N, 10°26'10"E, 1500 m, on a schist outcrop, 4 August 2025, leg. et det. G. Gheza, Hb. Gheza (25080424). • Brescia, Stelvio Ntl Park, near the Lakes of Ercavallo, 46°19'51"N, 10°32'22"E, 2664 m, 31 July 2025, leg. C. Pistocchi, det. G. Gheza, Hb. Pistocchi (901).
A rare species growing on mosses or on soil in crevices of siliceous rock in rather oceanic areas. It was reported previously in Italy only from Trentino Alto Adige (Nimis 2026).

Cladonia strepsilis (Ach.) Grognot (Cladoniaceae)
Emilia-Romagna • Reggio Emilia, Appennino Tosco-Emiliano Ntl Park, Sella del Monte Prado, 44°15'11"N, 10°23'59"E, 1893 m, on soil, 12 July 2025, leg. C. Pistocchi, S. Girlanda & F. Zinato, det. G. Gheza & C. Pistocchi, Hb. Nascimbene (10121).
Toscana • Lucca, Mt. Prado near Passo di Monte Vecchio, 44°14'30"N, 10°24'40"E, 1927 m, on siliceous soil, 21 June 2023, leg. L. Di Nuzzo, det. G. Gheza, Hb. Di Nuzzo (100), Hb. Gheza (23111901).
A boreal-montane to tropical lichen found on humus-rich soil overlaying siliceous rocks and amongst bryophytes in humid depressions periodically filled by water, in open situations. It was never found in the Apennines before, having been reported mostly from the Alps (Nimis et al. 2018, Nimis 2026) and also from northern lowlands where suitable habitats still occur (Gheza 2018, 2020).

Cladonia trassii Ahti (Cladoniaceae)
Trentino-Alto Adige • Trento, Adamello-Brenta Natural Park, Val Nambrone, near Lago di Cornisello Inferiore, 46°13'19"N, 10°44'16"E, 2100 m, on soil above granite, 28 July 2023, leg. G. Gheza, L. Francesconi & C. Pistocchi, det. G. Gheza, Hb. Gheza (23072801), Hb. Nascimbene (9090). • Trento, Adamello-Brenta Natural Park, Val Nambrone, above Lago di Cornisello Superiore, 46°13'5"N, 10°43'34"E, 2190 m, on terricolous bryophytes, 28 July 2023, leg. G. Gheza, L. Francesconi & C. Pistocchi, det. G. Gheza, Hb. Gheza (23072802), Hb. Nascimbene (9094). • Trento, Adamello-Brenta Natural Park, below Lago delle Malghette, 46°15'49"N, 10°49'26"E, 1810 m, on soil over mossy granite rocks, 8 August 2023, leg. G. Gheza & C. Pistocchi, det. G. Gheza, Hb. Gheza (23080834). • Trento, Val di Sole, between Baito Cadinel and Cima Vegaia, 46°21'14"N, 10°44'13"E, 2584 m, on organic soil, 20 August 2023, leg. C. Pistocchi, det. C. Pistocchi & G. Gheza, Hb. Pistocchi (126). • Trento, Lago di Stablo, 46°16'56"N, 10°46'34"E, 1730 m, on soil, 31 July 2025, leg. et det. G. Gheza, Hb. Gheza (25073103). • Trento, Stelvio Ntl Park, Val del Monte, boulder/pic-nic park loc. Pradacci, 46°20'35"N, 10°37'42"E, 1646 m, on soil over rocks, 23 August 2025, leg. C. Pistocchi, det. G. Gheza & C. Pistocchi, Hb. Nascimbene (10209).
On acid soil near or above tree line; all earlier Italian records of this species are under *C. stricta* or *C. lepidota*. *C. stricta* is an arctic species, and according to Ahti (Nimis 2016) Italian records most likely refer to *C. trassii* (Ahti 1998).

Cladonia uncialis (L.) F.H. Wigg. **subsp. *biuncialis*** (Hoffm.) M. Choisy (Cladoniaceae)
Trentino-Alto Adige • Bolzano, Stelvio Ntl Park, Val Martello below Lyfiknott-Dosso di Livi, 46°30'50"N, 10°41'36"E, 2530 m, on soil, 4 October 2019, leg. et det. J. Nascimbene & C. Vallese, Hb. Nascimbene (7357). • Bolzano, Stelvio Ntl Park, Val Martello between Lyfialm-Malga Lifi and Lyfiknott-Dosso di Livi, 46°30'41"N, 10°41'52"E, 2398 m, on soil, 4 October 2019, leg. et det. J. Nascimbene & C. Vallese, Hb. Nascimbene (7360). • Bolzano, Stelvio Ntl Park, Val Martello below Lyfiknott-Dosso di Livi, 46°31'03"N, 10°41'27"E, 2692 m, on soil, 4 October 2019, leg. et det. J. Nascimbene & C. Vallese, Hb. Nascimbene (7405). • Bolzano, Maddalene Group, Val di Bresimo, Passo Alplaner (Alplahnerschafte), 46°26'48"N, 10°52'36"E, 2430 m, 29 September 2024, leg. C. Pistocchi, det. C. Pistocchi & G. Gheza, Hb. Pistocchi (334).

This subspecies differs from subsp. *uncialis* in the frequent presence of squamatic acid, and in having a powdery inner surface of the podetia. Mixed population may occur, where individuals may be difficult to assign to either subspecies.

Diploschistes candidissimus (Kremp.) Zahlbr. (Graphidaceae)
Piemonte • Cuneo, ridge SE above Colle Valcavera-Vallone dell'Arma, 44°22'40''N, 7°06'15''E, 2440 m, on calciferous schists, 23 July 2000, leg. P. L. Nimis & M. Tretiach (with J. Hafellner), det. P. L. Nimis, TSB (33971).
A mainly Mediterranean species growing on compact calciferous rocks, also found in dry Alpine valleys.

Eiglera flavida (Hepp) Hafellner (Eigleraceae)
Friuli Venezia Giulia • Udine, Mt. Novarza, 46°29'32''N, 12°45'09''E, 1500 m, on siliceous base-rich rocks, 1989, leg. P. L. Nimis, det. M. Tretiach, TSB (14176).• Udine, Natura 2000 Site-Mt. Auernig and Mt. Corona, area of Passo Pramollo (Naßfeldpaß) ca. 6 km N of Pontebba, Mt. Carnizza (Garnitzenberg) E above the pass, small limestone cliffs on the ridge W of the summit, 46°33'35''N, 13°17'36''E, 1840 m, on Palaeozoic limestone, 6 August 2007, leg. et det. J. Hafellner, GZU (JH76190).
An inconspicuous crustose lichen with a thin, greyish to ochre-brownish thallus and aspicilioid apothecia with blackish discs. The blue-green, N+ purple epihymenium and an unstructured euamyloid ascus tholus, both visible in light microscope preparations, are diagnostic for *Eiglera* species among the wide range of aspicilioid lichens. The species is found on various types of limestone and marble, weakly calciferous schists, as well as base-rich igneous rocks (e.g., serpentinite, basalt), often on low outcrops, pebbles and small stones near the ground and therefore under the long-term influence of soil moisture. The species has an overall bipolar distribution, in the Holarctic region most records originating from Europe. Widely distributed in the Alps (Nimis et al. 2018), but still under-collected country-wide.

Elixia flexella (Ach.) Lumbsch (Elixiaceae)
Piemonte • Cuneo, by the road from Monesi to Úpega, small valley S above the village Úpega, 44°06'55''N, 7°43'20''E, 1600 m, on rotting stumps of *Larix*, 18 July 2000, leg. J. Hafellner (with P. L. Nimis & M. Tretiach), det. J. Hafellner, GZU (JH88547). • Cuneo, Marguareis Natural Park, NE slopes of Mt. Bertrand, W above Úpega, 44°07'40''N, 7°41'30''E, 1960 m, on rotten stumps of *Larix*, 20 July 2000, leg. J. Hafellner (with P. L. Nimis & M. Tretiach), det. J. Hafellner, GZU (JH87607).
Valle d'Aosta • Aosta, by the road from Aosta to the Colle del Gran San Bernardo, NW above Saint Rhémy-en-Bosses, 45°50'25''N, 7°10'40''E, 1800 m, on wood of *Larix* stumps, 1 August 2001, leg. J. Hafellner (with P. L. Nimis & M. Tretiach), det. J. Hafellner, GZU (JH87200).
Due to the almost entirely endoxylic thallus this lignicolous species with minute, sessile, black, elongate to angular apothecia at first view might be taken as non-lichenized fungus. It shows a strong preference for subvertical faces of rotting stumps of conifers, where it often grows together with species of *Calicium*, *Placynthiella*, *Xylographa* and *Cladonia digitata*. The species is widely distributed in Europe from the boreal to the temperate-montane-subalpine belts. In the Alps the distribution appears to be incompletely documented (Nimis et al. 2018), being still undrcollected in Italy, Switzerland and France.

Farnoldia dissipabilis (Nyl.) Hertel (Lecideaceae)
Piemonte • Cuneo, ridge W above Colle del Vallonetto (Vallone dell'Arma), 44°23'10''N, 7°07'00''E, 2500 m, on calcareous rocks, 23 July 2000, leg. P. L. Nimis & M. Tretiach (with J. Hafellner), det. P. L. Nimis, TSB (34058).
On calciferous rocks, especially in rock fissures and on steeply inclined to slightly overhanging surfaces near or above tree line; very closely related to *F. jurana*, this taxon, known from the central European mountains (Alps, Carpathians), awaits further study.

Farnoldia jurana* subsp. *bicincta (Hertel) Hafellner & Türk (Lecideaceae)

Piemonte • Cuneo, Marguareis Natural Park, Pass between Cima di Pertegue (Cima di Pertegà) and Rocca del Fera, W above Upega, 44°08'50"N, 7°41'30"E, 2200 m, on calcareous schists, 20 July 2000, leg. P. L. Nimis & M. Tretiach (with J. Hafellner), det. P. L. Nimis, TSB (34783).
On exposed calcareous rocks near and above tree line, often associated with *Hymenelia coerulea*; probably more widespread in the Alps.

Farnoldia jurana* subsp. *muverani (Müll. Arg.) Hafellner & Türk (Lecideaceae)
Veneto • Belluno, Dolomiti Bellunesi Ntl Park, Erera, Pelse, near the well PE10, 46°09'44"N, 12°00'33"E, 1887 m, on carbonatic rocks, 18 July 2025, leg. et det. J. Nascimbene, Hb. Nascimbene (10289).
On calciferous rocks, including calcareous schists, near and above treeline; to be looked for further in the Alps.

Farnoldia muscigena (Vězda) Hafellner & Tretiach (Lecideaceae)
Piemonte • Cuneo, western ridge of Mt. Nebin ca. 1 Km E of Colle di Sampeyre, 44°32'40"N, 7°08'20"E, 2380 m, on soil and plant debris, 26 July 2000, leg. P. L. Nimis & M. Tretiach (with J. Hafellner), det. P. L. Nimis, TSB (32924).
Known from the central European mountains (Tatra, Alps) this lichen is found on dying bryophytes, crustose lichens and plant debris over calcareous substrata above or near tree line; probably more widespread in the Alps.

Flavoplaca coronata (Körb.) Arup, Frödén & Søchting (Teloschistaceae)
Piemonte • Cuneo, ridge SE above Colle Valcavera-Vallone dell'Arma, 44°22'40"N, 7°06'15"E, 2440 m, on calciferous schists, 23 July 2000, leg. P. L. Nimis & M. Tretiach (with J. Hafellner), det. P. L. Nimis, TSB (33957).
A mild-temperate to subtropical, mainly Mediterranean lichen found on the top of sun-exposed calcareous boulders, in sites often visited by birds; much overlooked in the past and certainly more common, exceptionally reaching the subalpine belt on south-facing rocks in dry-continental Alpine valleys.

Flavoplaca polycarpa (A. Massal.) Arup, Frödén & Søchting (Teloschistaceae)
Liguria • Imperia, Alpi Liguri Natural Park, just E below Upega on right bank of the creek Negrone (Corvo), 44°07'35"N, 7°44'10"E, 1260 m, on calciferous rocks, 19 July 2000, leg. P. L. Nimis & M. Tretiach (with J. Hafellner), det. P. L. Nimis, TSB (33572).
A calcicolous species found in sheltered situations, with optimum in open woodlands, in the Mediterranean belt confined to more humid-shaded situations, growing on the thalli of *Bagliettoa*-species with an involucrellum, especially *B. parmigerella* and *B. parmigera*.

Flavopunctelia flaventior (Stirt.) Hale (Parmeliaceae)
Lombardia • Sondrio, Pian Gembro Natural Reserve, Pian Gembro, 46°09'55"N, 10°10'08"E, 1370 m, on branches of *Betula* sp., 25 November 2023, leg. et det. G. Gheza, Hb. Gheza (23112501). • Como, Torre del Soccorso above Sala Comacina, 45°58'10"N, 9°10'15"E, 336 m, on bark of *Olea europaea*, 24 February 2026, leg. et det. G. Gheza, Hb. Gheza.
Liguria • Genova, near Portofino, 44°18'42"N, 9°12'18"E, 200 m, on bark of broad-leaved trees, det. P. L. Nimis, TSB (21799).
An epiphytic species of rather continental areas, found on more or less isolated deciduous trees, most frequent in dry Alpine valleys.

Frutidella furfuracea (Anzi) M.Westb. & M.Svensson (Ramalinaceae)
Valle d'Aosta • Aosta, Val Veny W of Courmayeur, Pian Veny near la Visaille, 45°47'40"N, 6°54'50"E, 1650 m, on bark of *Picea abies* near the ground, 30 July 2001, leg. J. Hafellner (with P. L. Nimis & M. Tretiach), det. J. Hafellner, GZU (JH87093). • Aosta, by the road from Aosta to the Colle del Gran San Bernardo, NW above Saint Rhémy-en-Bosses, 45°50'25"N, 7°10'40"E, 1800 m, on wood of *Larix* stumps, 1 August 2001, leg. J. Hafellner (with P. L. Nimis & M. Tretiach), det. J. Hafellner, GZU (JH87199).

Liguria • Imperia, slopes SW above the village Monesi, 44°04'20''N, 7°44'10''E, 1630 m, on rotten stumps, 18 July 2000, leg. J. Hafellner (with P. L. Nimis & M. Tretiach), det. J. Hafellner, GZU (JH87491).
For a long time this species has been included under various names in either *Lecidea* or *Biatora* until the congenericity with the type of *Frutidella*, *f. caesioatra* has been established (Schmull et al. 2011, under the heterotypic synonym *F. pullata*). Although sorediate, thalli of *Frutidella furfuracea* are quite frequently fertile. The presence of sphaerophorin is diagnostic (Tønsberg 1992, *sub Lecidea pullata*) against other sorediate crustose lichens. Most populations establish on bark or wood close to the ground. The species is widespread in the Holarctic region and fairly common in the Alps, from the montane to the subalpine belt.

Fuscopannaria praetermissa (Nyl.) P.M. Jørg. (Pannariaceae)
Emilia-Romagna • Reggio Emilia, Appennino Tosco-Emiliano Ntl Park, Mt. Casarola Anticima Nord, 44°19'59''N, 10°12'28''E, 1963 m, on terricolous bryophytes, 29 May 2024, leg. J. Nascimbene, C. Pistocchi, L. Di Nuzzo, L. Francesconi & G. Gheza, det. S. Girlanda & C. Pistocchi, Hb. Nascimbene (9672).
An arctic-alpine to boreal-montane, circumpolar lichen found on calciferous soil, mosses and plant debris, with optimum near and above tree line.

Gabura fascicularis (L.) P.M. Jørg. (Arctomiaceae)
Friuli Venezia Giulia • Udine, N-exposed slopes S above Lago di Sauris, Bosco della Stua, 46°26'35''N, 12°42'50''E, 1100 m, on bark of *Acer pseudoplatanus*, 16 August 1994, leg. et det. J. Hafellner, GZU (JH77024).
For a long time the species has been included in *Collema*, but already Degelius (1954) pointed to its isolated position in this genus. More recently, Otálora and Wedin (2013) have demonstrated a position in *Arctomiaceae*. The species is occasionally found in *Lobarion* communities, mainly on trunks of deciduous broad-leaved trees, in the Alps from the lowlands up to the montane belt. As in recent years in Northern Italy a decline of populations is observed (Nimis 2026), the record published here might already be historic.

Glaucomaria bicincta (Ramond) S.Y. Kondr., Lőkös & Farkas **var. *bicincta*** (Lecanoraceae)
Veneto • Belluno, Pordoi, between Rifugio Vièl dal Pan and Rifugio Fredarola, 46°28'34''N, 11°49'42''E, 2552 m, on volcanic rocks, 8 August 2024, leg. et det. J. Nascimbene, Hb. Nascimbene (9491). • Belluno, Dolomiti Bellunesi Ntl Park, Erera-Piani Eterni, Col Dorin, 46°09'50''N, 12°00'43''E, 1974 m, on selciferous carbonatic rocks, 18 July 2025, leg. et det. J. Nascimbene, Hb. Nascimbene (9992, 10008).
A holarctic lichen found on vertical or rain-sheltered surfaces of hard siliceous rocks.

Glaucomaria bicincta* var. *sorediata (Flot.) ined. (Lecanoraceae)
Piemonte • Cuneo, crest SW above Colle dell'Agnello, 44°40'58''N, 6°58'39''E, 2830 m, on calciferous schists, 25 July 2000, leg. P. L. Nimis & M. Tretiach (with J. Hafellner), det. P. L. Nimis, TSB (33165).
Liguria • Imperia, Alpi Liguri Natural Park, mountains N above Triora on the road to Col Garezzo, 44°02'50''N, 7°45'15''E, 1650 m, on sandstone, 21 July 2000, leg. P. L. Nimis & M. Tretiach (with J. Hafellner), det. P. L. Nimis, TSB (33474).
On vertical or rain-sheltered surfaces of siliceous rocks, probably widespread throughout the Alps, but overlooked, and certainly not common; also known from the Mediterranean mountains.

Glaucomaria rupicola* subsp. *rupicola* var. *efflorens (Leuckert & Poelt) ined. (Lecanoraceae)
Piemonte • Cuneo, Monviso Natural Park, N slopes of Monviso, W above Crissolo just W above Lago Fiorenza (S above Pian del Re), 44°41'50''N, 7°05'35''E, 2130 m, on ophiolitic rocks, 25 July 2021, leg. P. L. Nimis & M. Tretiach (with J. Hafellner), det. P. L. Nimis, TSB (35252).
A silicicolous lichen with optimum in somehow more shaded-humid situations than the typical variety; probably more widespread in the Alps.

Glaucomaria rupicola* subsp. *subplanata (Nyl.) Dose & Nimis (Lecanoraceae)

Veneto • Belluno, Dolomiti Bellunesi Ntl Park, North side of Mt. Talvena, 46°16'03"N, 12°09'21"E, 2437 m, on selciferous carbonatic rocks, 24 June 2025, leg. et det. J. Nascimbene, Hb. Nascimbene (9991).
Liguria • Imperia, Alpi Liguri Natural Park, mountains N above Triora on the road to Col Garezzo, 44°02'50"N, 7°45'15"E, 1650 m, on sandstone, 21 July 2000, leg. P. L. Nimis & M. Tretiach (with J. Hafellner), det. P. L. Nimis, TSB (33472).
More southern and perhaps more xerophytic than the typical subspecies, this taxon should be looked for further throughout Italy.

Glaucomaria rupicola* subsp. *sulphurata (Ach.) ined. (Lecanoraceae)
Piemonte • Cuneo, crest SW above Colle dell'Agnello, 44°40'58"N, 6°58'39"E, 2830 m, on calciferous schists, 25 July 2000, leg. P. L. Nimis & M. Tretiach (with J. Hafellner), det. P. L. Nimis, TSB (33135).
Valle d'Aosta • Aosta, Passo del Piccolo San Bernardo, 45°41'47"N, 6°53'55"E, 2050 m, on siliceous rocks, September 1997, leg. et det. P. L. Nimis, TSB (29483).
Liguria • Imperia, Alpi Liguri Natural Park, ridge S above Monesi, W of Col Garezzo, 44°02'50"N, 7°46'22"E, 1850 m, on calciferous schists, 21 July 2000, leg. P. L. Nimis & M. Tretiach (with J. Hafellner), det. P. L. Nimis, TSB (33390).
More southern, more photo- and thermophilous than the typical subspecies; in the Alps it is common only in dry situations, such as on south-exposed faces.

Glypholecia scabra (Pers.) Müll. Arg. (Acarosporaceae)
Liguria • Imperia, Alpi Liguri Natural Park, ridge S above Monesi, W of Col Garezzo, 44°02'50"N, 7°46'22"E, 1850 m, on calciferous schists, 21 July 2000, leg. P. L. Nimis & M. Tretiach (with J. Hafellner), det. P. L. Nimis, TSB (33358).
An incompletely holarctic species of dry-continental areas, found on exposed surfaces of calciferous and base-rich siliceous rocks; in Italy this characteristic species with an umbilicate thallus is restricted to the dry Alpine valleys with a continental climate.

Graphis betulina (Pers.) Ach. (Graphidaceae)
Trentino-Alto Adige • Trento, Adamello-Brenta Natural Park, Val Brenta, 46°12'01"N, 10°50'11"E, 1260 m, on bark of *Salix* sp., 9 August 2022, leg. G. Gheza & C. Pistocchi, det. G. Gheza, Hb. Gheza (22080937).
A taxon of the *G. scripta*-group with apothecia additionally surrounded by conspicuous, often raised, white thalline margins, found on the bark of broad-leaved trees in various forest types; widely distributed in the Holarctic region.

Graphis pulverulenta (Pers.) Ach. (Graphidaceae)
Emilia-Romagna • Forlì-Cesena, Foreste Casentinesi, Monte Falterona e Campigna Ntl Park, Piana dei Romiti, 43°59'25"N, 11°38'39"E, 720 m, on bark of *Carpinus betulus*, 11 November 2021, leg. C. Pistocchi &. P. Sbardella, det. J. Nascimbene, Hb. Nascimbene (8039).
A taxon of the *G. scripta*-group, characterised by apothecia with mostly acute ends and widely exposed white to grey pruinose discs, found on the bark of broad-leaved trees in various forest types.

Gyalecta geoica (Ach.) Ach. (Gyalectaceae)
Piemonte • Cuneo, ridge W above Colle del Vallonetto (Vallone dell'Arma), 44°23'10"N, 7°07'00"E, 2500 m, on calciferous soil, 23 July 2000, leg. P. L. Nimis & M. Tretiach (with J. Hafellner), det. P. L. Nimis, TSB (34069).
A cool-temperate to arctic-alpine, circumpolar species found on soil, bryophytes and plant debris over calcareous or basic siliceous substrata, often in rock fissures in sheltered situations, mostly in upland areas.

Gyalidea asteriscus (Anzi) Aptroot & Lücking (Gomphillaceae)
Piemonte • Cuneo, mountain ridge NW above Crissolo, N above Pian di Melzè, 44°42'15"N, 7°07'05"E, 1880 m, on calciferous soil, 25 July 2021, leg. P. L. Nimis & M. Tretiach (with J. Hafellner), det. P. L. Nimis,

TSB (35294). • Cuneo, *ibidem*, 25 July 2021, leg. J. Hafellner (with P. L. Nimis & M. Tretiach), det. J. Hafellner, GZU (JH75614).
A typical lichen of steppe grasslands on loess, whose distribution extends widely into Central Asia, found on loess and (in the Alps) on soil deriving from calcareous schists; restricted to strongly continental, dry Alpine valleys in Italy.

Gyalideopsis piceicola (Nyl.) Vězda & Poelt (Gomphillaceae)
Trentino-Alto Adige • Trento, Paneveggio-Pale di San Martino Natural Park, Val Canali, path along Rio Pradidali, 46°13'20"N, 11°52'19"E, 1263 m, on decorticated log, 4 July 2025, leg. et det. Martin Westberg, Hb. UPS (L-1184294), Hb. Nascimbene (10056).
In Italy, the species was previously known only from the mountains of Calabria, on twigs of *Abies* in a damp forest. This is the first record for the Italian Alps.

Gyrographa gyrocarpa (Flot.) Ertz & Tehler (Roccellaceae)
Piemonte • Cuneo, Colle di Sampeyre just W of the Pass, 44°33'10"N, 7°06'55"E, 2300 m, on siliceous rocks, 26 July 2000, leg. P. L. Nimis & M. Tretiach (with J. Hafellner), det. P. L. Nimis, TSB (32845).
On steeply inclined to rain-sheltered surfaces of siliceous rocks, often within forests in cold-humid situations, more rarely on subacid bark.

Helocarpon crassipes Th.Fr. (Helocarpaceae)
Lombardia • Sondrio, Stelvio Ntl Park, trail between Passo del Gavia and Rifugio Berni, 46°20'58"N, 10°29'33"E, 2610 m, on soil and plant debris, 14 September 2021, leg. G. Gheza, det. G. Gheza & F. Bottegoni, Hb. Gheza (21091405).
On bryophytes and plant debris on the ground and amongst rocks, in areas with siliceous substrata, with optimum near tree line; probably more widespread in the Alps. Türk and Hafellner (1993) stated that all material from at least the Eastern Alps is morphologically different from *H. crassipes* s. str., and belongs instead to *H. pulverulum*. However, Myllys et al. (2026) state that the two taxa represent a continuum from specimens with a poorly developed to those with a well-developed thallus, intermediates being sometimes found within the same collection.

Hertelidea botryosa (Fr.) Printzen & Kantvilas (Stereocaulaceae)
Friuli Venezia Giulia • Udine, Bosco della Stua above the Lago di Sauris between La Maina and Sauris/Zahre, 46°26'39"N, 12°43'03"E, 1040 m, on bark of *Abies alba*, 26 September 1990, leg. P. L. Nimis & M. Tretiach, det. P. L. Nimis, TSB (15074).
A probably circumboreal-montane to cool-temperate lichen found on lignum, often on burnt trunks of conifers and oaks, more rarely on acid bark, usually in upland areas but below tree line.

Hydropunctaria scabra (Vězda) C. Keller, Gueidan & Thüs (Verrucariaceae)
Friuli Venezia Giulia • Udine, Paluzza, above Malga Promosio, 46°35'36"N, 13°02'12"E, 1700 m, on stable stones in a small stream crossing the road, on acidic Palaeozoic rocks, 20 September 2022, leg. et det. J. Nascimbene, Hb. Nascimbene (7997).
On siliceous rocks in the amphibious zone of streams, often near rapids and waterfalls, but also on lake shores; widespread in Europe but only locally abundant; with a few records from the Alps, but perhaps overlooked or misidentified as *H. rheitrophila*.

Hymenelia coerulea A. Massal. (Hymeneliaceae)
Friuli Venezia Giulia • Udine, Natura 2000 Site - SAC Conca di Fusine, Laghi di Fusine near Tarvisio, 46°28'10"N, 13°41'18"E, 1700 m, on calcareous rocks, 6 November 1983, leg. M. Palma, det. P. L. Nimis, TSB (3751).

On steeply inclined surfaces of hard calciferous rocks, including moderately dolomitic, hard limestone; certainly widespread and locally abundant throughout the Alps, with optimum in the montane and subalpine belts.

Hymenelia cyanocarpa (Anzi) Lutzoni (Hymeneliaceae)
Veneto • Belluno, Dolomiti Bellunesi Ntl Park, Vette Feltrine, Busa delle Vette, 46°05'55"N, 11°50'27"E, 1900 m, on carbonatic rocks, leg. et det. J. Nascimbene, Hb. Nascimbene (10471).
On periodically inundated, hard siliceous rocks, with optimum above treeline: probably more widespread in the Alps.

Hymenelia heteromorpha (Kremp.) Lutzoni (Hymeneliaceae)
Piemonte • Cuneo, western ridge of Mt. Nebin ca. 1 Km E of Colle di Sampeyre, 44°32'40"N, 7°08'20"E, 2380 m, on calciferous rocks, 26 July 2000, leg. P. L. Nimis & M. Tretiach (with J. Hafellner), det. P. L. Nimis, TSB (32951).
A probably holarctic species found on dolomite and hard limestone in rather sheltered situations, with optimum near tree line.

Ingvariella bispora (Bagl.) Guderley & Lumbsch (Stictidaceae)
Liguria • Imperia, Alpi Liguri Natural Park, mountains N above Triora on the road to Col Garezzo, 44°02'50"N, 7°45'15"E, 1650 m, on sandstone, 21 July 2000, leg. P. L. Nimis & M. Tretiach (with J. Hafellner), det. P. L. Nimis, TSB (33443).
On base-rich siliceous substrata, mostly on horizontal surfaces; most common in Mediterranean Italy but also found in the dry-continental Alpine valleys.

Japewia tornoënsis (Nyl.) Tønsberg (Ramalinaceae)
Valle d'Aosta • Aosta, Passo del Piccolo San Bernardo, 45°41'47"N, 6°53'55"E, 2050 m, on bark, September 1997, leg. et det. P. L. Nimis, TSB (29475).
A circumboreal-montane species found on twigs of shrubs, on terricolous mosses and plant debris in upland areas, usually over siliceous substrata; certainly widespread throughout the Alps.

Kiliasia tristis (Müll. Arg.) Hafellner (Ramalinaceae)
Piemonte • Cuneo, ridge SE above Colle Valcavera-Vallone dell'Arma, 44°22'40"N, 7°06'15"E, 2440 m, on calciferous schists, 23 July 2000, leg. P. L. Nimis & M. Tretiach (with J. Hafellner), det. P. L. Nimis, TSB (33968).
On more or less calcareous substrata in upland areas.

Kuettlingeria percrocata (Arnold) I.V. Frolov, Vondrák & Arup (Teloschistaceae)
Piemonte • Cuneo, cirque E below summit of Rocca dell'Abisso W of Col di Tenda NW below Fort de Giaure, 44°08'25"N, 7°31'05"E, 2250 m, on gneiss, 22 July 2000, leg. P. L. Nimis & M. Tretiach (with J. Hafellner), det. P. L. Nimis, TSB (34457)
On base-rich and lime-containing siliceous rocks near or above tree line.

Lecania olivacella (Nyl.) Zahlbr. (Ramalinaceae)
Piemonte • Cuneo, western ridge of Mt. Nebin ca. 1 Km E of Colle di Sampeyre), 44°32'40"N, 7°08'20"E, 2380 m, on siliceous base-rich rocks (ophiolites), 26 July 2000, leg. P. L. Nimis & M. Tretiach (with J. Hafellner), det. P. L. Nimis, TSB (32902).
A widespread, but rare species found on basic or calciferous siliceous rocks, with a wide altitudinal range; to be looked for further in the Alps.

Lecanora cenisia Ach. **f. *cenisia*** (Lecanoraceae)

Liguria • Imperia, Alpi Liguri Natural Park, mountains N above Triora on the road to Col Garezzo, 44°02’50”N, 7°45’15”E, 1650 m, on sandstone, 21 July 2000, leg. P. L. Nimis & M. Tretiach (with J. Hafellner), det. P. L. Nimis, TSB (33425). • Imperia, *ibidem*, 21 July 2000, leg. J. Hafellner (with P. L. Nimis & M. Tretiach), det. J. Hafellner, GZU (JH87318).
A circumpolar, cool-temperate to arctic-alpine lichen of siliceous rocks, more rarely found also on hard lignum, most frequent in upland areas; common and widespread in the Alps, becoming rarer along the Apennines, south to the mountains of Sicily.

Lecanora epibryon* var. *bryopsora Doppelb. & Poelt (Lecanoraceae)
Veneto • Belluno, Dolomiti Bellunesi Ntl Park, Vette Feltrine, Busa delle Vette, 46°05’55”N, 11°50’27”E, 1900 m, on plant debris, leg. et det. J. Nascimbene, Hb. Nascimbene (10472).
Piemonte • Cuneo, Marguareis Natural Park, Cima di Pertegue (Cima di Pertegà), W above Upega just E below the summit, 44°08’50”N, 7°40’50”E, 2360 m, on soil over calcareous substrata, 20 July 2000, leg. P. L. Nimis & M. Tretiach (with J. Hafellner), TSB (33081). • Cuneo, crest SW above Colle dell'Agnello, 44°40’58”N, 6°58’39”E, 2770 m, on plant remnants, 25 July 2000, leg. J. Hafellner (with P. L. Nimis & M. Tretiach), det. J. Hafellner, GZU (JH59413). • Cuneo, mountain ridge NW above Crissolo, N above Pian di Melzè, 44°42’15”N, 7°07’05”E, 1880 m, on plant remnants, 25 July 2001, leg. J. Hafellner (with P. L. Nimis & M. Tretiach), det. J. Hafellner, GZU (JH75610). • Torino, mountain ridge S of the Valle di Susa, SW above the Colle dell'Assietta, 45°03’55”N, 6°57’10”E, 2550 m, on plant remnants, 27 July 2001, leg. J. Hafellner (with P. L. Nimis & M. Tretiach), det. J. Hafellner, GZU (JH75580).
On mosses and plant debris on calcareous substrata; certainly more widespread in the Alps, but difficult to recognize, being often sterile. According to Malícek (in litt.), molecular data suggest that this is just a sorediate morph of var. *epibryon* (Nimis 2026).

Lecanora handelii J.Steiner (Lecanoraceae)
Friuli Venezia Giulia • Udine, Mt. Coglians Group, surroundings of Comeglians, Mt. Crostis N above Tualis, on E crest N above Casera Chiadinis, 46°34’15”N, 12°53’40”E, 2200 m, on subvertical rock faces of iron-rich schist, 27 July 1995, leg. et det. J. Hafellner, GZU (JH35337).
The mostly sterile species is characterized by scattered greyish-yellowish areoles (much paler than in *L. subaurea*) on which marginal soralia with whitish to greyish soredia (not distincly yellow as in *L. subaurea*) develop. It grows preferably on steep to overhanging faces of metal-rich siliceous rocks and is widely distributed, with most records from various parts of Europe including the Alps and there mostly at high elevations (Nimis et al. 2018).

Lecanora leptacinella Nyl. (Lecanoraceae)
Lombardia • Brescia, Adamello Ragional Park, Passo Gallinera c. 7 km S above Vezza d’Oglio, NW above the pass in the lowermost part of the ridge to Mt. Aviolo, 46°10’55”N, 10°24’45”E, 2340 m, on plant remnants (dying *Polytrichum*), 25 July 2006, leg. J. Hafellner (with J. Nascimbene, L. Muggia & M. Tretiach), det. J. Hafellner, GZU (JH88503).
Piemonte • Cuneo, Monviso Natural Park, Mt. Viso, N slopes W above Crissolo, E above the northern end of Lago Fiorenza (S above Pian del Re), 44°41’50”N, 7°05’35”E, 2130 m, on dying *Rhacomitrium* mats, 25 July 2001, leg. J. Hafellner (with P. L. Nimis & M. Tretiach), det. J. Hafellner, GZU (JH87784).
The species shows a strong preference for dying gametophytes of mosses over acidic soils. Elsewhere it has also been found on branches of *Rhododendron*, detritus and dying lichens. It appears to have an arctic-alpine, holarctic distribution. In the Alps, it is known from the tree line ecotone up to the high-alpine belt, with most records from Austria. In Italy, the species was so far only recorded from one locality in the Dolomites in Trentino-Alto Adige (Nascimbene et al. 2021), but its ecological niche is likely to be under-collected.

Lecanora reagens Norman (Lecanoraceae)

Piemonte • Cuneo, Monviso Natural Park, N slopes of Monviso, W above Crissolo just W above Lago Fiorenza (S above Pian del Re), 44°41’50”N, 7°05’35”E, 2130 m, on ophiolitic rocks, 25 July 2021, leg. P. L. Nimis & M. Tretiach (with J. Hafellner), det. P. L. Nimis, TSB (35216).
Beneath overhangs of hard, mineral-rich siliceous rocks (gneiss, schists), mostly in fissures, occasionally on epilithic mosses, with optimum in upland areas.

Lecanora soralifera (Suza) Räsänen (Lecanoraceae)
Trentino-Alto Adige • Trento, Stelvio Natural Park, c. 8 km N above Cogolo, by the trail from Malga Mare to Lago Lungo, S below the lake, 46°25’25”N, 10°41’15”E, 2520 m, on inclined rock faces of boulders of iron-rich siliceous rocks, 27 July 2006, leg. J. Hafellner (with L. Muggia & M. Tretiach), det. J. Hafellner, GZU (JH88601).
Among the sorediate *Lecanora* species regularly found on metal-rich siliceous rocks *L. soralifera* is characterized by flat, pale yellow, densely aggregated areoles. Occasionally developed apothecia show that the species is closely related to *Lecanora intricata*. It mostly grows on subhorizontal to moderately inclined rock faces of a wide range of various siliceous rocks, often close to the ground. The species is widely distributed in Europe, including the Alps (Nimis et al. 2018). Its rarity in Italy is likely only virtual, due to a still fragmentarily documented distribution.

Lecanora stenotropa Nyl. (Lecanoraceae)
Veneto • Belluno, Dolomiti Bellunesi Ntl Park, Pezedei, below Forcella del Zità Sud, 46°16’27”N, 12°09’13”E, 2285 m, on selciferous carbonatic rocks, 2 July 2024, leg. et det. J. Nascimbene, Hb. Nascimbene (9448). • Belluno, Arabba, N-facing slope of Porta Vescovo, start of the via ferrata, 46°28’24”N, 11°52’25”E, 2610 m, on volcanic rocks, 1 August 2025, leg. et det. J. Nascimbene, Hb. Nascimbene (10304).
This silicicolous species, which is closely related to *L. polytropa*, is apparently quite widespread and locally common along the southern Alps (Roux and coll. 2014). In the past, it might have been confused with *L. polytropa.*

Lecanora subaurea Zahlbr. (Lecanoraceae)
Trentino-Alto Adige • Bolzano, Val Martello (Martelltal), S of Morter, 46°35’45”N, 10°49’25”E, 800 m, on iron-rich siliceous boulders, 14 September 1970, leg. et det. J. Poelt, GZU (JP8867). • Bolzano, Vallelunga (Langtauferer Tal) E of Passo di Resia (Reschenpass), E of Melag, 46°50’10”N, 10°39’40”E, 1920 m, on iron-rich siliceous boulders, 18 April 1984, leg. et det J. Hafellner GZU (JH12386). • Trento, Stelvio Ntl Park, Val del Saent, “Sentiero delle Cascate”, 46°26’15”N, 10°46’07”E, 1700 m, on iron-rich siliceous rocks, August 2003, leg. et det. J. Nascimbene, Hb. Nascimbene (788).
Piemonte • Cuneo, Vallone di S. Anna SW above Vinadio by the road to Colle della Lombarda, 44°16’25”N, 7°08’05”E, 1340 m, on iron-rich siliceous rocks, 24 July 2000, leg. J. Hafellner (with P. L. Nimis & M. Tretiach), det. J. Hafellner, GZU (JH87701). • Cuneo, Monviso Natural Park, N slopes of Monviso, W above Crissolo just W above Lago Fiorenza (S above Pian del Re), 44°41’50”N, 7°05’35”E, 2130 m, on ophiolitic rocks, 25 July 2021, leg. P. L. Nimis & M. Tretiach (with J. Hafellner), det. P. L. Nimis, TSB (35219).
Valle d’Aosta • Aosta, Val Veny W of Courmayeur, moraines E of Lago di Combal, 45°46’35”N, 6°52’10”E, 1980 m, on erratic boulder of iron-rich schist, 30 July 2001, leg. J. Hafellner (with P. L. Nimis & M. Tretiach), det. J. Hafellner, GZU (JH88597).
A typical species of iron-rich rocks, including pebbles.

Lecanora umbrosa Degel. (Lecanoraceae)
Toscana • Pistoia, surroundings of Abetone, Val di Luce, Alpe Tre Potenze, along the lift route “La Fariola”, 44°07’21”N, 10°38’19”E, 1780 m, on slightly calciferous sandstone, 27 October 1978, leg. et det. H. Mayrhofer, GZU (HM490, HM22016).
The sterile specimens occur on slightly calciferous sandstone. This is the first record from the Apennines.

Lecanoropsis albellula (Nyl.) Ivanovich (Lecanoraceae)

Veneto • Belluno, Casera Razzo 1 Km from the border between Friuli and Veneto, 46°28’36”N, 12°37’14”E, 1700 m, on bark of *Larix*, April 1986, leg. et det. P. L. Nimis, TSB (7812).
A probably circumboreal-montane species of hard lignum and acid bark, usually in upland areas, with a mainly western distribution in Europe; certainly widespread throughout the Alps, and becoming rarer southwards.

Lecanoropsis saligna (Schrad.) Ivanovich & Printzen ***var. saligna*** (Lecanoraceae)
Friuli Venezia Giulia • Udine, High Valley of the river Torre, Loc. Vedronza, 46°16’03”N, 13°15’47”E, 386 m, on bark, leg. et det. P. L. Nimis, TSB (12692).
A holarctic, temperate to boreal-montane lichen found on hard, undecomposed wood or on acid bark (conifers, *Betula*, etc.); most frequent in the Alps, but also present in the mountains of Southern Italy.

Lecanoropsis sarcopidoides (A. Massal.) Ivanovich & Printzen (Lecanoraceae)
Friuli Venezia Giulia • Udine, Cima di Valdolce, 46°34’27”N, 13°11’23”E, 1800 m, on bark of broad-leaved trees, 25 May 1995, leg. F. Bersan, det. P. L. Nimis, TSB (20607).
A rather poorly known taxon found on acid bark, most often of conifers, and on lignum, mostly in upland areas.

Lecidea atrobrunnea (DC.) Schaer. **subsp. *atrobrunnea*** (Lecideaceae)
Veneto • Belluno, Arabba, below Forcella Porta Vescovo, 46°28’35”N, 11°52’08”E, 2380 m, on volcanic rocks, 1 August 2025, leg. et det. J. Nascimbene, Hb. Nascimbene (10223). • Belluno, Arabba, Porta Vescovo, 46°28’24”N, 11°52’25”E, 2610 m, on volcanic rocks, 1 August 2025, leg. et det. J. Nascimbene, Hb. Nascimbene (10303).
Liguria • Imperia, Alpi Liguri Natural Park, Mountains N above Triora on the road to Col Garezzo, 44°02’50”N, 7°45’15”E, 1650 m, on sandstone, 21 July 2000, leg. P. L. Nimis & M. Tretiach (with J. Hafellner), det. P. L. Nimis, TSB (33431).
A bipolar, arctic-alpine to boreal-montane species of acid siliceous rocks in exposed situations, with optimum near and above tree line; common only in the Alps. The species is chemically variable, and several chemotypes were distinguished.

Lecidea berengeriana (A. Massal.) Nyl. (Lecideaceae)
Emilia-Romagna • Reggio Emilia, Appennino Tosco-Emiliano Ntl Park, below Cima dell’Angelo, 44°16’06”N, 10°25’07”E, 1834 m, on soil, 8 July 2025, leg. C. Pistocchi, S. Girlanda & F. Zinato, det. C. Pistocchi, Hb. Nascimbene (10156). • Reggio Emilia, Appennino Tosco-Emiliano Ntl Park, Mt. Prado (summit area), 44°14’59”N, 10°24’22”E, 2037 m, on soil, 12 July 2025, leg. C. Pistocchi, S. Girlanda & F. Zinato, det. S. Girlanda & C. Pistocchi, Hb. Nascimbene (10126).
A circumpolar, arctic-alpine to boreal-montane lichen found on mosses and plant debris over calcareous substrata; most common in the Alps, but probably occurring throughout the Apennines. The species does not belong to *Lecidea* nor to *Mycobilimbia* and is closely related to *Romjularia* (Fryday et al. 2014).

Lecidea confluescens Nyl. (Lecideaceae)
Piemonte • Cuneo, Alpi Liguri Natural Park, N ridge of Mt. Saccarello W above Monesi near Monumento al Redentore, 44°03’45”N, 7°42’50”E, 2200 m, on calciferous siliceous rocks, 18 July 2000, leg. P. L. Nimis & M. Tretiach (with J. Hafellner), det. P. L. Nimis, TSB (33694).
On inclined to vertical faces of calciferous rocks, especially lime-containing schists, with optimum above tree line, up to the nival belt; related to *L. lapicida*, but calcicolous and probably restricted to the Alps, where it is rare.

Lecidea leprosolimbata (Arnold) Poelt (Lecideaceae)
Piemonte • Cuneo, crest SW above Colle dell’Agnello, 44°40’58”N, 6°58’39”E, 2830 m, on calciferous schists, 25 July 2000, leg. P. L. Nimis & M. Tretiach (with J. Hafellner), det. P. L. Nimis, TSB (33193).
On sunny, inclined surfaces of hard, weakly calciferous siliceous rocks in upland areas; certainly more widespread in the Alps, and locally even common.

Lecidea nylanderi (Anzi) Th. Fr. (*incertae sedis*)
Friuli Venezia Giulia • Udine, N-exposed slopes S above Lago di Sauris, Bosco della Stua, 46°26'35"N, 12°42'50"E, 1100 m, on bark of *Picea abies*, 16 August 1994, leg. et det. J. Hafellner, GZU (JH84443).
Piemonte • Cuneo, by the road from Monesi to Úpega, small valley S above the village Úpega, 44°06'55"N, 7°43'20"E, 1600 m, on bark of *Larix*, 18 July 2000, leg. J. Hafellner (with P. L. Nimis & M. Tretiach), det. J. Hafellner, GZU (JH88547).
Valle d'Aosta • Aosta, Val Veny W of Courmayeur, Pian Veny near La Visaille, 45°47'40"N, 6°54'50"E, 1550 m, on bark of *Picea abies* near the ground, 30 July 2001, leg. J. Hafellner (with P. L. Nimis & M. Tretiach), det. J. Hafellner, GZU (JH87080).
The thallus of this mostly sterile species forms a thin crust consisting of greyish-white, relatively solid granules containing divaricatic acid (UV+ white). It is usually found on the bark of conifers, in the Alps at more humid sites, mostly on *Picea abies,* being apparently rarer in the dry-continental valleys. At the cited locality in Valle d'Aosta, it had settled, with *Ochrolechia alboflavescens*, in rather humid and slightly nutrient-enriched situations. The species is widespread from the boreal to the nemoral-montane zone in the Holarctic region, with most records in Europe and North America. Its distribution in Italy is still incompletely documented.

Lecidea obluridata Nyl. (Lecideaceae)
Trentino-Alto Adige • Trento, Paneveggio-Pale di San Martino Natural Park, Cavallazza Piccola, 46°16'45"N, 11°46'51"E, 2290 m, on porphyric rocks, 5 August 2022, leg. J. Nascimbene, det. J. Hafellner, Hb. Nascimbene (9415).
A rather poorly known silicicolous species based on a type from low elevation in the Pyrenees, with several records in Western and Central Europe, and across the Alps.

Lecidea promiscens Nyl. (Lecideaceae)
Friuli Venezia Giulia • Udine, Natura 2000 Site-Creta di Aip e Sella di Lanza, Passo Cason di Lanza, 46°33'22"N, 13°10'06"E, 1800 m, on siliceous rocks, 25 May 1995, leg. F. Bersan & A. Rondi, det. P. L. Nimis, TSB (20586).
A circum- and bipolar, arctic-alpine to boreal-montane lichen (Hertel 2006) found on boulders close to the ground and on siliceous pebbles in alpine heaths; certainly more widespread in the Alps, where it reaches the nival belt, and also reported from the Northern Apennines.

Lecidea rapax Hertel (Lecideaceae)
Piemonte • Cuneo, Rocca dell'Abisso W of Col di Tenda just N below the summit, 44°08'37"N, 7°30'14"E, 2750 m, on gneiss, 22 July 2000, leg. P. L. Nimis & M. Tretiach (with J. Hafellner), det. P. L. Nimis, TSB (34492). • Torino, mountains W of Pinerolo, NE slopes and ridges of the Punta Cialáncia S above the village Perrero, 44°53'00"N, 7°07'20"E, 2350 m, on siliceous rocks, on the thallus of *Bellemerea alpina*, 26 July 2001, leg. J. Hafellner (with P. L. Nimis & M. Tretiach), det. J. Hafellner, GZU (JH69376).
Valle d'Aosta • Aosta, Gran Paradiso Ntl Park, near the trail from Pont to the Rifugio Vittorio Emanuele II, 45°30'50"N, 7°13'17"E, 2550 m, on inclined rock faces (gneiss), on the thallus of *Bellemerea alpina*, 31 July 2001, leg. J. Hafellner (with P. L. Nimis & M. Tretiach), det. J. Hafellner, GZU (JH87032). • Aosta, Colle del Gran San Bernardo, SW below the pass, 45°52'18"N, 7°09'34"E, 2500 m, on siliceous rocks, on the thallus of *Bellemerea alpina*, 1 August 2001, leg. J. Hafellner (with P. L. Nimis & M. Tretiach), det. J. Hafellner, GZU (JH87110).
On siliceous rocks, growing on the thalli of *Bellemerea*-species; closely related to *L. leprosolimbata*, and so far known only from the Alps.

Lecidea speirodes Nyl. (Lecideaceae)
Piemonte • Cuneo, crest SW above Colle dell'Agnello, 44°40'50"N, 6°58'32"E, 2830 m, on hard siliceous rocks, 25 July 2000, leg. P. L. Nimis & M. Tretiach (with J. Hafellner), det. P. L. Nimis, TSB (33167).

A lichen known from the central and southern European mountains (Alps, Pyrenees, Cordillera Cantabrica in Spain, Tatra Mountains), found on steeply inclined, superficially decalcified calciferous rocks or on lime-containing siliceous rocks, with optimum in upland areas, up to the nival belt in the Alps.

Lecidea swartzioidea (Lecideaceae)
Veneto • Belluno, Pordoi, between Rifugio Vièl dal Pan and Rifugio Fredarola, 46°28’35”N, 11°49’29”E, 2492 m, on volcanic rocks, 8 August 2024, leg. et det. J. Nascimbene, Hb. Nascimbene (9488).
A circumpolar, arctic-alpine to boreal-montane lichen of siliceous rocks (Hertel 2006) most common near and above tree line: closely related to *L. lapicida* and doubtfully worthy of being separated from it as a distinct species.

Lecidea tessellata* var. *caesia (Anzi) Arnold (Lecideaceae)
Liguria • Imperia, Alpi Liguri Natural Park, ridge S above Monesi, W of Col Garezzo, 44°02’50”N, 7°46’22”E, 1850 m, on calciferous schists, 21 July 2000, leg. P. L. Nimis & M. Tretiach (with J. Hafellner), det. P. L. Nimis, TSB (33416).
On calciferous siliceous rocks, dolomite, superficially decalcified, hard limestone rocks near and above tree line, starting the life-cycle on other crustose lichens, especially *Aspicilia* sp.

Lecidea tessellata Flörke **var. *tessellata*** (Lecideaceae)
Liguria • Imperia, Alpi Liguri Ntl Park, mountains N above Triora on the road to Col Garezzo, 44°02’50”N, 7°45’15”E, 1650 m, on sandstone, 21 July 2000, leg. P. L. Nimis & M. Tretiach (with J. Hafellner), det. P. L. Nimis, TSB (33420).
A cool-temperate to arctic-alpine, circumpolar lichen found on hard, often mineral-rich siliceous rocks in upland areas, which sometimes starts the life-cycle on other crustose lichens, especially *Aspicilia* sp.; common in the Alps, where it reaches the nival belt, much rarer in the mountains of Southern Italy.

Lecidea umbonata (Hepp) Mudd (Lecideaceae)
Emilia-Romagna • Modena, Alto Appennino Modenese Natural Park, Mt. Cimone between Salto della Capra and Rifugio Pian Cavallaro, 44°12’04”N, 10°42’36”E, 1820 m, on subacidic arenaceous rocks, 21 October 2022, leg. et det. J. Nascimbene, Hb. Nascimbene (8013).
A circumpolar, mainly arctic-alpine, variable species (Hertel 2006) found on calciferous siliceous rocks, especially schist, in cool and humid situations near or above tree line; most frequent in the Alps, where it reaches the nival belt, but also present along the Apennines.

Lecidella flavosorediata (Vězda) Hertel & Leuckert (Lecanoraceae)
Liguria • Imperia, slopes SW above the village Monesi, 44°04’20”N, 7°44’10”E, 1630 m, on bark of *Larix*, 18 July 2000, leg. P. L. Nimis & M. Tretiach (with J. Hafellner), det. P. L. Nimis, TSB (33039).
This epiphytic species seems to be most frequent on deciduous trees and *Abies* in the mountains of Southern Italy, with a few records from the Alps.

Lecidella scabra (Taylor) Hertel & Leuckert (Lecanoraceae)
Piemonte • Cuneo, N ridge of Mt. Saccarello W above Monesi near Monumento al Redentore, 44°03’45”N, 7°42’50”E, 2200 m, on siliceous base-rich rocks, 18 July 2000, leg. P. L. Nimis & M. Tretiach (with J. Hafellner), det. P. L. Nimis, TSB (33660).
Liguria • Imperia, Alpi Liguri Natural Park, ridge S above Monesi, W of Col Garezzo, 44°02’50”N, 7°46’22”E, 1850 m, on calciferous schists, 21 July 2000, leg. P. L. Nimis & M. Tretiach (with J. Hafellner), det. P. L. Nimis, TSB (33407).
A mainly temperate to Mediterranean lichen found on basic siliceous substrata wetted by rain in species-poor stands; quite common on brick in archaeological areas of Tyrrhenian Italy, but also present in the Alps, the species is chemically heterogeneous. Albeit more rarely, the species can also grow on bark at the base of old deciduous trees.

Lecidella wulfenii (Hepp) Körb. (Lecanoraceae)
Emilia-Romagna • Reggio Emilia, Appennino Tosco-Emiliano Ntl Park, Mt. Casarola (sent. 667), 44°19'55''N, 10°12'28''E, 1972 m, on soil, 29 May 2024, leg. J. Nascimbene, C. Pistocchi, L. Di Nuzzo, L. Francesconi & G. Gheza, det. S. Girlanda & C. Pistocchi, Hb. Pistocchi (633).
A circumpolar, arctic-alpine lichen found on dying bryophytes and plant remains in exposed habitats near and above tree line; most common in the Alps, but also present in the high mountains of Southern Italy. This is the first record from the Northern Apennines.

Lecidoma demissum (Rutstr.) Gotth. Schneid. & Hertel (Lecideaceae)
Emilia-Romagna • Bologna, Corno alle Scale Natural Park, below Punta Sofia, 44°07'27''N, 10°49'39''E, 1850 m, on soil, 22 October 2021, leg. et det. J. Nascimbene, Hb. Nascimbene (7819). • Reggio Emilia, Appennino Tosco-Emiliano Ntl Park, Alpe di Succiso, 44°19'59''N, 10°11'47''E, 2010 m, on soil, 29 May 2024, leg. J. Nascimbene, C. Pistocchi, L. Di Nuzzo, L. Francesconi & G. Gheza, det. S. Girlanda & C. Pistocchi, Hb. Nascimbene (9679). • Reggio Emilia, Appennino Tosco-Emiliano Ntl Park, below Sella del Monte Prado, 44°15'11''N, 10°24'00''E, 1892 m, on soil, 12 July 2025, leg. C. Pistocchi, S. Girlanda & F. Zinato, det. C. Pistocchi, Hb. Nascimbene (10220).
A circumpolar, arctic-alpine lichen found on soil, rarely on siliceous rocks, in clearings of alpine grasslands with a long snow-lie; most common in the Alps, but also occurring in the mountains of Calabria. These are the first records for the Northern Apennines.

Lendemeriella lucifuga (G. Thor) S.Y. Kondr. (Teloschistaceae)
Piemonte • Cuneo, Vallone della Stura di Demonte 1 Km E of Festiona, 44°18'05''N, 7°21'30''E, 720 m, on bark of *Castanea* sp., 24 July 2000, leg. P.L. Nimis & M. Tretiach (with J. Hafellner), det. P.L. Nimis, TSB (33493).
A temperate species found on ancient, more or less isolated deciduous trees, especially oaks or *Castanea*, often in crevices of rough bark and on faces seldom wetted by rain.

Lepra schaereri (Hafellner) Hafellner (Ochrolechiaceae)
Veneto • Belluno, Arabba, N-facing slope of Porta Vescovo, 46°28'35''N, 11°52'08''E, 2380 m, on basaltic volcanic rocks, 1 August 2025, leg. et det. J. Nascimbene, Hb. Nascimbene (10263).
On base- or mineral-rich rocks in rainy areas near and above tree line, up to the nival belt in the Alps; probably restricted to the Alps and the Northern Apennines in Italy.

Lepraria borealis Loht. & Tønsberg (Stereocaulaceae)
Lombardia • Bergamo, Orobie Bergamasche Natural Park, road between Cimalbosco and Passo del Vivione, 46°01'35''N, 10°12'07''E, 1762 m, on moss cushions above siliceous sandstone, 31 May 2022, leg. G. Gheza, det. G. Gheza & H. Mayrhofer, Hb. Gheza (22053189). • Bergamo, Orobie Bergamasche Natural Park, trail between Baita Brandelegno and Baita Napoleù, 46°00'34''N, 10°05'38''E, 1522 m, on moss cushions on siliceous sandstone, 17 August 2022, leg. et det. G. Gheza, Hb. Gheza (22081727). • Brescia, Stelvio Ntl Park, between Passo del Gavia and Corno dei Tre Signori, 46°20'44''N, 10°30'07''E, 2710 m, on moss cushions above gneiss rocks and boulders, 24 August 2022, leg. et det. G. Gheza & L. Di Nuzzo, Hb. Gheza (22082403). • Bergamo, Orobie Bergamasche Natural Park, Lago del Venerocolo, 46°03'19''N, 10°09'24''E, 2302 m, on moss cushions above mica-schist boulders, 26 August 2022, leg. et det. G. Gheza & L. Di Nuzzo, Hb. Gheza (22082653). • Bergamo, Orobie Bergamasche Natural Park, trail below Rifugio Tagliaferri, 46°03'12''N, 10°06'46''E, 2330 m, on moss cushions on siliceous sandstone, 17 August 2023, leg. et det. G. Gheza & C. Pistocchi, Hb. Gheza (23081749). • Sondrio, Orobie Valtellinesi Natural Park, trail along Lago Belviso, 46°05'08''N, 10°07'59''E, 1490 m, on moss cushions on a mica-schist outcrop, 22 August 2023, leg. et det. G. Gheza, Hb. Gheza (23082211). • Bergamo, Baita del Quader, 46°00'44''N, 10°07'11''E, 1250 m, on moss cushions on siliceous sandstone, 29 April 2024, leg. et det. G. Gheza, Hb. Gheza (24042912). • Bergamo, Orobie Bergamasche Natural Park, between Pizzo Tornello and Lago di Varro, 46°01'54''N, 10°06'32''E, 2374

m, on moss cushions on siliceous sandstone, 20 July 2024, leg. et det. G. Gheza & L. Di Nuzzo, Hb. Gheza (24072018). • Bergamo, Orobie Bergamasche Natural Park, trail between Lago di Val Asinina and Laghetti delle Valli, 46°02’09”N, 10°10’49”E, 2067 m, on mosses over siliceous sandstone, 5 August 2024, leg. et det. G. Gheza, Hb. Gheza (24080540). • Sondrio, between Rifugio Bignami and Ghiacciaio Fellaria, 46°20’14”N, 9°56’53”E, 2453 m, on moss cushions above schist boulders, 14 August 2024, leg. et det. G. Gheza, Hb. Gheza (24081403). • Bergamo, trail between Ronco and Cascata del Vò, 46°01’22”N, 10°08’37”E, 1136 m, on mosses over siliceous sandstone, 19 August 2024, leg. et det. G. Gheza, Hb. Gheza (24081922). • Brescia, Mt. Culvegla, 46°04'55”N, 10°12'30”E, 2615 m, on moss cushions over schist, 31 August 2025, leg. et det. G. Gheza, Hb. Gheza (25083108). • Brescia, trail between Sellero and Pià d’Ort, 46°02’57”N, 10°20’23”E, 556 m, on mosses over siliceous sandstone, 2 January 2026, leg. et det. G. Gheza, Hb. Gheza (26010206).
A species typically growing on bryophytes on siliceous rocks in montane areas (Nimis 2026), which can occasionally occur also in the lowlands (Gheza and Nascimbene 2024). Despite having never been reported before from Lombardia, it seems quite widespread in the subalpine and alpine belts of the region.

Leprocaulon quisquiliare (Leers) M. Choisy (Catillariaceae)
Emilia-Romagna • Bologna, Piandisetta, along Rio Farnedola near Molino dei Cucchi, 44°15’08”N, 11°10’25”E, 275 m, on bark, 26 May 2023, leg. D. Ivan, det. G. Gheza, Hb. Nascimbene (9950).
A mainly mild-temperate to Mediterranean lichen found on basic siliceous rocks covered by a thin film of soil; very common on brick walls in archaeological areas of Tyrrhenian Italy, where it is also found on bark (e.g. of *Olea*), extremely rare along the eastern side of the Peninsula, exceptionally reaching the montane belt in the western Alps.

Leproplaca chrysodeta (Vain.) Ahti (Teloschistaceae)
Piemonte • Cuneo, ridge NW above Crissolo N above Pian di Melzè, S-exposed slopes, 44°42’15”N, 7°07’05”E, 1880 m, on calciferous schists, 25 July 2001, leg. P. L. Nimis & M. Tretiach (with J. Hafellner), det. P. L. Nimis, TSB (35311*)*.
A temperate to humid subtropical species found on shaded, steeply inclined or rain-sheltered surfaces of calciferous rocks, sometimes also overgrowing epilithic mosses or even occurring on the undersides of inclined old trunks of trees with base-rich bark; certainly ranging throughout the country, below the subalpine belt.

Leproplaca obliterans (Nyl.) Arup, Frödén & Søchting (Teloschistaceae)
Piemonte • Cuneo, Monviso Natural Park, N slopes of Monviso, W above Crissolo just W above Lago Fiorenza (S above Pian del Re), 44°41’50”N, 7°05’35”E, 2130 m, on ophiolitic rocks, 25 July 2021, leg. P. L. Nimis & M. Tretiach (with J. Hafellner), det. P. L. Nimis, TSB (35237).
A cool-temperate to boreal-montane, circumpolar species found under overhangs of basic siliceous rocks, especially calcareous schists, mostly in upland areas; most frequent in the Alps, but also present in the Mediterranean mountains.

Leproplaca proteus (Poelt) Arup, Frödén & Søchting (Teloschistaceae)
Friuli Venezia Giulia • Udine, Sappada, Laghi d’Olbe, 46°35’50”N, 12°41’33”E, 2160 m, on carbonatic rocks, 8 November 2025, leg. et det. J. Nascimbene, Hb. Nascimbene (10002).
On steeply inclined to rain-sheltered surfaces of compact, more or less calcareous rocks in rather sheltered situations, restricted to warm-dry sites in the mountains.

Lichenomphalia hudsoniana (H.S.Jenn.) Redhead, Lutzoni, Moncalvo & Vilgalys (Hygrophoraceae)
Veneto • Belluno, c. 9 km W of Sauris di Sopra, saddle between Col Marende and M. Tiarfin, S above Casera Razzo, 46°28’15”N, 12°36’10”E, 2000 m, overgrowing bryophytes and plant remnants, 27 July 1993, leg. et det. J. Hafellner, GZU (JH32749).
The specimen reported here is sterile. However, as *L. hudsoniana* is the only species in Europe developing a *Coriscium*-type thallus somewhat recalling the squamules of *Normandina pulchella*. In the Alps the species is

fairly common in suitable habitats over acidic soils (including raw humus in N exposition under *Pinus mugo* stands in limestone mountains).

Lobothallia alphoplaca (Wahlenb.) Hafellner (Megasporaceae)
Veneto • Belluno, Arabba, below Forcella Porta Vescovo, 46°28’35”N, 11°52’08”E, 2380 m, on volcanic rocks, 1 August 2025, leg. et det. J. Nascimbene, Hb. Nascimbene (10082).
A widespread species with an apparently disjunct distribution in mountain areas of the Northern hemisphere, found on compact siliceous rocks wetted by rain in upland areas; locally abundant only in the Alps, wherever appropriate substrata are available.

Miriquidica garovaglii (Schaer.) Hertel & Rambold (Lecanoraceae)
Veneto • Belluno, Pordoi, between Rifugio Vièl dal Pan and Rifugio Fredarola, 46°28’34”N, 11°49’42”E, 2552 m, on volcanic rocks, 8 August 2024, leg. et det. J. Nascimbene, Hb. Nascimbene (9483).
A circumpolar, arctic-alpine species found on mineral-rich rocks wetted by rain in wind-exposed situations, such as on peaks and windy ridges in the Alps, usually near or above tree line, reaching the nival belt.

Miriquidica leucophaea (Rabenh.) Hertel & Rambold (Lecanoraceae)
Friuli Venezia Giulia • Udine, Mt. Crostis massiv N of Comeglians, S-slopes of Mt. Neval, 46°33’50”N, 12°53’30”E, 2000 m, on inclined rock faces near the ground (siliceous schists), 17 August 1994, leg. et det. J. Hafellner, GZU (JH76919).
A species resembling *Lecanora polytropa,* but with a very different secondary chemistry (miriquidic acid). It grows on a wide variety of siliceous rocks (e.g. gneiss, mica schist, acidic sandstone, clay slate, eclogite, serpentinite), mostly on low and therefore longer moist outcrops, in boulder fields and on pebbles, being ecologically similar to *Rhizocarpon alpicola*, perhaps occasionally starting the life-cycle on *Rhizocarpon geographicum* s. lat. It has a holarctic distribution with a disjunction in SE Australia; in Europe it ranges from the arctic to the temperate zone, being fairly common on suitable sites in the central European mountains, from the montane to the lower alpine belt. In Italy it is probably more common than the few records would suggest.

Miriquidica nigroleprosa (Vain.) Hertel & Rambold **var. *nigroleprosa*** (Lecanoraceae)
Trentino-Alto Adige • Bolzano, Val d’Ultimo, near Steinbergl Alm and Kaserfeld-Alm, 46°30’39”N, 10°53’27”E, 2038 m, on siliceous rocks, 28 May 2021, leg. et det. H. Oberhollenzer, STU.
On hard siliceous rocks (e.g. granite) in exposed situations such as windy ridges, starting the life-cycle on yellow *Rhizocarpon*-species; most often sterile, it has been largely overlooked in the Alps.

Montanelia disjuncta (Erichsen) Divakar, A. Crespo, Wedin & Essl. (Parmeliaceae)
Emilia-Romagna • Bologna, Corno alle Scale Natural Park, Madonna dell’Acero, 44°09’06”N, 10°49’21”E, 1166 m, on siliceous sandstone, 17 April 2024, leg. et det. G. Gheza & L. Di Nuzzo, Hb. Gheza (24041702).
A widespread lichen of dry-cool areas found on steeply inclined surfaces of siliceous rocks in upland areas; most frequent, but generally not common, in the Alps, very rare in the high Mediterranean mountains.

Nesothele succedens (Rehm ex Arnold) Orange (Verrucariaceae)
Veneto • Belluno, Dolomiti Bellunesi Ntl Park, Cimonega, along Sentiero dei Caserin near Pass de Mura, 46°09’22”N, 11°55’25”E, 1830 m, on dolomitic rocks, 25 June 2023, leg et det. J. Nascimbene, Hb. Nascimbene (8200). • Belluno, Dolomiti Bellunesi Ntl Park, near Malga Pramper close to a spring, 46°17’48”N, 12°09’11”E, 1655 m, on subacidic rocks, 2 July 2024, leg. et det. J. Nascimbene, Hb. Nascimbene (9315). • Belluno, near Passo Pordoi close to a spring above the street, 46°29’07”N, 11°48’57”E, 2219 m, on carbonatic rocks, 8 August 2024, leg. et det. J. Nascimbene, Hb. Nascimbene (9470).
On basic siliceous to calcareous rocks with frequent seepage of water, often near creeks, in gorges etc., mostly in upland areas.

Ochrolechia frigida (Sw.) Lynge (Ochrolechiaceae)

Veneto • Belluno, Pordoi, between Rifugio Vièl dal Pan and Rifugio Fredarola, 46°28’36”N, 11°49’19”E, 2458 m, on acidic soil, 8 August 2024, leg. et det. J. Nascimbene, Hb. Nascimbene (9321). • Belluno, Dolomiti Bellunesi Ntl Park, Mt. Talvena, 46°15’58”N, 12°9’21”E, 2542 m, on plant debris, 24 June 2025, leg. et det. J. Nascimbene, Hb. Nascimbene (10039). • Belluno, Dolomiti Bellunesi Ntl Park, North side of Mt. Talvena, 46°16’01”N, 12°09’21”E, 2460 m, on plant debris, 24 June 2025, leg. et det. J. Nascimbene, Hb. Nascimbene (10040).
An arctic-alpine, circumpolar species found on mosses, plant debris and soil in the Alps, where it reaches the nival belt.

Ochrolechia upsaliensis (L.) A.Massal.(Ochrolechiaceae)
Valle d’Aosta • Aosta, Val Veny W of Courmayeur, ridge W above the Rifugio Elisabetta Soldini, 45°45’55”N, 6°50’05”E, 2250 m, on plant remnants and bryophytes, 30 July 2001, leg. J. Hafellner (with P. L. Nimis & M. Tretiach), det. J. Hafellner, GZU (JH75441).
The always richly fruiting species with an overall holarctic distribution is fairly common at high altitudes in calcareous mountains. In the Alps it preferably settles on plant remnants in cold steppes above the tree line (e.g. *Caricetum firmae* and similar vegetation types).

Opegrapha niveoatra (Borrer) J.R. Laundon (Opegraphaceae)
Lombardia • Sondrio, Bagni di Masino, 46°14’34”N, 9°36’04”E, 1159 m, on bark of *Abies alba*, 24 August 2019, leg. et det. G. Gheza, Hb. Gheza.
A mild-temperate lichen found on old trees in open woodlands; closely related to *O. vulgata*, but differing in the shorter conidia.

Opegrapha vulgata (Ach.) Ach. (Opegraphaceae)
Friuli Venezia Giulia • Udine, highest part of Val Pesarina, Rifugio De Gasperi (Pesariis), 46°32’20”N, 12°45’17”E, 1500 m, on bark, 3 September 1983, det. P.L. Nimis, TSB (3602).
A widespread, but not common temperate species with optimum in humid forests, especially on *Abies alba*, but also on broad-leaved trees.

Pachypeltis insularis (Poelt) Vondrák & I.V.Frolov (Teloschistaceae)
Piemonte • Cuneo, ridge SE above Colle Valcavera-Vallone dell’Arma, 44°22’40”N, 7°06’15”E, 2440 m, on calciferous schists, 23 July 2000, leg. P. L. Nimis & M. Tretiach (with J. Hafellner), det. P. L. Nimis, TSB (33964).
Liguria • Imperia, Alpi Liguri Natural Park, ridge S above Monesi, W of Col Garezzo, 44°02’50”N, 7°46’22”E, 1850 m, on calciferous schists on *Aspicilia candida*, 21 July 2000, leg. P. L. Nimis & M. Tretiach (with J. Hafellner), det. P. L. Nimis, TSB (33395).
On calcareous schists, lichenicolous on *Aspicilia candida* and *A. polychroma*; previously known from South Tyrol, but probably more widespread in the Alps.

Parmelia ernstiae Feuerer & A. Thell (Parmeliaceae)
Trentino-Alto Adige • Trento, Adamello-Brenta Natural Park, between Molveno and Croz dell’Altissimo (forestry road n. 322), 46°10‘05“N, 10°55‘58“E, 1394 m, on bark of *Abies alba*, 24 June 2022, leg. et det. L. Francesconi, Hb. Francesconi (57), ITS-GenBank Acc.Nr: PZ094143. • Trento, Adamello-Brenta Natural Park, Val Nambrone, near Rifugio Nambrone, 46°12’14”N, 10°45’09”E, 1330 m, on bark of *Picea abies*, 20 July 2023, leg. et det. J. Nascimbene, Hb. Nascimbene (8785), ITS-GenBank Acc.Nr: PZ094142.
This mainly epiphytic, cryptic species seems to be quite common in the Iberian Peninsula; the Italian distribution is poorly known, as the species was almost never distinguished from *P. saxatilis* and/or *P. serrana*; several Italian records could be hidden under those of *P. saxatilis* s. lat.

Parmelia pinnatifida Kurok. (Parmeliaceae)

Trentino-Alto Adige • Trento, Adamello-Brenta Natural Park, between Malga Campo and Lago di San Giuliano, 46°09'24"N, 10°42'58"E, 1740 m, on bark, 27 July 2023, leg. et det. G. Gheza, L. Francesconi & C. Pistocchi, Hb. Nascimbene (9615). • Trento, Stelvio Ntl Park, Frattasecca Integral Reserve, 46°21'10"N, 10°38'06"E, 1910 m, on siliceous rocks, 3 October 2024, leg. et det. C. Pistocchi, Hb. Nascimbene (9542). • Trento, Stelvio Ntl Park, Frattasecca Integral Reserve near Cascata Cadini, 46°21'24"N, 10°38'28"E, 1820 m, on siliceous rocks, 3 October 2024, leg. et det. C. Pistocchi, Hb. Nascimbene (9564). • Trento, Stelvio Ntl Park, Val di Rabbi, above Malga Fratte, 46°24'33"N, 10°46'52"E, 1562 m, on epiphytic bryophytes, 19 June 2025, leg. C. Pistocchi, det. C. Pistocchi & G. Gheza, Hb. Nascimbene (10264). • Trento, Stelvio Ntl Park, Val del Monte, Pradacci, 46°20'35"N, 10°37'45"E, 1642 m, on siliceous rocks, 27 August 2025, leg. et det. C. Pistocchi, Hb. Nascimbene (10356).
This species has been only recently reported from the Italian Alps (Gheza 2019); other records may be hidden among those of the morphologically and ecologically similar *P. omphalodes*.

Parmelia serrana A. Crespo, M.C. Molina & D. Hawksw. (Parmeliaceae)
Trentino-Alto Adige • Trento, Val di Sole, between Val Piana and Lago dei Caprioli, 46°18'00"N, 10°45'02"E, 1370 m, on bark of *Picea abies*, 16 June 2025, leg. C. Pistocchi, det. L. Di Nuzzo & C. Pistocchi, Hb. Nascimbene (10461).
This species seems to be widespread in southern Europe, especially in areas with a subcontinental climate. Its Italian distribution is very poorly known, and most records are probably hidden among those of *P. saxatilis* s.lat.

Parvoplaca tiroliensis (Zahlbr.) Arup, Søchting & Frödén (Teloschistaceae)
Piemonte • Cuneo, western ridge of M. Nebin ca. 1 Km E of Colle di Sampeyre, 44°32'40"N, 7°08'20"E, 2380 m, on soil and plant debris, 26 July 2000, leg. P. L. Nimis & M. Tretiach (with J. Hafellner), det. P. L. Nimis, TSB (32964). • Cuneo, *ibidem*, 26 July 2000, leg. J. Hafellner (with P. L. Nimis & M. Tretiach), det. J. Hafellner, GZU (JH75629). • Torino, Gran Bosco di Salbertrand Natural Park, ridge S of the Valle di Susa, SW above Colle dell'Assietta, 45°03'55"N, 6°57'10"E, 2550 m, on plant remnants, 27 July 2001, leg. J. Hafellner (with P. L. Nimis & M. Tretiach), det. J. Hafellner, GZU (JH75578). • Torino, Gran Bosco di Salbertrand Natural Park, ridge S of Valle di Susa, northern slopes of Mt. Genevris, ENE of the top, 45°02'03"N, 6°54'25"E, 2500 m, on plant remnants, 27 July 2001, leg. J. Hafellner (with P. L. Nimis & M. Tretiach), det. J. Hafellner, GZU (JH87242). • Torino, mountains NE of Bardonecchia, western slopes of Punta Sommeiller, S below Colle de Sommeiller, 45°07'35"N, 6°50'15"E, 2850 m, on plant remnants, 28 July 2001, leg. J. Hafellner (with P. L. Nimis & M. Tretiach), det. J. Hafellner, GZU (JH87433).
An arctic-alpine species, mainly found on mosses and plant debris, often on leaves of *Saxifraga* sp., in *Carex firma*-stands over calcareous substrata; common in the Alps, rare in the Central Apennines.

Peltigera extenuata (Vain.) Lojka (Peltigeraceae)
Emilia-Romagna • Modena, Alto Appennino Modenese Natural Park, Mt. Cimone (summit area), 44°11'40"N, 10°41'54"E, 2137 m, on soil, 4 August 2025, leg. C. Pistocchi, S. Girlanda & F. Zinato, det. S. Girlanda & C. Pistocchi, Hb. Nascimbene (10093). • Reggio Emilia, Appennino Tosco-Emiliano Ntl Park, below Mt. Cusna, 44°17'29"N, 10°23'39"E, 1954 m, on soil, 6 August 2025, leg. C. Pistocchi, S. Girlanda & F. Zinato, det. C. Pistocchi, Hb. Nascimbene (10159).
This species has more or less the same ecology of *P. didactyla*, but seems to be restricted to the montane belt, and differs in the flocculent rhizines and in the C+ pink reaction of medulla and soralia; in Italy there are only a few records from the Alps, but the species could be more widespread, because it was not distinguished from *P. didactyla* in the past. These are the first records for the Apennines.

Peltigera hymenina (Ach.) Delise (Peltigeraceae)
Trentino-Alto Adige • Trento, Stelvio Ntl Park, Frattasecca Integral Reserve, above Malga Frattasecca, 46°21'11"N, 10°38'34"E, 1680 m, on terricolous bryophytes, 2 October 2024, leg. C. Pistocchi, det. C. Pistocchi & J. Nascimbene, Hb. Nascimbene (9524).

On mineral soil in open, but never fully sun-exposed habitats, often associated with mosses, with optimum in the montane belt, from the Alps to the mountains of Southern Italy.

Peltigera lepidophora (Vain.) Bitter (Peltigeraceae)
Emilia-Romagna • Reggio Emilia, Appennino Tosco-Emiliano Ntl Park, below Mt. Cusna, 44°17'27''N, 10°23'35''E, 1973 m, on soil, 10 July 2025, leg. C. Pistocchi, S. Girlanda & F. Zinato, det. C. Pistocchi, Hb. Nascimbene (9915). • Modena, Alto Appennino Modenese Natural Park, below Mt. Cimone, above "Il Casotto" of the Aeronautica Militare, 44°11'46''N, 10°42'16''E, 1952 m, on soil, 5 August 2025, leg. C. Pistocchi, S. Girlanda & F. Zinato, det. C. Pistocchi, Hb. Nascimbene (10110). • Reggio Emilia, Appennino Tosco-Emiliano Ntl Park, between Mt. Cusna and Ricovero Rio Grande, 44°17'37''N, 10°23'48''E, 1877 m, on soil, 6 August 2025, leg. C. Pistocchi, S. Girlanda & F. Zinato, det. S. Girlanda & C. Pistocchi, Hb. Nascimbene (10163).
A mainly boreal-montane, circumpolar pioneer species of base-rich mineral soil, most frequent in upland areas with optimum near tree line; most frequent in the Alps, but apparently reaching the mountains of Sicily.

Peltigera malacea (Ach.) Funck (Peltigeraceae)
Emilia-Romagna • Bologna, Corno alle Scale Natural Park, below Punta Sofia, 44°07'27''N, 10°49'39''E, 1850 m, on soil, 22 October 2021, leg. et det. J. Nascimbene, Hb. Nascimbene (7822). • Modena, Alto Appennino Modenese Natural Park, Mt. Cimone between Salto della Capra and Rifugio Pian Cavallaro, 44°12'04''N, 10°42'36''E, 1820 m, on soil, 21 October 2022, leg. G. Gheza, det. C. Fagiani & G. Gheza, Hb. Nascimbene (8823). • Reggio Emilia, Appennino Tosco-Emiliano Ntl Park, between Lago della Bargetana and Sella del Mt. Prado, 44°15'17''N, 10°24'04''E, 1815 m, on soil, 11 July 2025, leg. C. Pistocchi, S. Girlanda & F. Zinato, det. C. Pistocchi & S. Girlanda, Hb. Nascimbene (10119).
A circumpolar, arctic-alpine lichen found in grasslands and scrublands near and above tree line, often amongst mosses, on siliceous substrata; most common in the Alps, but also found in the Southern Apennines.

Physcia caesia* var. *rhaetica Frey (Physciaceae)
Piemonte • Cuneo, Marguareis Natural Park, just NW above the village Carnino, 44°08'56''N, 7°43'44''E, 1400 m, on slightly calciferous blue-green schists, 19 July 2000, leg. P. L. Nimis & M. Tretiach (with J. Hafellner), det. P. L. Nimis, TSB (33303).
A morph with orange medulla, most frequent in continental Alpine areas, which is worthy of further study.

Physcia magnussonii Frey (Physciaceae)
Piemonte • Cuneo, Monviso Natural Park, N slopes of Monviso, W above Crissolo just W above Lago Fiorenza (S above Pian del Re), 44°41'50''N, 7°05'35''E, 2130 m, on ophiolitic rocks, 25 July 2021, leg. P. L. Nimis & M. Tretiach (with J. Hafellner), det. P. L. Nimis, TSB (35258).
On steeply inclined surfaces of base-rich siliceous rocks, often starting its development in fissures of the rock.

Physcia tribacia (Ach.) Nyl. (Physciaceae)
Veneto • Verona, Monti Lessini, Erbezzo, 45°40'16''N, 11°00'26''E, 1500 m, on rock, 1986, leg. G. Lazzarin, det. P.L. Nimis, TSB (8420).
A widespread Mediterranean to xeric subtropical lichen found on basic siliceous rocks in sunny situations, often with *Peltula euploca* and ecologically related species, but less bound to periodical seepage of water; fairly common in parts of Mediterranean Italy, wherever suitable substrata are available, rarer in dry-warm Alpine valleys.

Physconia muscigena (Ach.) Poelt **var.** ***muscigena*** (Physciaceae)
Liguria • Imperia, Alpi Liguri Natural Park, ridge S above Monesi, W of Col Garezzo, 44°02'50''N, 7°46'22''E, 1850 m, on calciferous schists, 21 July 2000, leg. P. L. Nimis & M. Tretiach (with J. Hafellner), det. P. L. Nimis, TSB (33388).

An arctic-alpine, circumpolar lichen found on mosses and plant debris in open situations, such as in grasslands and on mosses growing on isolated calcareous boulders, from the subalpine and alpine belts of the Alps, throughout the Apennines to the mountains of Sicily.

Physconia muscigena* var. *bayeri (Nádv.) Poelt (Physciaceae)
Trentino-Alto Adige • Trento, Adamello-Brenta Natural Park, below Sasso Rosso along Sentiero Costanzi, 46°16’39”N, 10°54’26”E, 2520 m, on carbonatic soil, 28 July 2022, leg. et det. C. Pistocchi, Hb. Nascimbene (8308).
On terricolous mosses and plant debris in alpine-subalpine situations, rare. According to (Starosta and Svoboda 2020) it is genetically undistinguishable from the typical variety.

Physconia petraea (Poelt) Vězda & Poelt (Physciaceae)
Piemonte • Cuneo, Valle Varaita, ca. 4 km W of the village Sampeyre, slopes exposed to the S just W of Villar, 44°34’55”N, 7°08’05”E, 1120 m, on siliceous base-rich rocks, 25 July 2000, leg. P. L. Nimis & M. Tretiach (with J. Hafellner), det. P. L. Nimis, TSB (32672).
On base-rich siliceous rocks and epilithic mosses in dry-warm situations; known only from southern Europe, and in need of further study.

Placidiopsis pseudocinerea Breuss (Verrucariaceae)
Piemonte • Cuneo, ridge NW above Crissolo N above Pian di Melzè, S-exposed slopes, 44°42’15”N, 7°07’05”E, 1880 m, on calciferous schists, 25 July 2001, leg. P. L. Nimis & M. Tretiach (with J. Hafellner), det. P. L. Nimis, TSB (35345).
An arctic-alpine, circumpolar lichen found on soil and on dying bryophytes on siliceous, base-rich or slightly calciferous soil (e.g. on calcareous schist), with optimum near and above tree line; it can be easily confused with *Catapyrenium cinereum* and is certainly much more widespread through the Italian Alps, also occurring in the Central Apennines.

Polyozosia invadens (H. Magn.) S.Y. Kondr., Lőkös & Farkas (Lecanoraceae)
Veneto • Belluno, Tre Cime di Lavaredo, near Rifugio Auronzo, 46°36’47”N, 12°17’46”E, 2300 m, on dolomitic rocks, 31 September 1994, leg. et det. P. L. Nimis, TSB (19712***)***.
On calciferous rocks in upland areas, often starting the life-cycle on other crustose lichens; certainly more widespread throughout the country. The species is closely related to *P. semipallida*.

Polyozosia perpruinosa Fröberg ex S.Y. Kondr., Lőkös & Farkas (Lecanoraceae)
Veneto • Belluno, Dolomiti Bellunesi Ntl Park, Mt. Pelf, 46°13’50”N, 12°12’09”E, 2374 m, on carbonatic rocks, 28 July 2023, leg. et det. J. Nascimbene, Hb. Nascimbene (8570). • Belluno, Dolomiti Bellunesi Ntl Park, Cima de Zità Sud, 46°16’16”N, 12°09’24”E, 2540 m, on carbonatic rocks, 2 July 2024, leg. et det. J. Nascimbene, Hb. Nascimbene (9419). • Belluno, Pordoi, between Rifugio Vièl dal Pan and Rifugio Fredarola, 46°28’36”N, 11°49’19”E, 2458 m, on volcanic rocks, 8 August 2024, leg. et det. J. Nascimbene, Hb. Nascimbene (9492).
Piemonte • Cuneo, Alpi Liguri Natural Park, N ridge of M Saccarello W above Monesi near Monumento al Redentore, 44°03’45”N, 7°42’50”E, 2200 m, on siliceous base-rich rocks, 18 July 2000, leg. P. L. Nimis & M. Tretiach (with J. Hafellner), det. P. L. Nimis, TSB (33712).
Liguria • Imperia, Alpi Liguri Natural Park, ridge S above Monesi, W of Col Garezzo, 44°02’50”N, 7°46’22”E, 1850 m, on calciferous schists, 21 July 2000, leg. P. L. Nimis & M. Tretiach (with J. Hafellner), det. P. L. Nimis, TSB (33401).
On calciferous rocks, often starting the life-cycle on other crustose lichens, widespread throughout the Alps and probably also along the Apennines.

Polyozosia semipallida (H. Magn.) S.Y. Kondr., Lőkös & Farkas (Lecanoraceae)

Liguria • Imperia, Alpi Liguri Natural Park, just E below Upega on right bank of the creek Negrone (Corvo), 44°07’35”N, 7°44’10”E, 1260 m, on calciferous rocks, 19 July 2000, leg. P. L. Nimis & M. Tretiach (with J. Hafellner), det. P. L. Nimis, TSB (33647).
A calcicolous species found on the top of exposed boulders, in sites often visited by birds; certainly widespread throughout the country, absent from large settlements and very rare on man-made substrata.

Porina ahlesiana (Körb.) Zahlbr. (Porinaceae)
Piemonte • Cuneo, Colle di Sampeyre just W of the Pass, 44°33’10”N, 7°06’55”E, 2300 m, on siliceous rocks, 26 July 2000, leg. P. L. Nimis & M. Tretiach (with J. Hafellner), det. P. L. Nimis, TSB (32846).
A rare species of shaded-humid siliceous rocks, with a somehow western distribution in Europe and a wide altitudinal range.

Porpidia trullisata (Kremp.) Körb. (Lecideaceae)
Veneto • Belluno, Dolomiti Bellunesi Ntl Park, Pezedei, below Forcella del Zità Sud, 46°16’27”N, 12°09’13”E, 2285 m, on selciferous carbonatic rocks, 2 July 2024, leg. et det. J. Nascimbene, Hb. Nascimbene (9450). • Belluno, Dolomiti Bellunesi Ntl Park, Vette Feltrine near Passo Vette Grandi, 46°05’27”N, 11°50’34”E, 2050 m, on selciferous carbonatic rocks, 5 August 2024, leg. et det. J. Nascimbene, Hb. Nascimbene (9270).
On north-facing, steeply inclined surfaces of weakly calciferous rocks with optimum above tree line, reaching the nival belt; apparently restricted to the Alps in Italy.

Porpidia zeoroides (Anzi) Knoph & Hertel (Lecideaceae)
Veneto • Belluno, Pordoi, between Rifugio Vièl dal Pan and Rifugio Fredarola, 46°28’35”N, 11°49’29”E, 2492 m, on volcanic rocks, 8 August 2024, leg. et det. J. Nascimbene, Hb. Nascimbene (9489).
On steeply inclined, often north-exposed faces of more or less calciferous rocks in upland areas, reaching beyond tree line in the Alps.

Protoblastenia aurata Poelt & Vězda (Psoraceae)
Veneto • Belluno, Dolomiti Bellunesi Ntl Park, Vette Feltrine near Passo Vette Grandi, 46°05’27”N, 11°50’34”E, 2050 m, on selciferous carbonatic rocks, 5 August 2024, leg. et det. J. Nascimbene, Hb. Nascimbene (9271).
On calciferous siliceous rocks, especially schists, near and above tree line.

Protoblastenia siebenhaariana (Körb.) J. Steiner **var. *siebenhaariana*** (Psoraceae)
Piemonte • Cuneo, cirque E below summit of Rocca dell’Abisso W of Col di Tenda NW below Fort de Giaure, 44°08’25”N, 7°31’05”E, 2250 m, on gneiss, 22 July 2000, leg. P. L. Nimis & M. Tretiach (with J. Hafellner), det. P. L. Nimis, TSB (34497*)*.
A mainly arctic-alpine, probably circumpolar lichen found on base-rich or calciferous siliceous rocks and on dolomite in upland areas; not frequent in Italy, but locally common in the Alps.

Protocandelariella subdeflexa (Nyl.) Poelt ex D. Liu, Hur & S.Y. Kondr. (Candelariaceae)
Piemonte • Cuneo, near the village of Upega, 44°07’45”N, 7°43’40”E, 1300 m, on bark of *Juglans* sp., 19 July 2000, leg. P. L. Nimis & M. Tretiach (with J. Hafellner), det. P. L. Nimis, TSB (33596).
A mild-temperate, perhaps holarctic lichen found on isolated trees with base-rich bark, especially *Fraxinus, Populus* and *Juglans*, often near the base of the trunks, probably declining.

Protoparmelia badia* var. *cinereobadia (Harm.) Hafellner & Türk (Parmeliaceae)
Veneto • Belluno, Dolomiti Bellunesi Ntl Park, North side of Mt. Talvena, 46°16’03”N, 12°09’21”E, 2437 m, on selciferous carbonatic rocks, 24 June 2025, leg. et det. J. Nascimbene, Hb. Nascimbene (9990).
This name has been used for any morph of *P. badia* with an ash-grey to whitish thallus, but its application was probably not consistent with the original concept (grey morphs with a thick, rough thallus, medulla reacting

KC+ fleeting pink, and apothecia mostly smaller than 1 mm in diam.). According to Roux and coll. (2014) this variety occurs on weakly to moderately calciferous siliceous rocks from the montane to the nival belt.

Protoparmeliopsis muralis* var. *subcartilaginea (Poelt) Dose & Nimis (Lecanoraceae)
Piemonte • Cuneo, Monviso Natural Park, N slopes of Monviso, W above Crissolo just W above Lago Fiorenza (S above Pian del Re), 44°41'50"N, 7°05'35"E, 2130 m, on ophiolitic rocks, 25 July 2021, leg. P. L. Nimis & M. Tretiach (with J. Hafellner), det. P. L. Nimis, TSB (35236).
On horizontal to weakly inclined surfaces of siliceous rocks in upland areas. A poorly known taxon, well worthy of further study.

Protothelenella corrosa (Körb.) H. Mayrhofer & Poelt (Protothelenellaceae)
Lombardia • Brescia, Adamello Natural Park, Val Paghera S of Vezza d'Oglio, by the trail from the uppermost parking area to Rifugio Aviolo, 46°12'00"N, 10°24'55"E, 1850 m, on granitic boulders, 25 July 2006, leg. J. Hafellner (with J. Nascimbene, L. Muggia & M. Tretiach), det. J. Hafellner, GZU (JH88549).
This is the only saxicolous species of the genus. It mostly grows on various types of siliceous rocks (quartzite, granite, gneiss, schists, sandstone, vulcanites, including such with low content of calcium) at sites with permanent high humidity (but not submersed). It is widespread in the Holarctic region. with most records from Europe, from the Arctic tree line to the nemoral zone. In the Central European mountains, it has the optimum from the montane to the lower alpine belt. In Italy still undercollected.

Protothelenella sphinctrinoidella (Nyl.) H. Mayrhofer Poelt (Protothelenellaceae)
Piemonte • Cuneo, Colle della Lombarda, slopes exposed to the W just NE below the pass, 44°12'20"N, 7°09'10"E, 2350 m, on gneiss, 24 July 2000, leg. P. L. Nimis & M. Tretiach (with J. Hafellner), det. P. L. Nimis, TSB (34303).
On acid to subneutral soil, dying bryophytes and lichens, more rarely on decaying plants in upland areas, often in rather disturbed habitats, e.g. along mountain track sides; certainly more widespread in the Alps, but probably overlooked.

Protothelenella sphinctrinoides (Nyl.) H. Mayrhofer & Poelt (Protothelenellaceae)
Veneto • Belluno, Pordoi, between Rifugio Vièl dal Pan and Rifugio Fredarola, 46°28'36"N, 11°49'19"E, 2458 m, on plant debris, 8 August 2024, leg. et det. J. Nascimbene, Hb. Nascimbene (9368).
An arctic-alpine to boreal-montane, circumpolar lichen found on dying bryophytes on soil and rock, more rarely directly on soil, in rather disturbed sites (e.g., on mountain track sides) with a long snow-lie, often in crevices or small depressions of the ground; much undercollected and probably ranging throughout the Alps, with optimum above tree line.

Pseudephebe minuscula (Arnold) Brodo & D. Hawksw. (Parmeliaceae)
Piemonte • Cuneo, Rocca dell'Abisso W of Col di Tenda just N below the summit, 44°08'37"N, 7°30'14"E, 2750 m, on gneiss, 22 July 2000, leg. P.L. Nimis & M. Tretiach (with J. Hafellner), det. P.L. Nimis, TSB (34483). • Cuneo, Alpi Marittime, Rocca dell'Abisso W of Colle di Tenda, E below summit, steep slopes towards uppermost Vallone dell'Abisso, 44°08'37"N, 7°30'29"E, 2630 m, on steep rock faces (gneiss), 22 July 2000, leg. J. Hafellner (with P. L. Nimis & M. Tretiach), det. J. Hafellner, GZU (JH87381). • Torino, mountains NE of Bardonecchia, western slopes of Punta Sommeiller, S below Colle de Sommeiller, 45°07'35"N, 6°50'15"E, 2850 m, on boulders of siliceous schist, 28 July 2001, leg. J. Hafellner (with P. L. Nimis & M. Tretiach), det. J. Hafellner, GZU (JH87417).
Valle d'Aosta • Aosta, Gran Paradiso Ntl Park, near the trail from Pont to the Rifugio Vittorio Emanuele II, 45°30'50"N, 7°13'17"E, 2550 m, on vertical rock faces (gneiss), 31 July 2001, leg. J. Hafellner (with P. L. Nimis & M. Tretiach), det. J. Hafellner, GZU (JH87028). • Aosta, Colle del Gran San Bernardo, SW below the pass, 45°52'18"N, 7°09'34"E, 2500 m, on vertical siliceous rock faces, 1 August 2001, leg. J. Hafellner (with P. L. Nimis & M. Tretiach), det. J. Hafellner, GZU (JH87138).

An arctic-alpine, circumpolar species found on hard siliceous rocks (including pure quartz) in wind-exposed situations near and especially above tree line, up to the nival belt of the Alps; often confused with *P. pubescens* in the older literature; most common in the Alps, rarer in the high Mediterranean mountains, reaching south to the mountains of Calabria.

Pseudephebe pubescens (L.) M. Choisy (Parmeliaceae)
Veneto • Belluno, Arabba, below Forcella Porta Vescovo, 46°28’35’’N, 11°52’08’’E, 2380 m, on volcanic rocks, 1 August 2025, leg. et det. J. Nascimbene, Hb. Nascimbene (10015).
Liguria • Imperia, Alpi Liguri Natural Park, mountain ridge N above the village Triora, W below Col Garezzo, on slopes exposed to the S, 44°02’50’’N, 7°45’15’’E, 1650 m, on inclined faces of sandstone boulders, 21 July 2000, leg. J. Hafellner (with P. L. Nimis & M. Tretiach), det. J. Hafellner, GZU (JH87326).
An arctic-alpine to (more rarely) boreal-montane lichen which is ecologically similar to *P. minuscula*, but with a somehow broader altitudinal range; more frequent than *P. minuscula* in the mountains of Southern Italy.

Pseudevernia furfuracea* var. *ceratea (Ach.) D. Hawksw. (Parmeliaceae)
Emilia-Romagna • Modena, Alto Appennino Modenese Natural Park, Lago della Ninfa, 44°12’42’’N, 10°43’30’’E, 1500 m, on bark of *Larix decidua*, 21 October 2022, leg. et det. J. Nascimbene, Hb. Nascimbene (8020).
Distinguished by the presence of olivetoric acid, this chemical strain is more frequent than *Pseudevernia furfuracea* (L.) Zopf var. *furfuracea* in dry-continental areas (Martellos 2003) and at higher elevations (Cecconi et al. 2025).

Psora vallesiaca (Schaer.) Timdal (Psoraceae)
Trentino-Alto Adige • Trento, Adamello-Brenta Natural Park, Valagola between Lago di Valagola and Doss del Sabion, 46°09’53’’N, 10°48’52’’E, 1945 m, on marly limestone, 5 September 2022, leg. J. Nascimbene & C. Pistocchi, det. C. Pistocchi, Hb. Nascimbene (8974).
On calciferous rocks and thin layers of soil in rock fissures, not rare where suitable habitats are present.

Psoroglaena biatorella (Arnold) Lücking & Sérus. (Verrucariaceae)
Veneto • Belluno, Pordoi, between Rifugio Vièl dal Pan and Rifugio Fredarola, 46°28’36’’N, 11°49’19’’E, 2458 m, on plant debris, 8 August 2024, leg. et det. J. Nascimbene, Hb. Nascimbene (9493).
Trentino-Alto Adige • Bolzano, Puez-Odle Natural Park, Mt. Seceda (Geisler Spitzen) NE of Ortisei (St. Ulrich), on the ridge just W above Forc Pana (Pana Scharte), 46°36’05’’N, 11°44’05’’E, 2500 m, on soil in crevices, 2 September 2002, leg. J. Hafellner GZU (JH61241).
An inconspicuous lichen found on thin layers of more or less calciferous, humus-rich ground, or over epilithic mosses, mostly in upland areas; probably overlooked and more widespread, but never common, in the Alps.

Psoroma tenue Henssen **var. *boreale*** Henssen (Pannariaceae)
Emilia-Romagna • Reggio Emilia, Appennino Tosco-Emiliano Ntl Park, Mt. Cusna, 44°17’18’’N, 10°23’30’’E, 2098 m, on soil, 10 July 2025, leg. C. Pistocchi, S. Girlanda & F. Zinato, det. C. Pistocchi & J. Nascimbene, Hb. Nascimbene (9944). • Reggio Emilia, Appennino Tosco-Emiliano Ntl Park, Mt. Prado (summit area), 44°14’59’’N, 10°24’22’’E, 2037 m, on soil, 12 July 2025, leg. C. Pistocchi, S. Girlanda & F. Zinato, det. S. Girlanda & C. Pistocchi, Hb. Nascimbene (10138). • Modena, Alto Appennino Modenese Natural Park, Mt. Cimone along the “Sentiero dell’Atmosfera”, 44°11’47’’N, 10°41’44’’E, 2018 m, on soil, 4 August 2025, leg. C. Pistocchi, S. Girlanda & F. Zinato, det. C. Pistocchi, Hb. Nascimbene (10105). • Modena, Alto Appennino Modenese Natural Park, below Mt. Cimone, above “Il Casotto” of the Aeronautica Militare, 44°11’46’’N, 10°42’17’’E, 1914 m, on soil, 5 August 2025, leg. C. Pistocchi, S. Girlanda & F. Zinato, det. S. Girlanda & C. Pistocchi, Hb. Nascimbene (10113).
An arctic-alpine, circumpolar lichen weak in competition, found on wet, naked soil, near glaciers or late snow-beds over siliceous substrata; certainly more widespread in the nival belt of the Alps, but easily confused with

P. hypnorum (Jørgensen 2004, Breuss 2012), though rather easily recognized by the shiny brownish colour and the flat apothecia. These are the first records from the Apennines.

Psoronactis dilleniana (Ach.) Ertz & Tehler (Roccellaceae)
Piemonte • Cuneo, Colle di Sampeyre just W of the Pass, 44°33'10"N, 7°06'55"E, 2300 m, on siliceous rocks, 26 July 2000, leg. P. L. Nimis & M. Tretiach (with J. Hafellner), det. P. L. Nimis, TSB (32833).
On hard crystalline rocks, beneath overhangs and in crevices which are seldom wetted by rain, mostly in upland areas.

Pyrenodesmia chalybaea (Fr.) A. Massal. (Teloschistaceae)
Piemonte • Cuneo, western ridge of Mt. Nebin ca. 1 Km E of Colle di Sampeyre, 44°32'40"N, 7°08'20"E, 2380 m, on calcareous rocks, 26 July 2000, leg. P. L. Nimis & M. Tretiach (with J. Hafellner), det. P. L. Nimis, TSB (32936).
A mild-temperate species known from Europe and adjoining Africa and Asia, found on hard calciferous rocks (mostly on compact limestone) and dolomite, often, but not exclusively, on steeply inclined faces; especially common in Central and Southern Italy, above the Mediterranean belt.

Pyrenodesmia variabilis (Pers.) A. Massal. (Teloschistaceae)
Valle d'Aosta • Aosta, Passo del Piccolo San Bernardo, 45°41'47"N, 6°53'55"E, 2050 m, on calciferous rocks, September 1997, leg. et det. P. L. Nimis, TSB (29427).
A probably holarctic, subtropical to boreal-montane, very polymorphic lichen found a wide variety of calciferous substrata wetted by rain, with a wide altitudinal range. The whole complex is worthy of further study.

Pyrenula laevigata (Pers.) Arnold (Pyrenulaceae)
Friuli Venezia Giulia • Udine, Passo di Monte Croce Carnico, 46°35'40"N, 12°58'01"E, 1300 m, on bark of *Acer* sp., 28 January 1982, leg. et det. P. L. Nimis, TSB (2892).
A temperate species of smooth bark, most frequent on *Carpinus* and *Fagus* in open, humid woodlands.

Ramalina arsenii Sérus., van den Boom & Magain (Ramalinaceae)
Trentino-Alto Adige • Trento, Stelvio Ntl Park, Rabbi, Somrabbi, 46°24'49"N, 10°47'22"E, 1462 m, on siliceous rocks, 19 June 2025, leg. C. Pistocchi, det. G. Gheza & C. Pistocchi, Hb. Nascimbene (10198). • Trento, Stelvio Ntl Park, Val del Monte, Pradacci, 46°47'22"N, 10°37'45"E, 1642 m, on siliceous rocks, 27 August 2025, leg. et det. C. Pistocchi, Hb. Nascimbene (10355).
A recently-described species, apparently widespread in the Alps and also known from the Pyrenees, mostly growing beneath overhangs of weakly calciferous rocks at low elevations, more rarely on bark. The species is likely to be more frequent in Italy, because in the past it might have been identified as *R. pollinaria*.

Ramonia melathelia (Nyl.) Ertz (Gyalectaceae)
Piemonte • Cuneo, western ridge of Mt. Nebin ca. 1 Km E of Colle di Sampeyre, 44°32'40"N, 7°08'20"E, 2380 m, on soil and plant debris, 26 July 2000, leg. P. L. Nimis & M. Tretiach (with J. Hafellner), det. P. L. Nimis, TSB (32915). • Cuneo, *ibidem*, 26 July 2000, leg. J. Hafellner (with P. L. Nimis & M. Tretiach), det. J. Hafellner, GZU (JH75632).
Liguria • Imperia, Alpi Liguri Natural Park, mountain ridge S above the village Monesi, on the ridge W above Col Garezzo, 44°02'50"N, 7°46'22"E, 1850 m, on plant remnants and bryophytes, 21 July 2000, leg. J. Hafellner (with P. L. Nimis & M. Tretiach), det. J. Hafellner, GZU (JH87260).
An arctic-alpine, circumpolar species found on dying bryophytes, humus-rich soil and plant remains over more or less calcareous substrata near or above tree line.

Rehmia furfurosa (H. Magn. & Poelt) E.J. Möller, Timdal, Haugan & Bendiksby (Rhizocarpaceae)

Piemonte • Cuneo, Vallone di S. Anna SW above Vinadio by the road to Colle della Lombarda, 44°16’25”N, 7°08’00”E, 1340 m, on iron-rich siliceous rocks, 24 July 2000, leg. P. L. Nimis & M. Tretiach (with J. Hafellner), det. P. L. Nimis, TSB (33843).
On metal-rich siliceous rocks, mostly on steeply inclined to rain-sheltered faces in upland areas; easy to overlook, being mostly sterile. This is the first record from the Italian Alps, where the species should be looked for further.

Rhizocarpon ferax H. Magn. (Rhizocarpaceae)
Veneto • Belluno, Arabba, N-facing slope of Porta Vescovo, start of the via ferrata, 46°28’24”N, 11°52’24”E, 2588 m, on volcanic rocks, 1 August 2025, leg. et det. J. Nascimbene, Hb. Nascimbene (10302).
On siliceous rocks near and above treeline, starting the life-cycle on other crustose lichens. Already reported in Italy from Trentino-Alto Adige (Nascimbene 2005), it is probably more widespread in the Alps.

Rhizocarpon geographicum* subsp. *diabasicum (Räsänen) Poelt & Vězda (Rhizocarpaceae)
Veneto • Belluno, Pordoi, between Rifugio Vièl dal Pan and Rifugio Fredarola, 46°28’34”N, 11°49’42”E, 2552 m, on volcanic rocks, 8 August 2024, leg. et det. J. Nascimbene, Hb. Nascimbene (9487).
On siliceous rocks, sometimes also on superficially decalcified calcareous rocks, where it appears in forms with a whitish thallus, near and above tree line.

Rhizocarpon geographicum* subsp. *lindsayanum (Räsänen) Ahti (Rhizocarpaceae)
Trentino-Alto Adige • Trento, Adamello-Brenta Natural Park, between Passo Presena and Lago Scuro, 46°12’59”N, 10°34’34”E, 2838 m, on tonalite, 9 August 2023, leg. et det. J. Nascimbene, Hb. Nascimbene (8631).
This subspecies seems to have a circumboreal distribution. It is found on siliceous, often dust-impregnated rocks, also on walls and boulders in semi-urban environments. It certainly occurs also in the Alps, but was rarely distinguished from *Rh. geographicum* s. lat.

Rhizocarpon macrosporum Räsänen (Rhizocarpaceae)
Veneto • Belluno, Pordoi, between Rifugio Vièl dal Pan and Rifugio Fredarola, 46°28’35”N, 11°49’29”E, 2492 m, on volcanic rocks, 8 August 2024, leg. J. Nascimbene, det. H. Mayrhofer, Hb. Nascimbene (9363).
A chemically heterogeneous species of dust-impregnated, exposed siliceous rocks, including walls in small settlements, most common in upland areas.

Rhizocarpon norvegicum Räsänen (Rhizocarpaceae)
Veneto • Belluno, Pordoi, between Rifugio Vièl dal Pan and Rifugio Fredarola, 46°28’34”N, 11°49’42”E, 2552 m, on volcanic rocks, 8 August 2024, leg. et det. J. Nascimbene, Hb. Nascimbene (9486).
A pioneer species of schistose, slightly calciferous or basic eruptive rocks in upland areas, which often starts the life-cycle on members of Acarosporaceae.

Rhizocarpon ridescens (Nyl.) Zahlbr. (Rhizocarpaceae)
Piemonte • Cuneo, Vallone di S. Anna SW above Vinadio by the road to Colle della Lombarda, 44°16’25”N, 7°08’00”E, 1340 m, on iron-rich siliceous rocks, 24 July 2000, leg. P. L. Nimis & M. Tretiach (with J. Hafellner), det. P. L. Nimis, TSB (33849). • Cuneo, *ibidem*, 24 July 2000, leg. J. Hafellner (with P. L. Nimis & M. Tretiach), det. J. Hafellner, GZU (JH87703).
Valle d’Aosta • Aosta, Val Veny W of Courmayeur, moraines E of Lago di Combal, 45°46’35”N, 6°52’10”E, 1980 m, on erratic boulder of iron-rich schist, 30 July 2001, leg. J. Hafellner (with P. L. Nimis & M. Tretiach), det. J. Hafellner, GZU (JH88598). • Aosta, Gran Paradiso Ntl Park, Val Savarenche, between the villages Valsavarenche and Pont, 45°33’25”N, 7°12’40”E, 1740 m, on inclined rock faces of iron-rich rock lenses in large boulders of siliceous schist**,** 31 July 2001, leg. J. Hafellner (with P. L. Nimis & M. Tretiach), det. J. Hafellner, GZU (JH87173).

On iron-rich siliceous rocks, mostly under overhangs, near or above tree line; easily overlooked, being always sterile.

Rhizoplaca chrysoleuca (Sm.) Zopf (Lecanoraceae)
Veneto • Belluno, Arabba, below Forcella Porta Vescovo, 46°28’35”N, 11°52’08”E, 2380 m, on volcanic rocks, 1 August 2025, leg. et det. J. Nascimbene, Hb. Nascimbene (10016).
A widespread holarctic lichen found on bird's perching siliceous rocks and boulders, especially in upland areas; most frequent in areas with a dry-subcontinental climate, e.g., in the central Alps, but also occurring on the high Mediterranean mountains.

Rimularia gibbosa (Sm.) Zopf (Trapeliaceae)
Lombardia • Brescia, Adamello Natural Park, Val Paghera S of Vezza d’Oglio, by the trail from the uppermost parking area to Rifugio Aviolo, 46°12’00”N, 10°24’55”E, 1850 m, on vertical rock faces of granitic outcrops, 25 July 2006, leg. J. Hafellner (with J. Nascimbene, L. Muggia, M. Tretiach), det. J. Hafellner, GZU (JH88446). • Brescia, surroundings of Lago d’Aviolo c. 5.5 km S above Vezza d’Oglio, by the trail S of Rifugio Aviolo, E above the lake, 46°11’40”N, 10°24’55”E, 1940 m, on vertical rock faces of granitic boulders, 25 July 2006, leg. J. Hafellner (with J. Nascimbene, L. Muggia & M. Tretiach), det. J. Hafellner, GZU (JH88602) stored under *Schaereria cinereorufa*. • Bergamo, Orobie Bergamasche Natural Park, between Baita Brandelegno and Baita Napoleù, 46°00’34”N, 10°05’38”E, 1522 m, on siliceous sandstone, 17 August 2022, leg. G. Gheza, det. H. Mayrhofer, Hb. Gheza (22081731). • Bergamo, Orobie Bergamasche Natural Park, below Rifugio Ca' Rossa, 46°00’32”N, 10°02’09”E, 1580 m, on iron-rich rocks, 16 February 2023, leg. G. Gheza, det. H. Mayrhofer, Hb. Gheza.
Valle d’Aosta • Aosta, Val Veny W of Courmayeur, moraines E of Lago di Combal, 45°46’35”N, 6°52’10”E, 1980 m, on erratic boulder of iron-rich schist, 30 July 2001, leg. J. Hafellner (with P. L. Nimis & M. Tretiach), det. J. Hafellner, GZU (JH88599). • Aosta, Gran Paradiso Ntl Park, near the trail from Pont to the Rifugio Vittorio Emanuele II, 45°30’50”N, 7°13’17”E, 2550 m, on vertical rock faces (gneiss), 31 July 2001, leg. J. Hafellner (with P. L. Nimis & M. Tretiach), det. J. Hafellner, GZU (JH87027).
The species is found in nature in two morphs, one more or less richly fruiting the other sterile, developing asexual diaspores. It grows on various types of siliceous rocks, mostly on subvertical rock faces (small sterile thalli often on base faces of horizontal crevices). The overall holarctic species is widespread in Europe, including the Alps from the montane to high alpine belt (Nimis et al. 2018). Its distribution in Italy is still incompletely documented.

Rinodina calcarea (Arnold) Arnold (Physciaceae)
Liguria • Imperia, Alpi Liguri Natural Park, ridge S above Monesi, W of Col Garezzo, 44°02’50”N, 7°46’22”E, 1850 m, on calciferous schists, 21 July 2000, leg. P. L. Nimis & M. Tretiach (with J. Hafellner), det. P. L. Nimis, TSB (33400*)*.
A southern species found on the top of sun-exposed boulders of dolomite, limestone and calcareous schists, with a rather wide altitudinal range.

Rinodina cana (Arnold) Arnold (Physciaceae)
Piemonte • Cuneo, Rocca dell’Abisso W of Col di Tenda just N below the summit, 44°08’37”N, 7°30’14”E, 2750 m, on gneiss, 22 July 2000, leg. P. L. Nimis & M. Tretiach (with J. Hafellner), det. P. L. Nimis, TSB (34548).
On steeply inclined surfaces of weakly calcareous schists and basic siliceous rocks, most frequent in dry-warm areas; certainly overlooked and more frequent, at least in the Alps.

Rinodina candidogrisea Hafellner, Muggia & Obermayer (Physciaceae)
Veneto • Belluno, Dolomiti Bellunesi Ntl Park, North side of Mt. Talvena, 46°16’01”N, 12°09’21”E, 2460 m, on plant debris, 24 June 2025, leg. et det. J. Nascimbene, Hb. Nascimbene (10055).

A recently-described, ornithocoprophilous, terricolous species growing on mosses and plant debris over calcareous substrata near and above tree line; probably widespread throughout the Alps.

Rinodina castanomelodes H. Mayrhofer & Poelt (Physciaceae)
Piemonte • Cuneo, ridge NW above Crissolo N above Pian di Melzè, S-exposed slopes, 44°42'15"N, 7°07'05"E, 1880 m, on calciferous schists, 25 July 2001, leg. P. L. Nimis & M. Tretiach (with J. Hafellner), det. P. L. Nimis, TSB (35325).
An arctic-alpine to boreal-montane, perhaps circumpolar lichen found on limestone, marl and calcareous schists in and above tree line; widespread but not common in the Alps, where it can reach the nival belt.

Rinodina cinnamomea (Th. Fr.) Räsänen (Physciaceae)
Emilia-Romagna • Reggio Emilia, Appennino Tosco-Emiliano Ntl Park, below Il Passone, 44°16'06"N, 10°25'7"E, on plant debris, 8 July 2025, leg. C. Pistocchi, S. Girlanda & F. Zinato, det. C. Pistocchi, Hb. Nascimbene (10155). • Reggio Emilia, Appennino Tosco-Emiliano Ntl Park, below Mt. Cusna, 44°17'19"N, 10°23'27"E, 2075 m, on soil, 10 July 2025, leg. C. Pistocchi, S. Girlanda & F. Zinato, det. C. Pistocchi, Hb. Nascimbene (9936). • Modena, Alto Appennino Modenese Natural Park, NW-crest below Mt. Cimone, 44°11'42"N, 10°41'52"E, 2097 m, on soil, 4 August 2025, leg. C. Pistocchi, S. Girlanda & F. Zinato, det. C. Pistocchi, Hb. Nascimbene (10100).
On soil, mosses, and plant debris in tundra-like environments over more or less calciferous substrata, with optimum near tree line. These are the first records from the Apennines.

Rinodina dubyana (Hepp) J. Steiner (Physciaceae)
Piemonte • Cuneo, western ridge of Mt. Nebin ca. 1 Km E of Colle di Sampeyre, 44°32'40"N, 7°08'20"E, 2380 m, on calcareous rocks, 26 July 2000, leg. P. L. Nimis & M. Tretiach (with J. Hafellner), det. P. L. Nimis, TSB (32930).
A mainly temperate species found on steeply inclined to rain-sheltered, sunny surfaces of limestone and dolomite wetted by rain, sometimes also on pebbles on the ground, with optimum below the subalpine belt.

Rinodina griseosoralifera Coppins (Physciaceae)
Trentino-Alto Adige • Trento, Adamello-Brenta Natural Park, Val Nambrone near the bridge over Fiume Sarca, 46°12'50"N, 10°45'12"E, 1550 m, on bark of *Salix caprea*, 20 July 2023, leg. J. Nascimbene, det. J. Nascimbene & H. Mayrhofer, Hb. Nascimbene (8654).
Liguria • Imperia, Alpi Liguri Natural Park, just E below Upega on right bank of the creek Negrone (Corvo), 44°07'35"N, 7°44'10"E, 1260 m, on bark of *Juglans* sp., 19 July 2000, leg. P. L. Nimis & M. Tretiach (with J. Hafellner), det. P. L. Nimis, TSB (33566).
A species found on trunks of broad-leaved trees, often near the base, sometimes invading epiphytic mosses; easy to overlook, being mostly sterile, it is probably more widespread throughout the country, mostly at relatively low elevations.

Rinodina guzzinii Jatta (Physciaceae)
Piemonte • Cuneo, Marguareis Natural Park, just NW above the village Carnino, 44°08'56"N, 7°43'44"E, 1400 m, on slightly calciferous blue-green schists, 19 July 2000, leg. P. L. Nimis & M. Tretiach (with J. Hafellner), det. P. L. Nimis, TSB (33332).
An Irano-Turanian-Mediterranean species found on more or less horizontal, exposed surfaces of weakly calciferous rocks, most frequent in dry-warm areas below tree line.

Rinodina notabilis (Lynge) Sheard (Physciaceae)
Veneto • Belluno, Dolomiti Bellunesi Ntl Park, Vette Feltrine, Busa delle Vette, 46°05'55"N, 11°50'27"E, 1900 m, on carbonatic rocks, leg. et det. J. Nascimbene, Hb. Nascimbene (10473).
On more or less calciferous rocks in upland areas; the species is known also from Austria, Switzerland, France, Slovakia, and Spain, and from North America (Sheard 2010).

Rinodina parasitica H. Mayrhofer & Poelt (Physciaceae)
Toscana • Pistoia, surroundings of Abetone, Val di Luce, Alpe Tre Potenze, along the lift route "La Fariola", 44°07'21"N, 10°38'19"E, 1780 m, on slightly calciferous sandstone, 27 October 1978, leg. et det. H. Mayrhofer & J. Poelt, GZU (HM862).
The species is lichenicolous on a sterile whitish crustose lichen occurring on slightly calciferous sandstone. It was erroneously reported as *Rinodina milvina* by Mayrhofer and Poelt (1979). The specimens from other altitudes (1550 m, 1830 m) refer to *Rinodina milvina* as well as the samples distributed as Plantae Graecenses Lichenes 172 (Poelt 1979). This is the first record outside the Alps in Italy.

Rinodina zwackhiana (Kremp.) Körb. (Physciaceae)
Trentino-Alto Adige • Trento, Fiera di Primiero, Chiesa di Santa Maria Assunta, 46°10'36"N, 11°49'44"E, 700 m, on the wall of the church, 1 July 2025, leg. R. Kray, det. M. Westberg, Hb. Kray (1427).
A rarely collected species on man-made substrata. The thallus is superficially similar to *Verrucaria furfuracea* (B. de Lesd.) Breuss and *Verrucaria tectorum* (A. Massal.) Körb. but with lecanorine apothecia. A further diagnostic character is the K+ violet epithecium.

Ropalospora viridis (Tønsberg) Tønsberg (Ropalosporaceae)
Trentino-Alto Adige • Trento, Adamello-Brenta Natural Park, Val Nambrone, near Rifugio Nambrone, 46°12'14"N, 10°45'09"E, 1330 m, on bark of *Abies alba*, 20 July 2023, leg. J. Nascimbene, det. H. Mayrhofer, Hb. Nascimbene (8653).
On the smooth bark of deciduous and coniferous trees in cold-humid, open woodlands; perhaps more widespread, but certainly not common.

Rostania occultata (Bagl.) Otálora, P.M. Jørg. & Wedin (Collemataceae)
Friuli Venezia Giulia • Udine, W of Ampezzo by the road to Passo del Pura, near Albergo e Ristorante Pura, 46°24'50"N, 12°46'00"E, 715 m, on branches of *Sambucus nigra*, 17 August 1994, leg. et det. J. Hafellner, GZU (JH87209).
The specimen reported here has richly fruiting crowded thalline granules. Together with the oblong muriform ascospores this fits the species concept as circumscribed in a recent revision (Košuthová et al. 2022). *R. occultata* settles on base-rich to subneutral bark of various trees and shrubs and is widely distributed in Europe and North America. In the Alps it appears to be the only corticolous species of the genus and is found from the colline to montane belt. In Italy it is widely distributed all over the country but nowhere common.

Rusavskia sorediata (Vain.) S.Y. Kondr. & Kärnefelt (Teloschistaceae)
Liguria • Imperia, Alpi Liguri Natural Park, ridge S above Monesi, W of Col Garezzo, 44°02'50"N, 7°46'22"E, 1850 m, on calciferous schists, 21 July 2000, leg. P. L. Nimis & M. Tretiach (with J. Hafellner), det. P. L. Nimis, TSB (33389).
A mainly arctic-alpine, circumpolar species found on steeply inclined to rain-sheltered, nutrient-enriched surfaces of exposed calcareous or dolomitic boulders, sometimes also of basic siliceous rocks; most frequent in the Alps, where it can reach the nival belt, much rarer in the Apennines.

Sarcogyne platycarpoides Anzi (Acarosporaceae)
Trentino-Alto Adige • Trento, Paneveggio-Pale di San Martino Natural Park, Val Canali, Rio Pradidali, 46°13'29"N, 11°52'07"E, 1330 m, on semi-shaded dolomite, 4 July 2025, leg. et det. R. Kray, Hb. Kray (1394).
A widespread species, known from Austria, Czech Republic, Germany, Italy, Norway, western Russia and Switzerland, most common on dolomitic pebbles above treeline. Probably more widespread in the Alps.

Sarcosagium campestre (Fr.) Poetsch & Schied. (Thelocarpaceae)

Trentino-Alto Adige • Trento, Paneveggio-Pale di San Martino Natural Park, Val Canali, eastern trail of the Rio Pradidali, 46°13’12”N, 11°52’27”E, 1220 m, on soil, 4 July 2025, leg. R. Kray & M. Schultz, det. M. Schultz, Hb. Kray (1378).
An early coloniser of more or less calciferous soil, moribund bryophytes and plant debris, sometimes also growing on decaying wood, mostly in disturbed habitats, e.g. in burned sites, mostly in upland areas; perhaps more widespread in the Alps, and also occurring in the Central Apennines.

Schaereria cinereorufa (Schaer.) Th. Fr. (Schaereriaceae)
Lombardia • Brescia, Adamello Natural Park, surroundings of Lago d’Aviolo c. 5.5 km S above Vezza d’Oglio, by the trail S of Rifugio Aviolo, E above the lake, 46°11’40”N, 10°24’55”E, 1940 m, on vertical rock faces of granitic boulders, 25 July. 2006, leg. J. Hafellner (with J. Nascimbene, L. Muggia & M. Tretiach), det. J. Hafellner, GZU (JH88602).
This altogether rare species mostly grows on various types of regularly wetted siliceous rocks, often on steep to subvertical faces where it can also overgrow small moss cushions and parmelioid macrolichens. The ecological requirements are similar to those of the more common *Lepra corallina* or *Miriquidica leucophaea*. The species has a holarctic distribution, with most records from Europe, from the subarctic to the temperate zone. In the central European mountains, it is usually found from the montane to the lower alpine belt. In Italy so far rarely recorded and probably restricted to the Alps.

Schaereria fuscocinerea (Nyl.) Clauzade & Cl. Roux (Schaereriaceae)
Liguria • Imperia, Alpi Liguri Natural Park, mountains N above Triora on the road to Col Garezzo, 44°02’50”N, 7°45’15”E, 1650 m, on sandstone, 21 July 2000, leg. P. L. Nimis & M. Tretiach (with J. Hafellner), det. P. L. Nimis, TSB (33478). • Imperia, *ibidem*, 21 July 2000, leg. J. Hafellner (with P. L. Nimis & M. Tretiach), det. J. Hafellner, GZU (JH87328).
An arctic-alpine to boreal-montane, circumpolar species found on hard siliceous rocks in exposed situations, with optimum above tree line, reaching the nival belt in the Alps; common only in the Alps, much rarer in the Apennines.

Schismatomma ricasolii (A. Massal.) Egea & Torrente (Roccellaceae)
Friuli Venezia Giulia • Udine, Val Raccolana, road from Chiusaforte to Sella Nevea, 46°23’45”N, 13°26’02”E, 700 m, on bark of broad-leaved trees, 1997, leg. P. L. Nimis & M. Tretiach, TSB (31107).
A rare, cool-temperate, mainly western species with optimum in humid *Abies*-*Fagus* forests, but also occurring in humid sites of Tyrrhenian Italy within the Mediterranean belt.

Scytinium biatorinum (Nyl.) Otálora, P. M. Jørg. & Wedin (Collemataceae)
Trentino-Alto Adige • Bressanone, Val di Valles, trail to “Brixener Hütte”, 46°54’42”N, 11°37’19”E, 2240 m, on mica-shist, 7 July 2025, leg. et det. R. Kray, Hb. Kray (1408). • Bressanone, Val di Valles, at the beginning of Val di Valles, 46°52’52”N, 11°37’16”E, 1740 m, on mica-shist, 7 July 2025, leg. R. Kray, det. R. Kray & M. Schultz, Hb. Kray (1411).
Both collections ecologically deviate from this otherwise typically terricolous species of disturbed habitats. *Scytinium biatorinum*, as currently circumscribed, is probably not homogeneous (M. Schultz, pers. comm.), and it remains to be determined whether these ecological differences correspond to distinct phylogenetic lineages.

Scytinium imbricatum (P.M. Jørg.) Otálora, P.M. Jørg. & Wedin (Collemataceae)
Piemonte • Cuneo, Marguareis Natural Park, Pass between Cima di Pertegue (Cima di Pertegà) and Rocca del Fera, W above Upega, 44°08’50”N, 7°41’30”E, 2200 m, on calciferous soil, 20 July 2000, leg. P. L. Nimis & M. Tretiach (with J. Hafellner), det. P. L. Nimis, TSB (34599).
Emilia-Romagna • Bologna, Corno alle Scale, “Strada Forestale delle Malghe” above the tree line, 44°07’30”N, 10°49’00”E, 1598 m, on soil over rocks, 29 October 2025, leg. C. Pistocchi, det. C. Pistocchi & J. Nascimbene, Hb. Nascimbene (10154), Hb. Pistocchi (963).

On more or less calciferous ground in alpine grasslands; likely to occur throughout the Italian Alps, but formerly filed under pulvinate forms of *S. gelatinosum* or *S. lichenoides*. The record from Emilia-Romagna is the first one from the Northern Apennines.

Seirophora contortuplicata (Ach.) Frödén (Teloschistaceae)
Liguria • Imperia, Alpi Liguri Natural Park, ridge S above Monesi, W of Col Garezzo, 44°02'50"N, 7°46'22"E, 1850 m, on calciferous schists, 21 July 2000, leg. P. L. Nimis & M. Tretiach (with J. Hafellner), det. P. L. Nimis, TSB (33362).
On south-facing surfaces protected from rain (e.g. under overhangs) of calciferous rocks in the mountains. Not uncommon in the Alps and locally frequent along the eastern slopes of the Central Apennines.

Solorina bispora* var. *subspongiosa (Zschacke) Frey (Peltigeraceae)
Trentino-Alto Adige • Trento, Adamello-Brenta Natural Park, Val Gelada below Bocchetta dei Tre Sassi, 46°14'34"N, 10°53'04"E, 2350 m, on organic soil, 24 June 2022, leg. et det. C. Pistocchi and J. Nascimbene, Hb. Nascimbene (8906). • Trento, Adamello-Brenta Natural Park, near Passo del Grosté, 46°12'18"N, 10°53'47"E, 2395 m, on organic soil, 1 July 2022, leg. et det. C. Pistocchi & J. Nascimbene, Hb. Nascimbene (9411). • Trento, Adamello-Brenta Natural Park, Val Scura, 46°14'04"N, 10°56'41"E, 2033 m, on soil, 22 August 2024, leg. E. Betti, det. E. Auer & C. Pistocchi, Hb. Pistocchi (653). • Bolzano, Odle-Puez Natural Park, Altopiano di Crespeina, 46°34'18"N, 11°49'54"E, 2420 m, on basic soil, 22 June 2025, leg. et det. J. Nascimbene, Hb. Nascimbene (9839).
Emilia-Romagna • Modena, Alto Appennino Modenese Natural Park, Mt. Cimone (summit area), 44°11'40"N, 10°41'54"E, 2137 m, on soil, 4 August 2025, leg. C. Pistocchi, S. Girlanda & F. Zinato, det. C. Pistocchi & J. Nascimbene, Hb. Nascimbene (10095).
This is the morph with a dominant cyanobacterial photobiont in the *S. bispora*-group; on basic to subneutral soil at high elevations; not always distinguished, and probably more widespread, also in the Alps. The record from Emilia-Romagna is the first one from the Apennines.

Solorina octospora (Arnold) Arnold (Peltigeraceae)
Friuli Venezia Giulia • Udine, Natura 2000 Site-Monti Bivera e Clapsavon, surroundings of Sauris, Mt. Clapsavon, summit area, 46°26'15"N, 12°37'55"E, 2450 m, over thick soil layer on Triassic limestone, 28 July 1995, leg. et det. J. Hafellner, GZU (JH32701).
Valle d'Aosta • Aosta, Val Veny W of Courmayeur, ridge W above the Rifugio Elisabetta Soldini, 45°45'55"N, 6°50'05"E, 2250 m, on soil over Jurassic limestone, 30 July 2001, leg. J. Hafellner (with P. L. Nimis & M. Tretiach), det. J. Hafellner, GZU (JH75413).
A species of the *S. saccata*-group with the best developed thallus, and relatively small apothecia. The 8-spored asci are diagnostic. It settles on soil or overgrows plant remnants and mosses over calciferous substrata. The species has a holarctic distribution, in Europe with an arctic-alpine disjunction. In the Alps it is hardly observed below tree line and has the optimum from the alpine to the subnival belt. In Italy it was reported only for some northern regions, where virtual gaps are herewith closed.

Sporodictyon schaererianum A. Massal. (Verrucariaceae)
Veneto • Belluno, Pordoi, between Rifugio Vièl dal Pan and Rifugio Fredarola, 46°28'36"N, 11°49'19"E, 2458 m, on volcanic rocks, 8 August 2024, leg. et det. J. Nascimbene, Hb. Nascimbene (9490).
A mainly circum-arctic to boreal-montane lichen found on hard calciferous rocks, often on dolomite, but also on calcareous sandstone and schist in cold-humid situations, with optimum above tree line. For further details see Savić and Tibell (2009).

Staurothele fissa (Taylor) Zwackh (Verrucariaceae)
Friuli Venezia Giulia • Udine, Paluzza, above Malga Promosio in a small stream crossing the road, 46°35'36"N, 13°02'12"E, 1700 m, on acidic Palaeozoic rocks overgrowing *Hydropunctaria scabra*, 20 September 2022, leg. et det. J. Nascimbene, Hb. Nascimbene (7996).

A probably circumpolar lichen, amphibious in montane to alpine creeks, or growing on moist siliceous rocks; much rarer in the high Mediterranean mountains than in the Alps.

Staurothele frustulenta Vain. (Verrucariaceae)
Liguria • Imperia, Alpi Liguri Natural Park, just E below Upega on right bank of the creek Negrone (Corvo), 44°07'35"N, 7°44'10"E, 1260 m, on calciferous rocks, 19 July 2000, leg. P. L. Nimis & M. Tretiach (with J. Hafellner), det. P. L. Nimis, TSB (33552*)*.
On calcareous or basic siliceous rocks in open habitats, mostly below the subalpine belt.

Stereocaulon nanodes Tuck. (Stereocaulaceae)
Piemonte • Cuneo, Vallone di S. Anna SW above Vinadio by the road to Colle della Lombarda, 44°16'25"N, 7°08'00"E, 1340 m, on iron-rich siliceous rocks, 24 July 2000, leg. P. L. Nimis & M. Tretiach (with J. Hafellner), det. P. L. Nimis, TSB (33837). • Cuneo, Monviso Natural Park, N slopes of Monte Viso W above Crissolo, E above the northern end of Lago Fiorenza (S above Pian del Re), 44°41'50"N, 7°05'35"E, 2130 m, on scattered ophiolitic rock boulders, 25 July 2001, leg. J. Hafellner (with P. L. Nimis & M. Tretiach), det. J. Hafellner, GZU (JH87757).
Valle d'Aosta • Aosta, Val Veny W of Courmayeur, moraines E of Lago di Combal, 45°46'35"N, 6°52'10"E, 1980 m, on erratic boulder of iron-rich schist, 30 July 2001, leg. J. Hafellner (with P. L. Nimis & M. Tretiach), det. J. Hafellner, GZU (JH88600).
An arctic-alpine to boreal-montane, circumpolar lichen found on mineral-rich rocks, also under overhanging faces, on pebbles and large boulders, often on metal-rich substrata and in rather disturbed habitats in upland areas, reaching the nival belt; certainly more widespread in the Alps.

Synalissina cladodes (Tuck.) M. Schultz & M. Prieto (Lichinellaceae)
Trentino-Alto Adige • Trento, Adamello-Brenta Natural Park, near Cima Nana, 46°17'22"N, 10°54'45"E, 2370 m, on carbonatic soil, 28 July 2022, leg. et det. J. Nascimbene & C. Pistocchi, Hb. Nascimbene (9656). • Trento, Brenta Dolomites, Cima Nana, 46°17'01"N, 10°54'14"E, 2651 m, on carbonatic soil, 17 August 2022, leg. C. Pistocchi, det. C. Pistocchi & J. Nascimbene, Hb. Nascimbene (9661).
On more or less calciferous rocks with periodical percolation of water in cool situations; widespread in the Northern Hemisphere (Scandinavia, North America), but not common; rarely collected in the Alps, being only known from Switzerland, and recently found in the Alps of Veneto.

Tetramelas geophilus (Sommerf.) Norman (Caliciaceae)
Valle d'Aosta • Aosta, Passo del Piccolo San Bernardo, 45°41'47"N, 6°53'55"E, 2050 m, on calciferous rocks, September 1997, leg. et det. P. L. Nimis, TSB (29476).
A mainly boreal-montane to arctic-alpine, circumpolar lichen overgrowing terricolous and epilithic mosses on calciferous substrata, rarely corticolous, near and above tree line.

Tetramelas papillatus (Sommerf.) Kalb (Caliciaceae)
Liguria • Imperia, Alpi Liguri Natural Park, mountains N above Triora on the road to Col Garezzo, 44°02'50"N, 7°45'15"E, 1650 m, on soil and plant debris over sandstone, 21 July 2000, leg. P. L. Nimis & M. Tretiach (with J. Hafellner), det. P. L. Nimis, TSB (33479).
An arctic-alpine lichen found on terricolous bryophytes, mostly above tree line, reaching the nival belt in the Alps; it is related to *T. insignis*.

Thalloidima squamatum (Hoffm.) M. Westb. & Timdal (Ramalinaceae)
Trentino-Alto Adige • Trento. Adamello-Brenta Natural Park, below Sasso Rosso along Sentiero Costanzi, 46°16'41"N, 10°54'27"E, 2540 m, on carbonatic soil, 28 July 2022, leg. J. Nascimbene & C. Pistocchi, det. C. Pistocchi, Hb. Nascimbene (8405).

A recently-resurrected, apparently widespread species growing on moss cushions over calcareous rocks or directly on rock, rarely on soil. Italian material of *Th. sedifolium* should be checked against this species, which is likely to be quite common, especially in upland areas. For further details see Westberg et al. (2023).

Thalloidima tauricum Szatala (Ramalinaceae)
Piemonte • Cuneo, crest SW above Colle dell'Agnello, 44°40'58"N, 6°58'39"E, 2830 m, on calciferous soil, 25 July 2000, leg. P. L. Nimis & M. Tretiach (with J. Hafellner), det. P. L. Nimis, TSB (33180).
Liguria • Imperia, Alpi Liguri Natural Park, mountains N above Triora on the road to Col Garezzo, 44°02'50"N, 7°45'15"E, 1650 m, on soil and plant debris over sandstone, 21 July 2000, leg. P. L. Nimis & M. Tretiach (with J. Hafellner), det. P. L. Nimis, TSB (33477).
A mainly southern species with an Eurasiatic distribution, found on calciferous soil and in fine crevices of the rocks, often associated with cyanobacterial lichens when young, with a wide altitudinal range.

Thalloidima toninianum (A. Massal.) A. Massal. (Ramalinaceae)
Piemonte • Cuneo, ridge NW above Crissolo N above Pian di Melzè, S-exposed slopes, 44°42'15"N, 7°07'05"E, 1880 m, on calciferous schists, 25 July 2001, leg. P. L. Nimis & M. Tretiach (with J. Hafellner), det. P. L. Nimis, TSB (3534).
A mainly Mediterranean to submediterranean-montane species found on steeply inclined to slightly rain-sheltered seepage tracks of calcareous rocks, always in association with cyanobacterial colonies.

Thelidium incavatum Mudd (Verrucariaceae)
Piemonte • Cuneo, ridge SE above Colle Valcavera-Vallone dell'Arma, 44°22'40"N, 7°06'15"E, 2440 m, on calciferous schists, 23 July 2000, leg. P. L. Nimis & M. Tretiach (with J. Hafellner), det. P. L. Nimis, TSB (33967*)*.
On small calcareous pebbles close to the ground, usually in upland areas; probably more widespread, but overlooked.

Thelidium pertusatii (Garov.) Jatta (Verrucariaceae)
Lombardia • Bergamo, Orobie Bergamasche Natural Park, creek near Malga Gaffione, 46°02'06"N, 10°11'23"E, 1817 m, on siliceous sandstone, 5 August 2024, leg. G. Gheza, det. J. Nascimbene, Hb. Gheza (24080548).
An amphibious species on frequently wetted siliceous rocks in alpine rivers and irrigated rocks.

Thelidium subabsconditum Eitner (Verrucariaceae)
Veneto • Belluno, Dolomiti Bellunesi Ntl Park, Cima de Zità Sud, 46°16'16"N, 12°09'24"E, 2540 m, on carbonatic rocks, 2 July 2024, leg. et det. J. Nascimbene, Hb. Nascimbene (9428).
Widespread in the Alps (Nimis et al. 2018) on inclined surfaces of compact calciferous rocks in rather shaded, non-eutrophicated situations near or above tree line. Since it was not always distinguished from similar species, it has an apparently incomplete distribution in the Alps.

Thelidium ungeri Körb. (Verrucariaceae)
Piemonte • Cuneo, western ridge of Mt. Nebin ca. 1 Km E of Colle di Sampeyre, 44°32'40"N, 7°08'20"E, 2380 m, on calciferous rocks, 26 July 2000, leg. P. L. Nimis & M. Tretiach (with J. Hafellner), det. P. L. Nimis, TSB (32944).
On inclined surfaces of calciferous rocks in upland areas. Closely related to *T. pyrenophorum*, from which it differs in the thick, verrucose thallus, this species seems to be widespread in the Alps.

Thelopsis flaveola Arnold (Stictidaceae)
Trentino-Alto Adige • Trento, Adamello-Brenta Natural Park, near Passo del Grosté, 46°12'16"N, 10°53'54"E, 2480 m, on plant debris, 1 July 2022, leg. et det. J. Nascimbene & C. Pistocchi, Hb. Nascimbene (9412).

A mainly temperate species found on bark of ancient deciduous trees, but also, in the subalpine belt, at the base of old *Rhododendron* shrubs; to be looked for further in the Alps.

Toensbergia geminipara (Th. Fr.) T.Sprib. & Resl (*incertae sedis*)
Friuli Venezia Giulia • Udine, Mt. Fleons (Raudenspitz), along the trail on the crest W below the top, 46°38'45"N, 12°45'10"E, 2400 m, over bryophytes and plant remnants, 17 August 1996, leg. J. Hafellner (with M. Tretiach & A. Hafellner), det. J. Hafellner, GZU (JH39098).
Valle d'Aosta • Aosta, Gran Paradiso Ntl Park, near the trail from Pont to the Rifugio Vittorio Emanuele II, 45°30'50"N, 7°13'17"E, 2550 m, on plant remnants and bryophytes (dying *Polytrichum*), 31 July 2001, leg. J. Hafellner (with P. L. Nimis & M. Tretiach), det. J. Hafellner, GZU (JH87049).
For long time this species has been treated as either an *Ochrolechia* or a *Pertusaria*; however, its secondary chemistry is not found in these genera. A convincing solution for this taxonomic problem was found, when a close relationship with the type species of *Toensbergia* was demonstrated (Spribille et al. 2020). Both specimens cited here are sterile and contain alectorialic acid, responsible for the thalli turning pinkish to reddish after storage in the herbarium. This chemistry is unique among the terricolous-muscicolous sorediate crustose lichens found above tree line. The species shows a holarctic, arctic-alpine distribution and is also widespread in the Alps, but not common. In Alpine Italy, it had been reported from Friuli and Valle d'Aosta (Nimis et al. 2018), but without locality data. Herewith we fill these gaps.

Toensbergia leucococca (R.Sant.) Bendiksby & Timdal (Lecanoromycetidae inc. sed.)
Veneto • Belluno, Prealpi Bellunesi, Nevegal SE of Belluno, slopes exposed to NE, surroundings of the valley station of the chair-lift on Col Faverghera, 46°05'25"N, 12°16'40"E, 1030 m, on bark of *Alnus* sp., 31 August 2002, leg. et det. J. Hafellner, GZU (JH61083).
This epiphytic species, the type species of *Toensbergia*, is only known in the sterile state. In phylogenetic reconstructions, a sister position of *Toensbergia* to *Sporastatia* was shown (Bendiksby and Timdal 2013, Spribille et al. 2020). However, judging from morpho-anatomy and ecology, the assignment of the two genera in the same family (Sporastatiaceae) appears little convincing. The species settles on bark of both deciduous and coniferous trees under oceanic to suboceanic climates. With an overall holarctic distribution, in the Alps *Toensbergia leucococca* is more frequent in the marginal ranges, mostly in the montane belt. Its distribution in Italy is still insufficiently documented.

Toninia asiae-centralis (H. Magn.) Timdal (Ramalinaceae)
Piemonte • Cuneo, ridge NW above Crissolo N above Pian di Melzè, S-exposed slopes, 44°42'15"N, 7°07'05"E, 1880 m, on calciferous schists, 25 July 2001, leg. P. L. Nimis & M. Tretiach (with J. Hafellner), det. P. L. Nimis, TSB (35346).
On calciferous rocks and soil; despite the name, this subspecies is widespread also in Southern and Central Europe, with scattered outposts north to Greenland.

Toniniopsis verrucarioides (Nyl.) Kistenich, Timdal, Bendiksby & S. Ekman (Ramalinaceae)
Trentino-Alto Adige • Trento, Adamello-Brenta Natural Park, Val Gelada between Malga Mondifrà and Bocchetta dei Tre Sassi, 46°14'40"N, 10°52'00"E, 1870 m, on carbonatic rocks, 5 September 2022, leg. et det. J. Nascimbene, Hb. Nascimbene (8215).
Liguria • Imperia, Alpi Liguri Natural Park, just E below Upega on right bank of the creek Negrone (Corvo), 44°07'35"N, 7°44'10"E, 1260 m, on calciferous rocks, 19 July 2000, leg. P. L. Nimis & M. Tretiach (with J. Hafellner), det. P. L. Nimis, TSB (33570).
An arctic-alpine to cool-temperate lichen found in fissures and fine crevices of calcareous rocks in upland areas, often growing on species of *Placynthium* when young.

Trapeliopsis pseudogranulosa Coppins & P. James (Trapeliaceae)
Trentino-Alto Adige • Trento, trail between Lago dei Caprioli and Malga Alta, 46°17'40"N, 10°46'24"E, 1500 m, on organic soil, 31 July 2025, leg. et det. G. Gheza, Hb. Gheza (25073101). • Trento, trail between

Lago di Stablo and Malga Artuich, 46°16'45''N, 10°46'32''E, 1810 m, on organic soil, 31 July 2025, leg. et det. G. Gheza, Hb. Gheza (25073105).
Lombardia • Brescia, Valle delle Forme in Val Grigna, 45°53'38''N, 10°19'37''E, 1210 m, on organic soil, 6 August 2019, leg. et det. G. Gheza, Hb. Gheza. • Bergamo, trail between Forcellino and Bossico, 45°50'28''N, 10°03'44''E, 1140 m, on organic soil, 7 January 2022, leg. et det. G. Gheza, Hb. Gheza (22010736). • Bergamo, Orobie Bergamasche Natural Park, trail between Laghi Gemelli and Carona, 46°00'50''N, 9°46'58''E, 1430 m, on organic soil, 17 June 2022, leg. et det. G. Gheza, Hb. Gheza (22061711). • Brescia, Adamello Natural Park, Val Sozzine, 46°14'42''N, 10°32'22''E, 1417 m, on organic soil, 10 August 2022, leg. et det. G. Gheza, Hb. Gheza (22081004). • Bergamo, Orobie Bergamasche Natural Park, trail between Baita Brandelegno and Baita Napoleù, 46°00'45''N, 10°05'51''E, 1472 m, on organic soil, 17 August 2022, leg. et det. G. Gheza, Hb. Gheza (22081715). • Pavia, Hermitage of Sant'Alberto di Butrio, 44°51'15''N, 9°08'52''E, 640 m, on organic soil, 15 October 2022, leg. et det. G. Gheza, Hb. Gheza (22101503). • Bergamo, road between Villa Richetti and Colere, 45°58'15''N, 10°06'04''E, 1030 m, on mosses above a wall at the road edge, 5 January 2023, leg. et det. G. Gheza, Hb. Gheza (23010511). • Brescia, below Malga Creisa, 45°57'30''N, 10°07'43''E, 1420 m, on organic soil, 7 January 2023, leg. et det. G. Gheza, Hb. Gheza (23010701). • Bergamo, Natura 2000 Site-Boschi del Giovetto di Palìne, mule track between Prat de Bregn and Malga Costone, 45°57'59''N, 10°08'04''E, 1570 m, on organic soil, 23 April 2023, leg. et det. G. Gheza, Hb. Gheza (23042301). • Varese, trail between Mt. Chiusarella and Monte Martica, 45°52'52''N, 8°48'47''E, 900 m, 1 October 2023, on organic soil, leg. et det. G. Gheza, Hb. Gheza (23100110). • Brescia, Stelvio Ntl Park, above Sant'Apollonia, 46°17'42''N, 10°30'13''E, 1717 m, on soil, 7 August 2025, leg. et det. G. Gheza, Hb. Gheza (25080723). • Brescia, Val Moranda below Malga Dosso, 46°08'35''N, 10°15'53''E, 1665 m, on organic soil, 22 August 2025, leg. et det. G. Gheza, Hb. Gheza (25082200).
Piemonte • Biella, trail between Bocchetto Sessera and Alpe Montuccia, 45°39'56''N, 8°03'40''E, 1379 m, on organic soil, 5 October 2025, leg. et det. G. Gheza, Hb. Gheza (25100502). • Vercelli, Valsesia, Regina degli Angeli Chapel above Rainero di Rossa, 45°50'14''N, 8°07'17''E, 1123 m, on organic soil, 22 February 2026, leg. et det. G. Gheza, Hb. Gheza.
Easily recognizable by the green thallus with pale greenish soralia and scattered orange patches. Despite being widespread in the Alps outside Italy (Nimis et al. 2018), the records reported here are the first from the Italian Alps, where it is likely very widespread, but overlooked. It is listed as "vulnerable" in the Red List of terricolous lichens of Italy (Gheza et al. 2022).

Umbilicaria nylanderiana (Zahlbr.) H. Magn. (Umbilicariaceae)
Veneto • Belluno, Arabba, N-facing slope of Porta Vescovo, start of the via ferrata, 46°28'24''N, 11°52'24''E, 2588 m, on volcanic rocks, 1 August 2025, leg. et det. J. Nascimbene, Hb. Nascimbene (10225).
An arctic-alpine, probably circumpolar lichen found on the top of isolated siliceous boulders, most frequent in the Alps above tree line, but reaching south to the mountains of Calabria.

Umbilicaria subglabra (Nyl.) Harm. (Umbilicariaceae)
Emilia-Romagna • Modena, Alto Appennino Modenese Natural Park, Monte Cimone between Salto della Capra and Rifugio Pian Cavallaro, 44°12'04''N, 10°42'36''E, 1820 m, on subacidic arenaceous rocks, 21 October 2022, leg. et det. J. Nascimbene, Hb. Nascimbene (8017). • Parma, Bedonia, Monte Ragola, 44°35'13''N, 9°32'18''E, 1662 m, on rocks, 24 November 2023, leg. D. Ivan, det. E. Auer & G. Gheza, Hb. Nascimbene (9726).
On steeply inclined to horizontal, exposed surfaces of siliceous rocks, often at the top of large boulders; certainly widespread throughout the Alps. These are the first record from the Apennines.

Varicellaria rhodocarpa (Körb.) Th. Fr. (Ochrolechiaceae)
Piemonte • Cuneo, Monviso Natural Park, N slopes of Monte Viso W above Crissolo, E above the N end of Lago Fiorenza (S above Pian del Re), 44°41'50''N, 7°05'35''E, 2130 m, on plant remnants, 25 July 2001, leg. J. Hafellner (with P. L. Nimis & M. Tretiach), det. J. Hafellner, GZU (JH87783).

We report here a fertile specimen of this rather conspicuous alpine crustose lichen. Its ascomata immersed in the white to cream-coloured thallus, however, are very inconspicuous and may be mistaken as poorly developed soralia. The huge glossy hyaline ascospores protruding from the hymenia and accumulating on the ascoma surface are occasionally visible under the dissecting microscope. The holarctic species (with an enclave in southernmost South America) has an arctic-alpine distribution in Europe. In the Alps it is apparently rarer in the western parts.

Variospora australis (Arnold) Arup, Søchting & Frödén (Teloschistaceae)
Piemonte • Cuneo, ridge SE above Colle Valcavera-Vallone dell'Arma, 44°22'40"N, 7°06'15"E, 2440 m, on calciferous schists, 23 July 2000, leg. P. L. Nimis & M. Tretiach (with J. Hafellner), det. P. L. Nimis, TSB (33944).
On exposed calciferous rocks near or above tree line, e.g., on the top of large, isolated boulders and on steeply inclined to vertical surfaces.

Variospora paulii (Poelt) Arup, Søchting & Frödén (Teloschistaceae)
Veneto • Belluno, Val di Gares, near Cascata di Gares Alta, 46°18'12"N, 11°52'20"E, 1540 m, on calcareous rocks, 12 August 2023, leg. et det. J. Nascimbene, Hb. Nascimbene (8290).
On weakly to steeply inclined surfaces of limestone and marl slates in upland areas; known from various Eurasian orobiomes, from Greenland, and from many localities in the Alps, but rather rare or poorly collected.

Verrucaria alpicola Zschacke (Verrucariaceae)
Lombardia • Bergamo, Orobie Bergamasche Natural Park, trail between Nona and the Gleno Dam, 46°00'35"N, 10°04'09"E, 1454 m, on siliceous sandstone, 17 August 2022, leg. G. Gheza, det. J. Nascimbene, Hb. Gheza (22081752).
A typically sub-aquatic species which often occurs in the splash water zone in streams, but also at temporarily inundated sites in springs, both on calcareous and on siliceous rocks, in sunny to moderately shaded sites, mostly in upland areas; probably more widespread, at least in the Alps.

Verrucaria anziana Garov. (Verrucariaceae)
Trentino-Alto Adige • Trento, Adamello-Brenta Natural Park, near Lago Scuro, 46°12'36"N, 10°34'29"E, 2680 m, on tonalite, 9 September 2023, leg. et det. J. Nascimbene, Hb. Nascimbene (8927), GenBank Acc.Nr.: PZ094139. • Paneveggio - San Martino Natural Park, streamlet below exit from the lower lake of the Laghetti di Colbricon, 46°17'05"N, 11°45'55"E, 1910 m, on carbonatic rocks, 2 July 2025, leg. et det. H. Thüs, STU (SMNS-STU-F-0005139), GenBank Acc.Nr.: PZ053819.
This species had long been treated as a synonym of *V. latebrosa*, but with the revision of Orange (2020) it became clear that it is a genetically distinct species. Typical specimens with fully immersed perithecia (like our specimen from San Martino) can be separated morphologically from similar taxa of the *V. aethiobola* group, while for thalli with more or less protruding perithecia (such as in the specimen 8927 from Hb. Nascimbene) DNA-barcoding is required for an unambiguous identification. It usually grows on siliceous rocks by streams, rivers and lakes, being known in Europe from the Alps, the British Isles and Scandinavia, but sequenced specimens are also known from Alaska (Spribille et al. 2020) and the species might have a circumboreal distribution.

Verrucaria funckii (Spreng.) Zahlbr. (Verrucariaceae)
Lombardia • Bergamo, Orobie Bergamasche Natural Park, trail between Nona and the Gleno Dam, 46°00'35"N, 10°04'09"E, 1454 m, on siliceous sandstone, 17 August 2022, leg. G. Gheza, det. J. Nascimbene, Hb. Gheza (22081751). • Bergamo, Orobie Bergamasche Natural Park, Laghetti di San Carlo, 46°03'22"N, 10°09'36"E, 2271 m, on siliceous rocks (schist), 26 August 2022, leg. G. Gheza & L. Di Nuzzo, det. J. Nascimbene, Hb. Gheza (22082623). • Bergamo, Orobie Bergamasche Natural Park, Baita di Varro, 46°01'23"N, 10°06'25"E, 2044 m, on siliceous sandstone, 20 July 2024, leg. et det. G. Gheza & L. Di Nuzzo, Hb. Gheza (24072058).

Among freshwater Verrucariaceae, this is one of the few species which are usually found in permanently submerged conditions, more rarely in the splash zone of water courses or on deeply shaded stream banks, always on siliceous substrata. It is a typical element of springs and clear headwaters, where it can dominate the benthic community. Probably much more widespread in the Alps.

Verrucaria praetermissa (Trevis.) Anzi (Verrucariaceae)
Lombardia • Bergamo, Orobie Bergamasche Natural Park, Vilminore di Scalve, trail between Bueggio and Diga del Gleno, 46°00'29''N, 10°04'22''E, 1228 m, on siliceous sandstone, 17 August 2022, leg. G. Gheza, det. J. Nascimbene & G. Gheza, Hb. Gheza (22081755), Hb. Nascimbene (8014). • Varese, Lago di Ganna Nature Reserve, Lago di Ganna, 45°53'26''N, 8°49'46''E, 460 m, on porphyry, 20 May 2023, leg. G. Gheza, det. G. Gheza & J. Nascimbene, Hb. Gheza (23052002). • Brescia, Adamello Natural Park, Deria, 46°02'39''N, 10°21'22''E, 610 m, on siliceous schists, 1 May 2024, leg. et det. G. Gheza, Hb. Gheza (24050103). • Bergamo, Orobie Bergamasche Natural Park, Val del Vò above Ronco, 46°00'59''N, 10°08'25''E, 1130 m, on siliceous sandstone, 19 August 2024, leg. et det. G. Gheza, Hb. Gheza (24081920).
Emilia-Romagna • Bologna, Corno alle Scale Natural Park, Madonna dell'Acero along Fosso della Canella, 44°09'09''N, 10°50'35''E, 1050 m, on sandstone, 19 June 2024, leg. et det. J. Nascimbene, Hb. Nascimbene (9052), ITS-GenBank Acc.Nr: PZ094141.
A probably circumboreal freshwater species, typically submerged only for short periods (long-term inundated only in cold springs), often found in the splash water zone along creeks, on mineral-rich siliceous rocks with alkaline to neutral or only slightly acidic surfaces, but also on acidic rocks when irrigated by subneutral to alkaline water, and on calcareous substrata; perhaps more widespread in the Alps. Nimis (1993) stated that, according to Zschacke (1933), the specimens from Lombardia cited and distributed by Anzi as *Verrucaria praetermissa* (Lich. Lang. n. 244) belonged instead to *Verrucaria latebrosa* Körb., but the interpretation of the latter name has changed with the revision of Orange (2020), and molecular data are now required for unambiguous identification. Later, Nimis (2016) reported from Lombardia the record by Jatta (1909-11), which however refers to specimens by Anzi as well. Those presented here are therefore the first reliable records from Lombardia.

Verrucula coccinearia (Zehetl.) Nav.-Ros. & Cl. Roux (Verrucariaceae)
Piemonte • Cuneo, ridge SE above Colle Valcavera-Vallone dell'Arma, 44°22'40''N, 7°06'15''E, 2440 m, on calciferous schists, 23 July 2000, leg. P. L. Nimis & M. Tretiach (with J. Hafellner), det. P. L. Nimis, TSB (33973).
On the thalli of *Caloplaca coccinea* in upland areas; overlooked and certainly more widespread in the Alps.

Verrucula biatorinaria (Zehetl.) Nav.-Ros. & Cl. Roux (Verrucariaceae)
Piemonte • Cuneo, ridge W above Colle del Vallonetto (Vallone dell'Arma), 44°23'10''N, 7°07'00''E, 2500 m, on calcareous rocks, 23 July 2000, leg. P. L. Nimis & M. Tretiach (with J. Hafellner), det. P. L. Nimis, TSB (34037).
A lichenicolous lichen growing on the thalli of *Calogaya biatorina*; certainly more widespread in the Alps and along the Apennines.

Verseghya thysanophora (R.C. Harris) S.Y. Kondr., Lőkös, Farkas & Hur (Lecanoraceae)
Trentino-Alto Adige • Trento, Adamello-Brenta Natural Park, Val Brenta, 46°11'32''N, 10°50'50''E, 1320 m, on bark of *Abies alba*, 9 August 2022, leg. G. Gheza & C. Pistocchi, det. H. Mayrhofer, Hb. Gheza (22080915).
On bark in humid deciduous forests. This is a sorediate, mostly sterile epiphytic species described from North America (Tønsberg 1992, Harris et al. 2000), which has been often confused with sterile specimens of the mainly epilithic *Haematomma ochroleucum* var. *ochroleucum*. Both species, when sterile, are extremely similar, and also have a similar chemistry, as they produce atranorin, usnic acid, and zeorin, but *V. thysanophora* also contains a characteristic set of terpenoids ("*Thysanophora*-unknowns") and (at least in European specimens) lacks porphyrilic acid, which is present in *H. ochroleucum* (Kukwa and Motiejūnaitė 2005). Several earlier records of *H. ochroleucum* from the Alps could actually refer to *V. thysanophora*.

Violella fucata (Stirt.) T. Sprib. (Tephromelataceae)
Veneto • Belluno, Nevegal SE of Belluno, slopes exposed to NE, surroundings of the valley station of the chair-lift on Col Faverghera, 46°05'25''N, 12°16'40''E, 1030 m, on rotten stumps (wood, bark remnants), 31 August 2002, leg. et det. J. Hafellner, GZU (JH61089).
Lombardia • Brescia, Val di Corteno, along the road from Edolo to Aprica, W of Le Fucine, along the Torrente Ogliolo di Edolo close to the left stream bank, 46°09'55''N, 10°12'30''E, 1025 m, on bark of *Alnus incana*, 1 June 1975, leg. et det. J. Hafellner, GZU (JH770).
Liguria • Imperia, Alpi Liguri Natural Park, just E below the village Upega, on southern (right) bank of the stream Negrone (Corvo torrent), 44°07'35''N, 7°44'10''E, 1260 m, on bark of *Fagus*, 19 July 2000, leg. J. Hafellner (with P. L. Nimis & M. Tretiach), det. J. Hafellner, GZU (JH84445).
Violella fucata settles on acidic bark and wood and a decline of populations along with increasing eutrophication has been observed. It is widely distributed in the holarctic region, as a forest species from the northern boreal to the nemoral-montane zone (in Asia with enclaves in the Himalayan range). It is also widespread in the Alps (Nimis et al. 2018), its rarity in the Italian Alps being likely due to undercollecting. The presence of black basidiomata of the lichenicolous fungus *Tremella lichenicola* may confirm the identification.

Xanthocarpia ferrarii (Bagl.) Frödén, Arup & Søchting (Teloschistaceae)
Liguria • Imperia, Alpi Liguri Natural Park, slopes SW above the village Monesi, 44°04'20''N, 7°44'10''E, 1630 m, on calciferous rocks, 18 July 2000, leg. P. L. Nimis & M. Tretiach (with J. Hafellner), det. P. L. Nimis, TSB (33069).
A temperate early coloniser of mortar walls, gypsum outcrops and other calciferous, often man-made, soft substrata at relatively low elevations.

Xylographa parallela (Ach:Fr.) Fr. (Xylographaceae)
Liguria • Imperia, Alpi Liguri Natural Park, just E below Upega on right bank of the creek Negrone (Corvo), 44°07'35''N, 7°44'10''E, 1260 m, on wood, 19 July 2000, leg. P. L. Nimis & M. Tretiach (with J. Hafellner), det. P. L. Nimis, TSB (33648). • Imperia, *ibidem*, 19 July 2000, leg. J. Hafellner (with P. L. Nimis & M. Tretiach), det. J. Hafellner, GZU (JH87536).
A cool-temperate to boreal-montane, circumpolar species found on hard wood, on poles, fences and on flanks of decorticated boles, especially of conifers.

Xylographa vitiligo (Ach.) J.R. Laundon (Xylographaceae)
Liguria • Imperia, Alpi Liguri Natural Park, just E below Upega on right bank of the creek Negrone (Corvo), 44°07'35''N, 7°44'10''E, 1260 m, on wood, 19 July 2000, leg. P. L. Nimis & M. Tretiach (with J. Hafellner), det. P. L. Nimis, TSB (33605).
Veneto • Belluno, Casera Razzo 1 Km from the border between Friuli and Veneto, 46°28'36''N, 12°37'14''E, 1750 m, on wood of *Larix*, 20 September 1986, leg. et det. P. L. Nimis, TSB (1966).
A mainly boreal-montane species found on decaying, decorticated but still hard stumps, mostly of conifers, especially near the base, or on fallen trunks, with optimum near tree line.

References

Ahti T (1998) A revision of *Cladonia stricta*. Folia Cryptogamica Estonica 32: 5–8.

Baruffo L, Zedda L, Elix JA, Tretiach M (2006) A revision of the lichen genus *Lepraria* s.lat. in Italy. Nova Hedwigia 83: 387–430. https://doi.org/10.1127/0029-5035/2006/0083-0387

Bendiksby M, Timdal E (2013) Molecular phylogenetics and taxonomy of *Hypocenomyce* sensu lato (Ascomycota: Lecanoromycetes): Extreme polyphyly and morphological/ecological convergence. TAXON 62: 940–956. https://doi.org/10.12705/625.18

Bertrand M, Roux C (2013) *Aspicilia spermatomanes* (Nyl.) Maheu et Gillet en Corse. Bull. Ass. fr. lichénologie 38: 87–98.

Breuss O (2012) Zur Verbreitung von *Psoroma tenue* var. *boreale* (lichenisierte Ascomycota, Pannariaceae) in den Alpen. Stapfia 97: 169-173.

Breuss O, Berger F (2012) Die Validierung von *Verrucaria finitima* und Bemerkungen über den Formenkreis von *Verrucaria tristis* (lichenisierte Ascomyceten, Verrucariaceae). Österreichische Zeitschrift für Pilzkunde 21: 117–126.

Cecconi E, Pellegrini E, Micai G, Ongaro S, Kaci MA, Pisuttu C, Carniel FC, Nali C, Tretiach M (2025) Different altitudinal distributions of the two chemical varieties of *Pseudevernia furfuracea* collide with the conserved physiological response of transplanted lichens. Environmental and Experimental Botany 237: 106186. https://doi.org/10.1016/j.envexpbot.2025.106186

Černajová I, Steinová J, Škvorová Z, Škaloud P (2022) The curious case of *Cladonia luteoalba*: no support for its distinction. The Lichenologist 54: 345–354. https://doi.org/10.1017/S002428292200024X

Czeika H, Czeika G (2007) *Placynthium* in den Alpen und Karpaten sowie in benachbarten Gebieten. Herzogia 20: 29–51.

Degelius G (1954) The lichen genus *Collema* in Europe. Morphology, taxonomy, ecology. Symbolae Botanicae Upsaliensis 13: 1–499.

Esslinger TL (1978) Studies in the lichen family Physciaceae. II The genus *Phaeophyscia* in North America. Mycotaxon 7: 283–320. https://doi.org/10.5962/p.417856

Frisch A, Ohmura Y, Holien H, Bendiksby M (2022) A phylogenetic survey of the ascomycete genus *Arthrorhaphis* (Arthrorhaphidaceae, Lecanoromycetes) including new species in *Arthrorhaphis citrinella* sensu lato. TAXON 71: 936–962. https://doi.org/10.1002/tax.12718

Fryday AM, Printzen C, Ekman S (2014) *Bryobilimbia*, a new generic name for *Lecidea hypnorum* and closely related species. The Lichenologist 46: 25–37. https://doi.org/10.1017/S0024282913000625

Gheza G (2018) Addenda to the lichen flora of the Ticino river valley (western Po Plain, Italy). Natural History Sciences 5. https://doi.org/10.4081/nhs.2018.381

Gheza G (2019) The macrolichens of Val di Scalve (northern Italy) and the first record of *Parmelia pinnatifida* in Italy. Webbia 74: 307–315.

Gheza G (2020) I licheni terricoli degli ambienti aperti aridi della pianura piemontese. Rivista piemontese di Storia naturale 41: 147–155.

Gheza G, Nascimbene J (2024) Lichens from the *aurifodinae* of the upper Ticino river valley (N Italy). Natural History Sciences 11. https://doi.org/10.4081/nhs.2024.734

Gheza G, Di Nuzzo L, Nimis PL, Benesperi R, Giordani P, Vallese C, Nascimbene J (2022) Towards a Red List of the terricolous lichens of Italy. Plant Biosystems 156: 824–825. https://doi.org/10.1080/11263504.2022.2065379

Hafellner J (2010) Contributions to a revision of lichenized, phaeospored species of *Polyblastia* coll., mainly in Central European mountains. Bibliotheca Lichenologica 104: 117–141.

Hafellner J, Türk R (2016) Die lichenisierten Pilze Österreichs - eine neue Checkliste der bisher nachgewiesenen Taxa mit Angaben zur Verbreitung und Substratökologie. Stapfia 104: 1–216.

Harris RC, Brodo IM, Tønsberg T (2000) *Lecanora thysanophora* , a Common Leprose Lichen in Eastern North America. The Bryologist 103: 790–793. https://doi.org/10.1639/0007-2745(2000)103%255B0790:LTACLL%255D2.0.CO;2

Heiðmarsson S (2017) *Dermatocarpon*. In: Nordic Lichen Flora. Nordic Lichen Society, Sweden, Volume 6, 19–25.

Hertel H (2006) World distribution of species of *Lecidea* (Lecanorales) occurring in Central Europe. In: Lackovičová A, Guttová A, Lisická E, Lizoň P (Eds), Central European Lichens: Diversity and Threat. Mycotaxon Ltd., Ithaca, NY, 19–74.

Jørgensen PM (2004) *Psoroma tenue* var. *boreale*, an overlooked, widespread, arctic-alpine lichen. Graphis scripta 15: 60-64.

Jørgensen PM, Schultz M, Guttová A (2013) Validation of *Anema tumidulum* (Lichinaceae, Lichenized Ascomycota), A Widespread Cyanophilic Lichen. Herzogia 26: 1–7. https://doi.org/10.13158/heia.26.1.2013.1

Kashiwadani H (1975) The genera *Physcia*, *Physconia*, and *Dirinaria* (lichens) of Japan. Ginkgoana 3: 1–77.

Knudsen K, Kocourková J (2016) *Acarospora sphaerosperma* (Acarosporaceae), new for Europe and the Czech Republic, and *Acarospora irregularis*, new for Austria. Herzogia 29: 465–472.

Knudsen K, Kocourková J, Nordin A (2014) Conspicuous similarity hides diversity in the *Acarospora badiofusca* group (Acarosporaceae). The Bryologist 117: 319–328. https://doi.org/10.1639/0007-2745-117.4.319

Košuthová A, Westberg M, Wedin M (2022) A revision of the *Rostania occultata* (*Collemataceae*) complex in Fennoscandia. The Lichenologist 54: 13–24. https://doi.org/10.1017/S0024282921000487

Kukwa M, Motiejūnaitė J (2005) Notes on *Haematomma ochroleucum* and *Lecanora thysanophora* lichens in Lithuania [Pastabos apie dvi kerpių rūšis – *Haematomma ochroleucum* ir *Lecanora thysanophora* - Lietuvoje]. Botanica Lithuanica 11: 247–249.

Martellos S (2003) The distribution of the two chemical varieties of the lichen *Pseudevernia furfuracea* in Italy. Plant Biosystems - An International Journal Dealing with all Aspects of Plant Biology 137: 29–33. https://doi.org/10.1080/11263500312331351301

Mayrhofer H, Poelt J (1979) Die saxicolen Arten der Flechtengattung *Rinodina* in Europa. Cramer, Vaduz, 186 pp.

McCune B, Arup U, Breuss O, Di Meglio E, Di Meglio J, Esslinger TL, Magain N, Miadlikowska J, Miller AE, Muggia L, Nelson PR, Rosentreter R, Schultz M, Sheard JW, Tønsberg, Tor, Walton J (2018) Biodiversity and ecology of lichens of Katmai and Lake Clark National Parks and Preserves, Alaska. Mycosphere 9: 859–930. https://doi.org/10.5943/mycosphere/9/4/10

Myllys L, Svensson M, Kantvilas G, Sérusiaux E, Van Den Boom P, Aptroot A, Cezanne R, Eichler M, Pino-Bodas R, Printzen C, Pykälä J, Thor G, Westberg M, Weber L, Kantelinen A (2026) The first comprehensive multilocus phylogeny of the lichenized genus *Micarea* (Ectolechiaceae, Ascomycota). TAXON 75: e70087. https://doi.org/10.1002/tax.70087

Nascimbene J (2005). Lichenological studies in northern Italy: new records for Trentino-Alto-Adige. Gredleriana 5: 39-44.

Nascimbene J, Gheza G, Hafellner J, Mayrhofer H, Muggia L, Obermayer W, Thor G, Nimis PL (2021) Refining the picture: new records to the lichen biota of Italy. MycoKeys 82: 97–137. https://doi.org/10.3897/mycokeys.82.69027

Nimis PL (1993) The lichens of Italy: an annotated catalogue. Mus. Reg. Sci. Nat., Torino, Monogr. XII, 897 pp.

Nimis PL (2016) The lichens of Italy. A second annotated catalogue. EUT Edizioni Università di Trieste, Trieste, 740 pp.

Nimis PL (2026) ITALIC - The Information System on Italian Lichens. Version 8.0. University of Trieste, Dept. of Biology. https://dryades.units.it/italic. All data are released under a CC BY-SA 4.0 licence.

Nimis PL, Hafellner J, Roux C, Clerc P, Mayrhofer H, Martellos S, Bilovitz PO (2018) The lichens of the Alps–an annotated checklist. MycoKeys: 1. 10.3897/mycokeys.31.23658

Orange A (2020) The *Verrucaria aethiobola* group (lichenised Ascomycota, Verrucariaceae) in North-west Europe. Phytotaxa 459. https://doi.org/10.11646/phytotaxa.459.1.1

Orange A, Earland-Bennett PM, Hitch CJB, Powell M (2017) A new leprose *Leprocaulon* (Ascomycota, *Leprocaulales*) from Great Britain. The Lichenologist 49: 183–188. https://doi.org/10.1017/S0024282917000093

Otálora MAG, Wedin M (2013) *Collema fasciculare* belongs in *Arctomiaceae*. The Lichenologist 45: 295–304. https://doi.org/10.1017/S0024282912000849

Otte V (2007) Flechten, lichenicole Pilze und Moose aus dem Nordwest- Kaukasus – zweiter Nachtrag. Herzogia 20: 221–237.

Poelt J (1979) Plantae Graecenses Jahrgang 4: 1-51. In: Poelt J (Ed.), Plantae Graecenses. Institut für Systematische Botanik, Universität Graz, Graz, 1–51.

Poelt J, Döbbeler P (1975) *Lecidea margaritella*, a liverwort-associated lichen and its occurrence in Central Europe. Herzogia 3: 327–334. https://doi.org/10.1127/herzogia/3/1975/327

Prieto M, Wedin M, Schultz M (2024) Phylogeny, evolution and a re-classification of the Lichinomycetes. Studies in Mycology 109: 595–655. https://doi.org/10.3114/sim.2024.109.09

Pykälä J, Kantelinen A, Myllys L (2024) Seven new species of *Verrucaria* from calcareous and siliceous rocks of Finland. The Lichenologist 56: 259–272. https://doi.org/10.1017/S002428292400029X

Ravera S, Puglisi M, Vizzini A, Totti C, Arosio G, Benesperi R, Bianchi E, Boccardo F, Briozzo I, Dagnino D, De Giuseppe AB, Dovana F, Di Nuzzo L, Fascetti S, Gheza G, Giordani P, Malíček J, Mariotti MG, Mayrhofer H, Minuto L, Nascimbene J, Nimis PL, Martellos S, Passalacqua NG, Pittao E, Potenza G, Puntillo D, Rosati L, Sicoli G, Spitale D, Tomaselli V, Trabucco R, Turcato C, Vallese C, Zardini M (2019) Notulae to the Italian flora of algae, bryophytes, fungi and lichens: 8. Italian Botanist 8: 47–62. https://doi.org/10.3897/italianbotanist.8.48263

Roux C, coll. (2014) Catalogue des lichens et champignons lichénicoles de France métropolitaine. Éditions Henry des Abbayes, Fougères, 1525 pp.

Roux C, Bertrand M, Poumarat S, Uriac P (2022) Quelques espèces nouvelles saxicoles–calcifuges de *Lecanora* du groupe *polytropa* (Ascomycota, Lecanoraceae) découvertes en France. Bulletin de la Société linnéenne de Provence 73: 79–120.

Roux C, Magain N, Poumarat S, Archambaux P, Uriac P (2025a) *Leprocaulon pseudocalcicola* Cl. Roux et Magain sp. nov.(Ascomycota, Leprocaulaceae). Bulletin de la Société Linnéenne de Provence 76.

Roux C, Poumarat S, Monnat J-Y, Farou J-L, Boissière J-C, Diederich P, Derrien M-C, Van Haluwyn C, Gonnet D, Esnault J, Carlier G, Houmeau J-M, Lagrandie J, Pinault P, Bauvet C, Masson D, Wirth V, Gardiennet A, Beudin T, Daval G, Mahé L (2025b) Catalogue des lichens et champignons lichénicoles de France métropolitaine. 4th ed. 2015 pp.

Savić S, Tibell L (2008) *Atla*, a new genus in the Verrucariaceae (Verrucariales). The Lichenologist 40: 269–282. https://doi.org/10.1017/S0024282908007512

Savić S, Tibell L (2009) Taxonomy and species delimitation in Sporodictyon (Verrucariaceae) in Northern Europe and the adjacent Arctic—reconciling molecular and morphological data. TAXON 58: 585–605. https://doi.org/10.1002/tax.582022

Scheidegger C (1987) *Buellia uberior* und *B. miriquidica* (Physciaceae, Lecanorales), zwei lichenicole Krustenflechten auf *Schaereria tenebrosa*. Bot. Helvetica 97: 99–116. https://doi.org/10.5169/SEALS-67858

Schmull M, Miadlikowska J, Pelzer M, Stocker-Wörgötter E, Hofstetter V, Fraker E, Hodkinson BP, Reeb V, Kukwa M, Lumbsch HT, Kauff F, Lutzoni F (2011) Phylogenetic affiliations of members of the heterogeneous lichen-forming fungi of the genus *Lecidea* sensu Zahlbruckner (Lecanoromycetes, Ascomycota). Mycologia 103: 983–1003. https://doi.org/10.3852/10-234

Schutte JA (1983) Distribution Patterns of Foliose and Fruticose Lichens of Eastern Iowa. The Bryologist 86: 117. https://doi.org/10.2307/3243176

Sheard JW (2010) The lichen genus *Rinodina* (Ach.) Gray (Lecanoromycetidae, Physciaceae) in North America, North of Mexico. NRC Research Press, Ottawa, 246 pp.

Spribille T, Fryday AM, Pérez-Ortega S, Svensson M, Tønsberg T, Ekman S, Holien H, Resl P, Schneider K, Stabentheiner E, Thüs H, Vondrák J, Sharman L (2020) Lichens and associated fungi from Glacier Bay National Park, Alaska. The Lichenologist 52: 61–181. https://doi.org/10.1017/S0024282920000079

Starosta J, Svoboda D (2020) Genetic variability in the *Physconia muscigena* group (Physciaceae, Ascomycota) in the Northern Hemisphere. The Lichenologist 52: 305–317. https://doi.org/10.1017/S0024282920000134

Stenroos S (1990) *Cladonia luteoalba* − an enigmatic *Cladonia*. Karstenia 30: 27–32. https://doi.org/10.29203/ka.1990.278

Stenroos S, Huhtinen S, Lesonen A, Palice Z, Printzen C (2009) *Puttea*, gen. nov., erected for the enigmatic lichen *Lecidea margaritella*. The Bryologist 112: 544–557. https://doi.org/10.1639/0007-2745-112.3.544

Thiers B (2016) Index Herbariorum: A global directory of public herbaria and associated staff. New York Botanical Garden's Virtual Herbarium. https://sweetgum.nybg.org/science/ih/.

Thüs H, Schultz M (2009) Fungi: Lichens 1. In: Süßwasserflora Von Mitteleuropa. Spektrum Akademischer Verlag, Heidelberg, 1–223.

Tønsberg T (1992) The sorediate and isidiate, corticolous, crustose lichens in Norway. Sommerfeltia 14: 1–336. https://doi.org/10.2478/som-1992-0002

Tretiach M, Hafellner J (2000) Lichens and lichenicolous fungi of Mt. Fleons (Carnic Alps, Italy). I. Epilithic and epigaeic species. Herzogia 14: 99–111.

Tuckerman E (1860) Four Hundred and Seventy-Eighth Meeting. March 13, 1860. Monthly Meeting; Observations on North American and Some Other Lichenes; On the Difficulty of Identifying Plants and Animals Mentioned by Ancient Greek Authors. Proceedings of the American Academy of Arts and Sciences 4: 383. https://doi.org/10.2307/20021247

Türk R, Hafellner J (1993) Flechten im Nationalpark Hohe Tauern – Kärntner Anteil (Österreich). Carinthia II 183: 723–757.

Westberg M, Ekman S, Briegel-Williams L, Fernandez-Brime S, Wedin M, Timdal E (2023) *Thalloidima squamatum* comb. nov.: a distinct and common but overlooked lichen in Europe. Plant and Fungal Systematics 68: 353–363. 10.35535/pfsyst-2023-0019.

Zschacke H (1933) Epigloeaceae, Verrucariaceae und Dermatocarpaceae. In: Zahlbruckner A (Ed.), Dr. L. Rabenhorst's Kryptogamen-Flora von Deutschland, Österreich und der Schweiz. Akademische Verlagsgesellschaft, Leipzig, 44–695.
